# HIV-1 Tat-mediated astrocytic amyloidosis involves the HIF-1α/lncRNA BACE1-AS axis

**DOI:** 10.1371/journal.pbio.3000660

**Published:** 2020-05-26

**Authors:** Susmita Sil, Guoku Hu, Ke Liao, Fang Niu, Shannon Callen, Palsamy Periyasamy, Howard S. Fox, Shilpa Buch

**Affiliations:** Department of Pharmacology and Experimental Neuroscience, University of Nebraska Medical Center, Omaha, Nebraska, United States of America; Centre International de Recherche en Infectiologie (CIRI), FRANCE

## Abstract

Increased life expectancy of patients diagnosed with HIV in the current era of antiretroviral therapy is unfortunately accompanied with the prevalence of HIV-associated neurocognitive disorders (HANDs) and risk of comorbidities such as Alzheimer-like pathology. HIV-1 transactivator of transcription (Tat) protein has been shown to induce the production of toxic neuronal amyloid protein and also enhance neurotoxicity. The contribution of astrocytes in Tat-mediated amyloidosis remains an enigma. We report here, in simian immunodeficiency virus (SIV)+ rhesus macaques and patients diagnosed with HIV, brain region–specific up-regulation of amyloid precursor protein (APP) and Aβ (40 and 42) in astrocytes. In addition, we find increased expression of β-site cleaving enzyme (BACE1), APP, and Aβ in human primary astrocytes (HPAs) exposed to Tat. Mechanisms involved up-regulation of hypoxia-inducible factor (HIF-1α), its translocation and binding to the long noncoding RNA (lncRNA) BACE1‐antisense transcript (BACE1-AS), resulting, in turn, in the formation of the BACE1-AS/BACE1 RNA complex, subsequently leading to increased BACE1 protein, and activity and generation of Aβ-42. Gene silencing approaches confirmed the regulatory role of HIF-1α in BACE1-AS/BACE1 in Tat-mediated amyloidosis. This is the first report implicating the role of the HIF-1α/lncRNABACE1-AS/BACE1 axis in Tat-mediated induction of astrocytic amyloidosis, which could be targeted as adjunctive therapies for HAND-associated Alzheimer-like comorbidity.

## Abstract 2

This study shows that astrocytes contribute to the Alzheimer-like pathology seen in the brains of individuals with HIV, and that this is regulated by the transcription factor HIF-1α and the lncRNA BACE1-AS and can contribute to HIV-associated neurocognitive disorders.

## Introduction

The life span of individuals living with HIV-1 has significantly increased due to effective combination antiretroviral therapy (cART). Paradoxically, however, despite suppressed viremia and increased life spans, there is continued high prevalence of HIV-associated neurocognitive disorder (HAND) [1,2] and other HIV-associated non-AIDS conditions that are closely associated with accelerated or premature aging of individuals infected with HIV [3]. Factors contributing to premature aging include chronic HIV-1 infection, persistent immune activation, and long-term usage of cART, all of which may predispose the individuals infected with HIV to age-related neurodegenerative disorders [4]. The aging HIV population has been shown to have increased vulnerability to age-related complications, which is associated with increased prevalence of HIV-associated neurocognitive impairment [1,2,5]. Chronic HIV infection and aging could thus intersect to impair global cognitive functioning [3]. A key co-pathogenic factor in the long-term survival of patients diagnosed with HIV treated with cART is age. The current thinking underlying the persistence of HAND is thought to involve aging, poor penetration of the antiretroviral drugs across the blood-brain barrier into the central nervous system (CNS), and residual chronic inflammation, resulting in persistent CNS low-level viral replication with accumulation of cytotoxic viral proteins such as HIV-1 transactivator of transcription (Tat) and gp120, as well as the potential emergence of resistant viral species and viral escape [6,7]. Additionally, cART itself has been implicated as a possible contributor to the process of aging and neurodegeneration. Intriguingly, one of the neuropathological findings in HIV-1 individuals is the deposition of amyloid β plaques in the brain, which in turn may contribute to the progression and pathology of HAND [8,9].

Deposition of amyloid plaques and tangles are a major hallmark of Alzheimer disease (AD) [10,11], and their role in neurodegeneration has been an area of intense investigation and interest. While HIV-1 infection and the viral Tat protein have been reported to induce amyloidosis, these reports have primarily focused on neurons and endothelial cells [12,13]. In these cells, HIV-1 Tat has been shown to impact amyloidosis by various mechanisms; e.g., HIV-1 Tat disrupts endolysosomal structure and function of neurons, resulting in accumulation of amyloid precursor protein (APP), Aβ, and its converting enzyme, β-site cleaving enzyme (BACE1), leading to increased cleavage of APP by BACE1, thus increasing the amyloid burden [14]. Tat-induced neuronal amyloidosis was also reported to be mediated by recruitment of APP to the lipid rafts, followed by cleavage with active BACE 1 [15]. Chemokine monocyte chemoattractant protein-1 (MCP-1) has also been implicated in the accumulation of amyloids in the brains in presence of Tat [16]. Not only the production of amyloids but degradation of amyloids has also been shown to be impacted by Tat. For example, Tat has been shown to inhibit neprilysin (Aβ degrading enzyme) activity in postmortem brain samples from patients diagnosed with HIV. More precisely, recombinant Tat (specifically with the cysteine-rich domain), when added directly to brain cultures, resulted in a 125% increase of soluble Aβ, and this was due to inhibition of neprilysin activity [17]. Tat has been also reported to decreased activity of neprilysin, resulting in inhibition of microglial phagocytosis of amyloids and thus adding to the burden of amyloid load in the brain [18]. Furthermore, Tat has also been shown to directly interact with Aβ and to form complexes that are more neurotoxic than Aβ alone [19]. Apart from the production and degradation, transport of amyloids has also been reported to be effected by Tat. In endothelial cells, the presence of Tat is reported to result in decreased expression of lipoprotein receptor-related protein 1 (which transports Aβ from brain to blood), while increasing the expression of the receptor for advanced glycation end products (transports Aβ to the brain), thereby perturbing amyloid transportation and contributing to increased amyloid burden [20]. Increased amyloid deposition has been implicated as one of the contributing factors leading to HAND in individuals infected with HIV with the AD genetic risk factor of apolipoprotein E4 (APOE ε4) [21]. Recent reports have also shown that antiretrovirals (zidovudine, lamivudine, indinavir, and abacavir) alone and in combination can inhibit microglial phagocytosis of amyloids and induce neuronal amyloid production, thus also contributing to amyloid burden [22]. While neurons are the major known producers of amyloid proteins, more recently, glial cells such as the astrocytes have also garnered interest as contributors of amyloidosis [23]. Astrocytes are an abundant glial cell population in the CNS. Their contribution to amyloidosis, if at all, could thus add a significant burden to the process of amyloidosis in response to HIV infection and/or HIV-1 Tat. To date, however, there are no reports on the role of astrocytes in HIV-1 Tat–induced amyloidosis, which in turn could have a significant effect on neurotoxicity associated with HAND.

Sequential cleavage of APP by BACE1 is essential for the generation of the toxic forms of Aβ [24]. Aβ 1–42 oligomers produced by cleavage with BACE1 are toxic and are known to contribute to neurodegeneration leading to behavioral impairments. BACE1 can thus be considered as a key regulator of amyloidopathy. Recent studies have identified critical mediators regulating production of BACE1. Among these transcription factor, such as hypoxia-inducible factor (HIF-1α) [25] and long noncoding RNAs (lncRNAs) [26] have gained considerable attention in recent years. For example, BACE1‐antisense transcript (BACE1-AS) lncRNA has been shown to elevate BACE1 mRNA stability and protein abundance in neurons, leading to increased Aβ production in patients with AD [26]. It has been also observed that HIF-1α can induce the production of BACE 1 in the AD model [27]. The role of HIF-1α and BACE1-AS in HIV-1 Tat–induced amyloidosis, however, remains unexplored.

Herein, we report astrocytic amyloidosis in the archival brain tissue of rhesus macaques chronically infected with simian immunodeficiency virus (SIV) and also in humans infected with HIV, the extent of amyloidosis in the latter correlating with grade of cognitive impairment. Furthermore, dissection of the molecular pathway involved in Tat-mediated increased expression of amyloids in human primary astrocytes (HPAs) involved up-regulated expression of HIF-1α and its subsequent interaction with lncRNA BACE1-AS to form a unique complex. This complex, in turn, was shown to regulate the synthesis and stabilization of BACE 1 via multiple regulatory mechanisms (transcription, posttranscription, translation, and posttranslation), leading to increased expression of the cleaved toxic Aβ 1–42 form. This is the first report of HIV-1 Tat–mediated induction of astrocytic amyloidosis involving the HIF-1α-lncRNA BACE1-AS axis that could also be a potential contributor to the progression of pathogenesis of HAND.

## Results

### Amyloidosis and HAND in patients diagnosed with HIV

**Fig 1:**
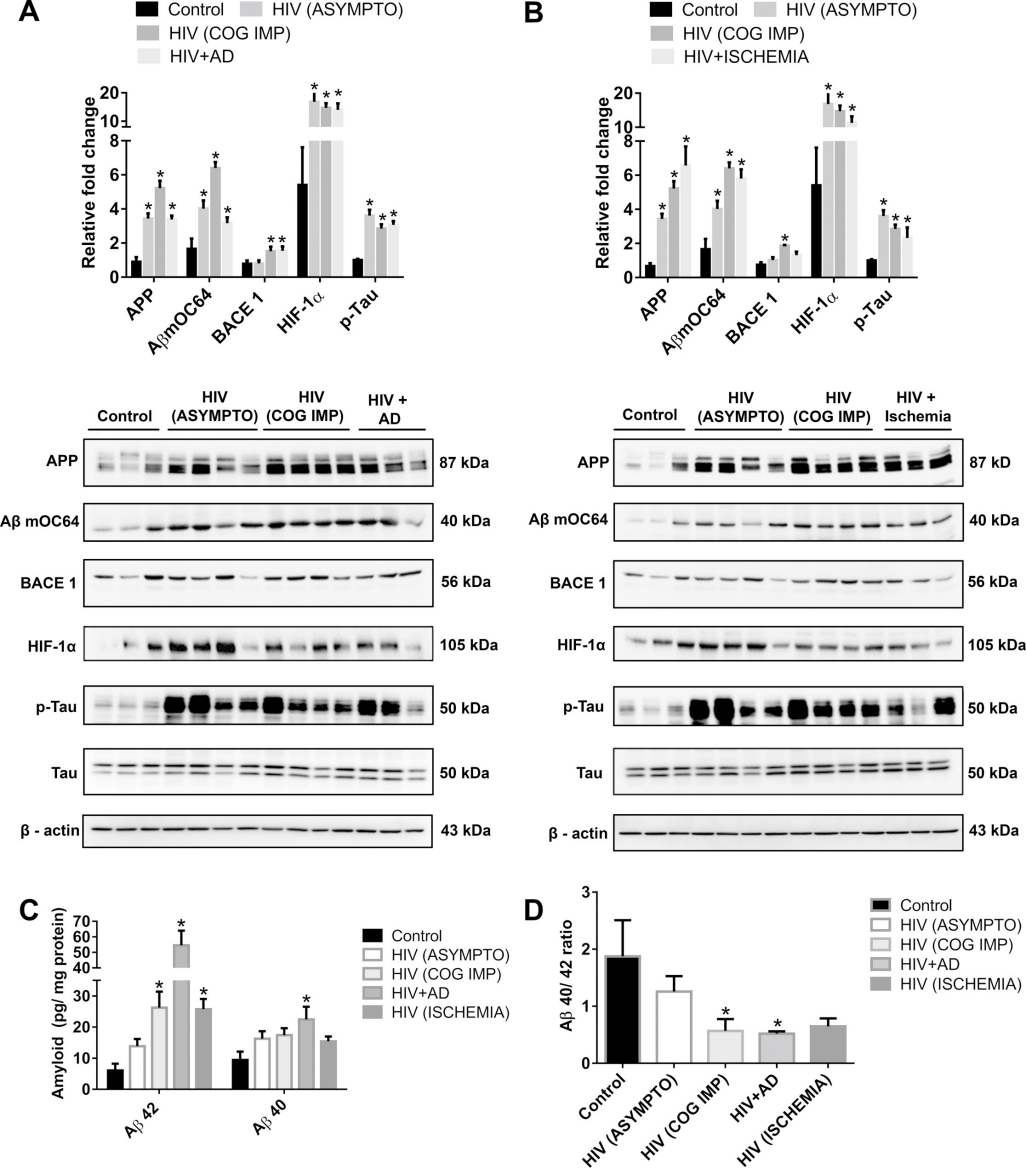
Amyloidopathy and neurofibrillary tangles in the FCs of individuals infected with HIV. (A) Representative western blots showing the expression of APP, Aβ mOC64, BACE1, HIF-1α, p-Tau, and Tau in the FC of control (uninfected, *n* = 3), patients infected with HIV with asymptomatic [HIV (ASYMPTO), *n* = 4], cognitive impairment [HIV (COG IMP), *n* = 4], those with neurofibrillar pathology (HIV + AD, *n* = 3), (B) as well as with cerebral ischemia (HIV + Ischemia, *n* = 3). β-actin was used as an internal control. (C) ELISA showing the protein levels of Aβ42 and 40 and (D) graphical representation of Aβ40/42 ratio. Data are presented as mean ± SEM. One-way ANOVA followed by Bonferroni post hoc test was performed, *P < 0.05 versus control. The data underlying this figure may be found in S1 Data. AD, Alzheimer Disease; APP, amyloid precursor protein; Aβ, amyloid beta; BACE1, β-site cleaving enzyme; FC, frontal cortex; HIF-1α, hypoxia-inducible factor.

We first sought to assess the expression of toxic amyloid proteins and HIF-1α in brain homogenates of chronically infected patients who are HIV positive (archival tissues obtained from NeuroAIDS Tissue Consortium [NNTC]) and were diagnosed with either asymptomatic neurocognitive impairment (neurocognitively impaired without a clear impact on daily activities) or that had minor cognitive impairment (these two groups differ, as the latter have functional impairment [28]). Patients infected with HIV with AD/cerebral ischemia were chosen as positive controls in addition to the uninfected group that served as negative controls. One-way ANOVA followed by Bonferroni post hoc test was employed for determining statistical significance. Our findings demonstrated significantly up-regulated *(*P* < 0.05) expression of the toxic Aβ mOC64 form, APP, HIF-1α, as well as p-Tau in the brains of various groups of patients infected with HIV (asymptomatic as well as minor cognitive impairment, AD, cerebral ischemia) compared to uninfected individuals. Intriguingly, expression of BACE1 protein was significantly up-regulated *(*P* < 0.05) only in the minor cognitive impaired group and in patients diagnosed with HIV with a diagnosis of AD (Fig 1A and 1B), compared to uninfected individuals. Furthermore, validation using ELISA data also showed a significantly higher *(*P* < 0.05) expression of the toxic Aβ42 in the patients who are HIV positive with minor cognitive impairment, AD, and cerebral ischemia groups compared to that of uninfected individuals. Expression of Aβ40, on the other hand, remained unchanged in all of the groups except in patients who are HIV positive with AD (Fig 1C). There was also a significant *(*P* < 0.05) reduction in the ratio of Aβ40/42 in patients who are HIV positive with cognitive impairment and with AD (Fig 1D) compared to uninfected individuals. These results demonstrated deposition of both toxic amyloid proteins and p-Tau in the brains of patients who are HIV positive in different groups of HAND. However, up-regulation of Aβ42 (reduction of the Aβ 40/42 ratio) was only observed in the HIV^+^ cognitive impaired group with functional abnormalities (minor cognitive impairment) but not in the asymptomatic group. Additionally, there was also up-regulation of HIF-1α in the brains of all the HIV^+^ groups compared with the uninfected controls.

### Enhanced expression of Aβ1–42 in patients diagnosed with HIV with HAND

**Fig 2:**
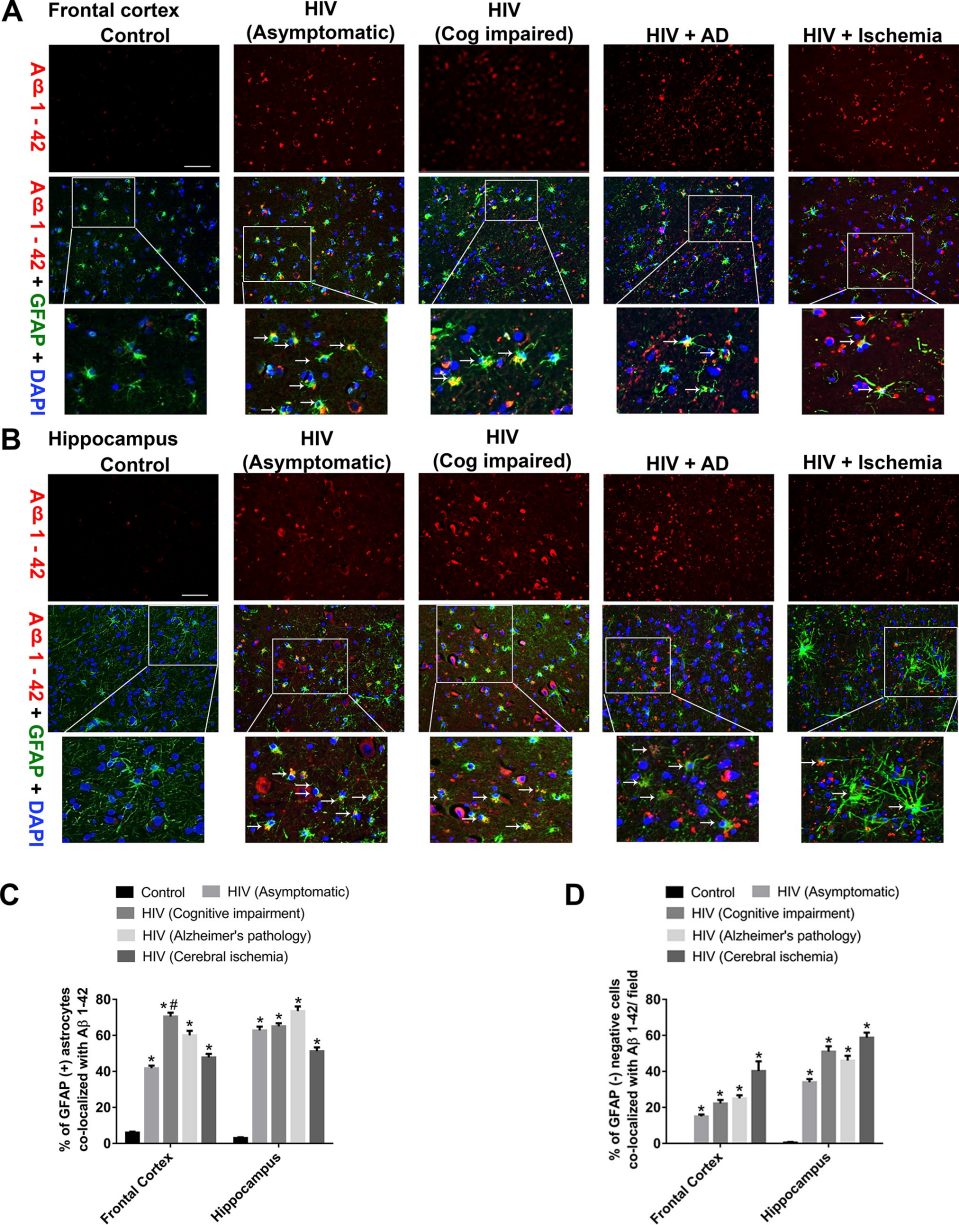
Differential expression of Aβ1–42 in the FC and Hp of patients infected with HIV. Representative fluorescent photomicrographs from different groups exhibiting differential expression of Aβ1–42 in GFAP^+^ astrocytes in the in the FC (A) and H (B), Scale bar, 10 μm. Quantitative analysis of percent of GFAP^+^ astrocytes (C) and GFAP^−^ cells (D) colocalized with Aβ1–42. Ten fields from each brain region/patient were analyzed. One-way ANOVA followed by Bonferroni post hoc test was used to determine the statistical significance: *P < 0.05 versus control, ^#^*P* < 0.05 versus HIV (ASYMPTO), (COG IMP). Data are presented as mean ± SEM. Arrows indicate GFAP-positive astrocytes colocalized with Aβ 1–42. The data underlying this figure may be found in S2 Data. AD, Alzheimer disease; Aβ, amyloid beta; FC, frontal cortex; GFAP, glial fibirillary acidic protein; Hp, hippocampus.

The next step was to assess the presence of Aβ1–42 in glial fibrillary acidic protein (GFAP)-positive astrocytes. For this, brain sections from various HIV+ groups were co-immunostained for the presence of Aβ1–42 and GFAP. One-way ANOVA followed by Bonferroni post hoc test was employed for determining statistical significance among different groups. As shown in Fig 2, there was increased expression of Aβ1–42 in the frontal cortices (FCs) and hippocampi of all HIV+ groups with a significant *(*P* < 0.05) colocalization with GFAP-positive astrocytes. Interestingly, there was significantly increased *(*P* < 0.05) colocalization of GFAP-positive astrocytes with Aβ1–42 in the HIV+ cognitively impaired group compared with the HIV+ asymptomatic group in the FC. This differential effect, however, was not obvious in the hippocampus (Hp), where both the asymptomatic as well as the cognitively impaired groups exhibited comparable expression of Aβ1–42 in the GFAP^+^ astrocytes (Fig 2A, 2B and 2C). Additionally, we have also observed that GFAP-negative cells also showed increased expression of Aβ1–42, indicating that cells other than astrocytes also produce amyloids. Quantification data showed that the percent of amyloids in GFAP negative cells are between 20% and 45% and that for positive cells are between 45% and 70% (Fig 2C and 2D). While quantifying, we found that several amyloids did not colocalize with the DAPI-nucleus, indicating extracellular amyloid deposition. The GFAP-negative cells are not necessarily neurons, as endothelial cells are also producers of amyloid in patients infected with HIV [29].

### Brain region-specific amyloidosis in SIV-infected macaques

**Fig 3:**
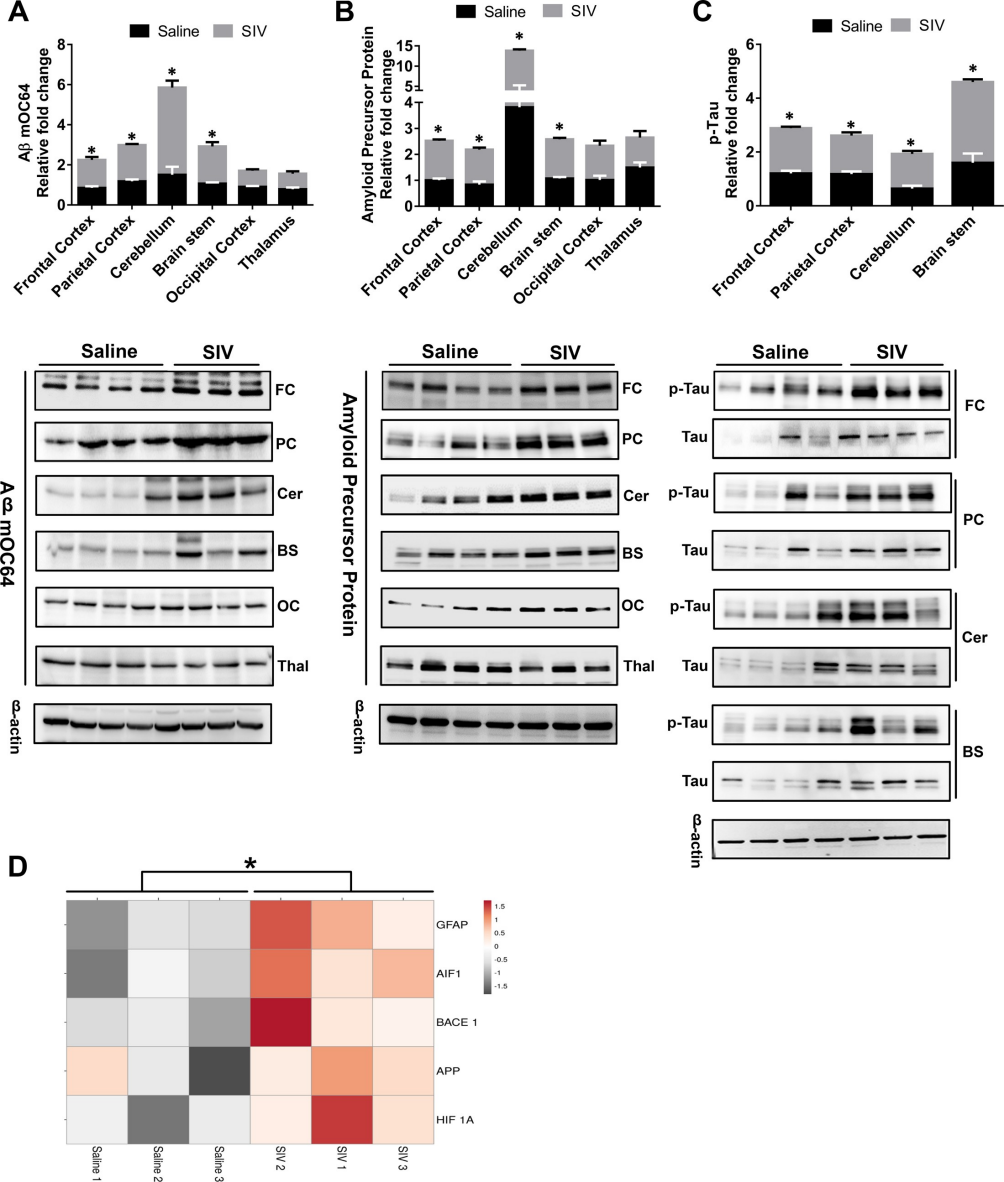
Amyloidopathy and neurofibrillary pathology in the brain regions of SIV-infected macaques. Representative western blots showing the expression of Aβ mOC64 (A) and APP (B) and p-Tau/Tau (C) in different brain regions—FC, PC, Cer, BS, OC, Thal—of saline and SIV^+^ macaques. (D) RNA-seq analysis showing expression of HIF 1A, APP, BACE1, GFAP, and AIF1 in the FC of saline- and SIV-infected macaques. Data are presented as mean ± SEM; *n* = 6. Student *t* test was used to determine the statistical significance: *P < 0.05 versus saline. The data underlying this figure may be found in S3 Data. AIF1, allograft inflammatory factor 1; APP, amyloid precursor protein; Aβ, amyloid beta; BACE1, β-site cleaving enzyme; BS, brain stem; Cer, cerebellum; FC, frontal cortex; GFAP, glial fibrillary acidic protein; OC, occipital cortex; PC, parietal cortex; RNA-Seq, RNA-sequencing; SIV, simian immunodeficiency virus; Thal, thalamus.

We next sought to validate our findings using the archival brain tissues of chronically SIV-infected macaques. We assessed the expression of Aβ mOC64, APP, and p-Tau in a number of brain regions (FC, parietal cortex [PC], cerebellum [Cer], and brain stem [BS]) of chronically SIV-infected macaques and the uninfected animals. Multiple *t* test was employed to determine the statistical significance between saline- and SIV-infected macaques. As shown in Fig 3A, 3B and 3C, there was significant up-regulation *(*P* < 0.05) of toxic Aβ mOC64, APP, and p-Tau protein in the specific brain regions FC, PC, Cer, and BS of the SIV-infected macaques versus the uninfected group. We also assessed expression of these proteins in other brains regions such as the occipital cortex (OC) and thalamus (Thal) but failed to see any differences in these regions within the two groups of macaques (Fig 3A, 3B and 3C). These results underscored brain region–specific deposition of toxic amyloid and p-Tau proteins in the SIV-infected animals with a predilection of FC, PC, Cer, and BS regions for the deposition of these toxic proteins. We next performed RNA sequencing (RNA-seq) analysis of the FC region from both groups of macaques (SIV^+^ and uninfected) and observed significant up-regulation *(*P* < 0.05) of APP, HIF1A, BACE1, and GFAP RNA in SIV^+^ versus the saline administered group (Fig 3D).

Table 1 demonstrates the viral load and the CD4/CD8 counts of the SIV-infected macaques 12 months postinfection (on the day of necropsy).

**Table 1:** CSF viral loads, CD4 and CD8 counts in SIV-infected macaques.

Monkey ID	Viral load (CSF)	CD4 count	CD8 count
**C1**	6,574	249	331
**C2**	633,917	132	165
**C4**	49,212	172	275

Abbreviation: CSF, cerebrospinal fluid

### In situ hybridization of APP in the brains of SIV-infected monkeys and patients infected with HIV

**Fig 4:**
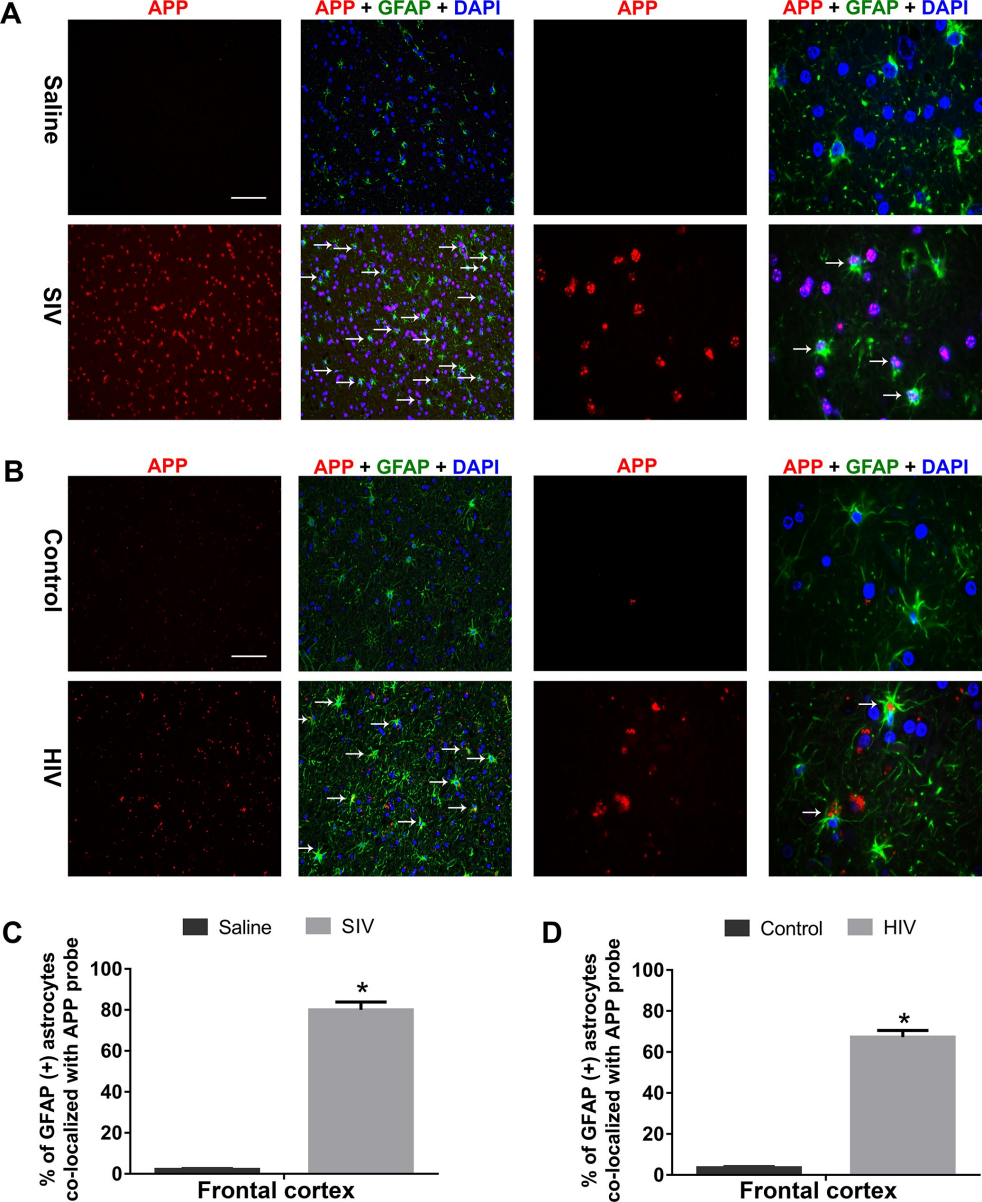
Expression of APP RNA by in situ hybridization in the FCs of SIV-infectedmacaques and patients who are infected with HIV. Representative FISH and IF photomicrographs showing differential expression of APP RNA in GFAP^+^ astrocytes in SIV^+^ macaques (A) and patients who are HIV positive (B). Scale bar, 10 μm. Quantitative analysis of percent GFAP^+^ astrocytes colocalized with APP RNA in saline and SIV^+^ (C) and patients who are HIV positive (D). Ten fields from FC/macaque/patient were analyzed from *n* = 3 macaques and *n* = 3 patients. Data are presented as mean ± SEM. Student *t* test was used to determine the statistical significance: *P < 0.05 versus saline/control. Arrows indicate GFAP-positive astrocytes colocalized with APP RNA. The data underlying this figure may be found in S4 Data. APP, amyloid precursor protein; FC, frontal cortex; GFAP, glial fibrillary acidic protein; SIV, simian immunodeficiency virus.

To further confirm the process of astrocytic amyloidosis ex vivo, we next performed in situ hybridization RNA fluorescent in situ hybridization (RNA FISH) using the APP probes to detect expression of APP RNA in the FC of SIV-infected monkeys and patients infected with HIV. Multiple *t* test was employed to determine the statistical significance between saline/control and SIV-infected monkeys and patients infected with HIV. As shown in Fig 4A, 4B, 4C and 4D, APP RNA was significantly up-regulated *(*P* < 0.05) in the GFAP^+^ astrocytes present in the FC of both SIV- and HIV-infected groups, while only a mild expression of APP RNA was observed in the FC of uninfected macaques/humans. Fig 4C and 4D are the quantification of the immunohistochemistry of Fig 4A and 4B.

### HIV-1 Tat induces amyloidosis in HPAs

**Fig 5:**
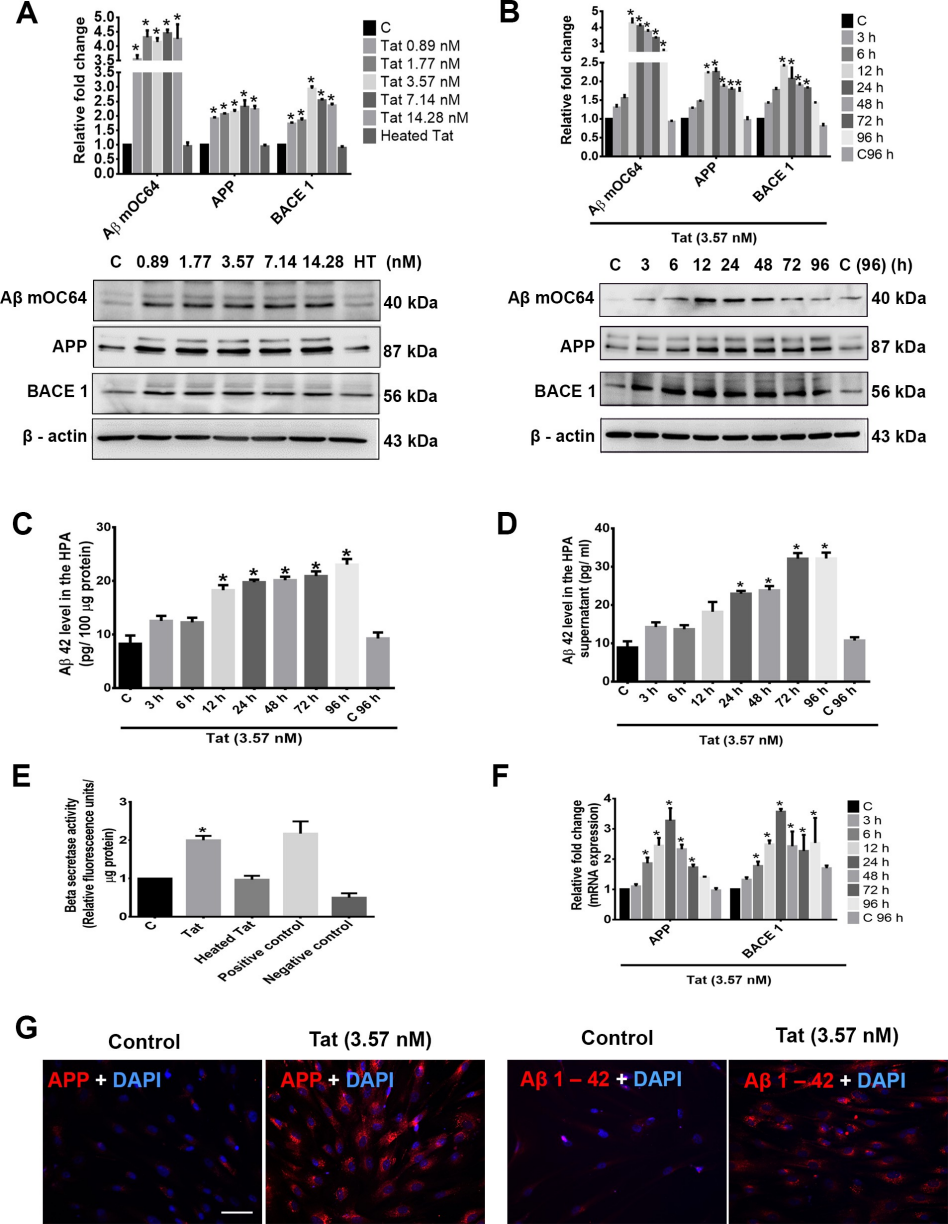
HIV-1 Tat–mediated amyloidosis in HPAs. Western blot analysis showing dose- (A) and time-dependent (B) up-regulation of Aβ mOC64, APP, and BACE1 in HPAs exposed to HIV-1 Tat at the indicated doses and time points. β-actin was used as an internal control. ELISA assay showing time-dependent up-regulation of Aβ42 protein in HPA lysates (C) as well as in the supernatant fluids (D) following exposure of cells to HIV-1 Tat (3.57 nM). (E) HIV-1 Tat (3.57 nM)–exposed HPAs showed increased β-secretase activity by spectrofluorometric analysis. (F) qPCR analysis showing expression of APP and BACE1 mRNA in HPAs exposed to HIV-1 Tat (3.57 nM) at the indicated time points. GAPDH was used as an internal control for mRNA expression. (G) Representative fluorescent photomicrographs showing increased expression of APP and Aβ1–42 proteins in HPAs exposed to HIV-1 Tat (3.57 nM) for 24 hours. Scale bar, 10 μm. Data are presented as mean ± SEM; *n* = 6. One-way ANOVA followed by Bonferroni post hoc test and Student *t* test was used to determine the statistical significance: *P < 0.05 versus control. The data underlying this figure may be found in S5 Data. APP, amyloid precursor protein; Aβ, amyloid beta; BACE1, β-site cleaving enzyme; GAPDH, glyceraldehyde 3-phosphate dehydrogenase; HPA, human primary astrocyte; qPCR, quantative polymerase chain reaction; Tat, transactivator of Tat.

Based on our ex vivo findings, we next sought to explore whether we could simulate the effect of HIV/SIV-mediated amyloidosis in HPAs exposed to the viral protein Tat. One-way ANOVA followed by Bonferroni post hoc test was employed for determining statistical significance among different groups. HPAs were exposed to varying concentrations of HIV-1 Tat (0.89, 1.77, 3.57, 7.14, 14.28 nM) for 24 hours followed by assessment of cell lysates for the expression of APP, HIF-1α, BACE1, BACE1-AS, Aβ mOC64, and Aβ 42 in the supernatant. As shown in Fig 5A, HIV-1 Tat significantly *(*P* < 0.05) increased the expression of conformation-specific Aβ mOC64, APP, as well as BACE1 compared to that of control. The serum and cerebrospinal fluid (CSF) concentrations of HIV-1 Tat in patients diagnosed with HIV was reported to range from 0.07–2.85 nM [30,31], and an even higher concentration of Tat than this has been suggested to be present in the vicinity of HIV positive perivascular cells in the CNS [32]. Because 3.57 nM Tat showed consistent and significant up-regulation of all the markers and closely matches with the reported amounts of Tat in the CNS, this concentration was chosen for future experimentation. The next step was to determine the time course for the expression of these markers. As shown in Fig 5B, HIV-1 Tat (3.57 nM) significantly *(*P* < 0.05) increased the expression levels of Aβ mOC64, APP, as well as BACE1 in a time-dependent manner (0, 3, 6, 12, 24, 48, 72, and 96 hours) in HPAs, with consistent up-regulation at 24 hours post Tat treatment compared to control. Based on the dose- and time-course studies, the optimal dose and time for Tat effect was 3.57 nM for 24 hours and was the chosen dose and time for all subsequent experiments. HPAs exposed to Tat also demonstrated significant *(*P* < 0.05) up-regulation of the Aβ 42 protein both intracellularly as well as in the supernatant fluids (Fig 5C and 5D). HIV-1 Tat also significantly up-regulated *(*P* < 0.05) BACE1 activity in HPAs (Fig 5E). Further validation of Tat-mediated induction of astrocytic amyloidosis was also done by assessing the mRNA expression of APP and BACE1 in HPAs exposed to Tat. As shown in Fig 5F, there was significant *(*P* < 0.05) time-dependent up-regulation of both APP and BACE1 in HPAs exposed to Tat compared to control. These findings were also validated by immunostaining in Tat-exposed HPAs for APP and Aβ1–42. As shown in Fig 5G, there was increased expression of APP and Aβ1–42 in HPAs exposed to Tat.

### HIV-1 Tat induces HIF-1α and lncRNA BACE1-AS expression in HPAs

**Fig 6:**
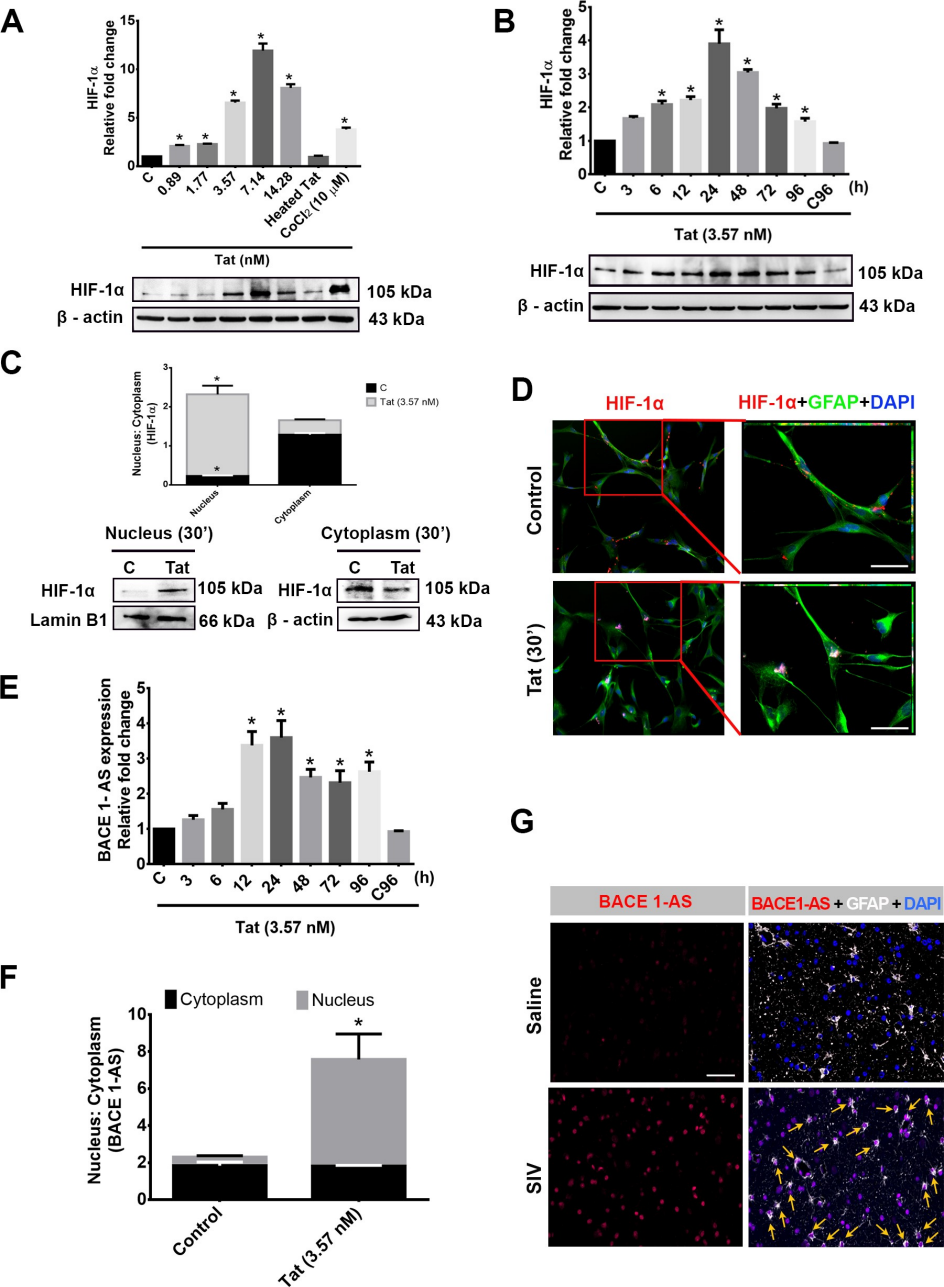
HIV-1 Tat–mediated expression of HIF-1α and BACE1-AS in HPA. Western blot analysis showing dose- (A) and time-dependent (B) up-regulation of HIF-1α in HPAs exposed to HIV-1 Tat at the indicated dose and time points. Cobalt chloride (CoCl_2_−10 μM) was used as a positive control for HIF-1α expression. (C) Nuclear and cytoplasmic expression of HIF-1α protein 30 minutes following exposure of cells to HIV-1 Tat (3.57 nM). (D) Representative fluorescent photomicrographs showing increased expression of HIF-1α after 30 minutes of HIV-1 Tat (3.57 nM) exposure in HPAs. (E) qPCR analysis demonstrating expression of lncRNA BACE1-AS in HPAs exposed to HIV-1 Tat (3.57 nM) at the indicated time points. (F) Nuclear and cytoplasmic expression of BACE1-AS RNA following exposure to HPAs to HIV-1 Tat (3.57 nM) for 12 hours. (G) Representative FISH and immunofluorescence photomicrographs demonstrating increased expression of BACE1-AS RNA in the GFAP^+^ astrocytes in the FC of SIV^+^ macaques compared with the saline group. Scale bar, 10 μm. Data are presented as mean ± SEM; *n* = 6. One-way ANOVA followed by Bonferroni post hoc and Student *t* test was employed to determine the statistical significance: * *P* < 0.05 versus control. Arrows indicate GFAP-positive astrocytes colocalized with BACE1-AS RNA. The data underlying this figure may be found in S6 Data. BACE1-AS, BACE1‐antisense transcript; FC, frontal cortex; FISH, fluorescence insitu hybridization; GFAP, glial fibrillary acidic protein; HIF-1α, hypoxia-inducible factor; HPA, human primary astrocyte; lncRNA, long noncoding RNA; qPCR, quantative polymerase chain reaction; SIV, simian immunodeficiency virus; Tat, transactivator of transcription.

Having determined Tat-mediated induction of APP and Aβ1–42 in HPAs, the next step was to assess the molecular mechanisms involved in this process. Both HIF-1α [27] and lncRNA BACE1-AS [26] are known inducers of BACE1 enzyme, which is crucial for sequential cleavage of Aβ from APP [33]. We thus next sought to determine the expression of both of these inducers in HPAs exposed to Tat. As shown in Fig 6A and 6B, HIV-1 Tat significantly *(*P* < 0.05) up-regulated the expression of HIF-1α in a both dose- and time-dependent manner compared to that of control. HIV-1 Tat also significantly *(*P* < 0.05) (as determined by *t* test) enhanced the translocation of HIF-1α from the cytoplasm to nucleus within 30 minutes of exposure (Fig 6C). Nuclear translocation of HIF-1α was also confirmed by immunofluorescence analysis (Fig 6D).

Similar to the induction of HIF-1α, Tat also significantly up-regulated *(*P* < 0.05) (as evident from ANOVA) the expression of lncRNA BACE1-AS in a time-dependent manner (Fig 6E), with significantly increased expression *(*P* < 0.05) (as shown by *t* test) in the nuclear fraction compared to that in the cytoplasm (Fig 6F). Tat-mediated up-regulation of BACE1-AS was also validated by in situ hybridization in the FCs of SIV-infected macaques. As shown in Fig 6G, in situ hybridization of BACE1-AS demonstrated an increased expression in the nucleus of GFAP^+^ astrocytes of SIV-infected macaques compared to the saline group. It should be noted that besides astrocytes, increased expression of BACE1-AS was also observed in other CNS cells.

### HIF-1α regulates the expression of lncRNA BACE1-AS in HPAs

**Fig 7:**
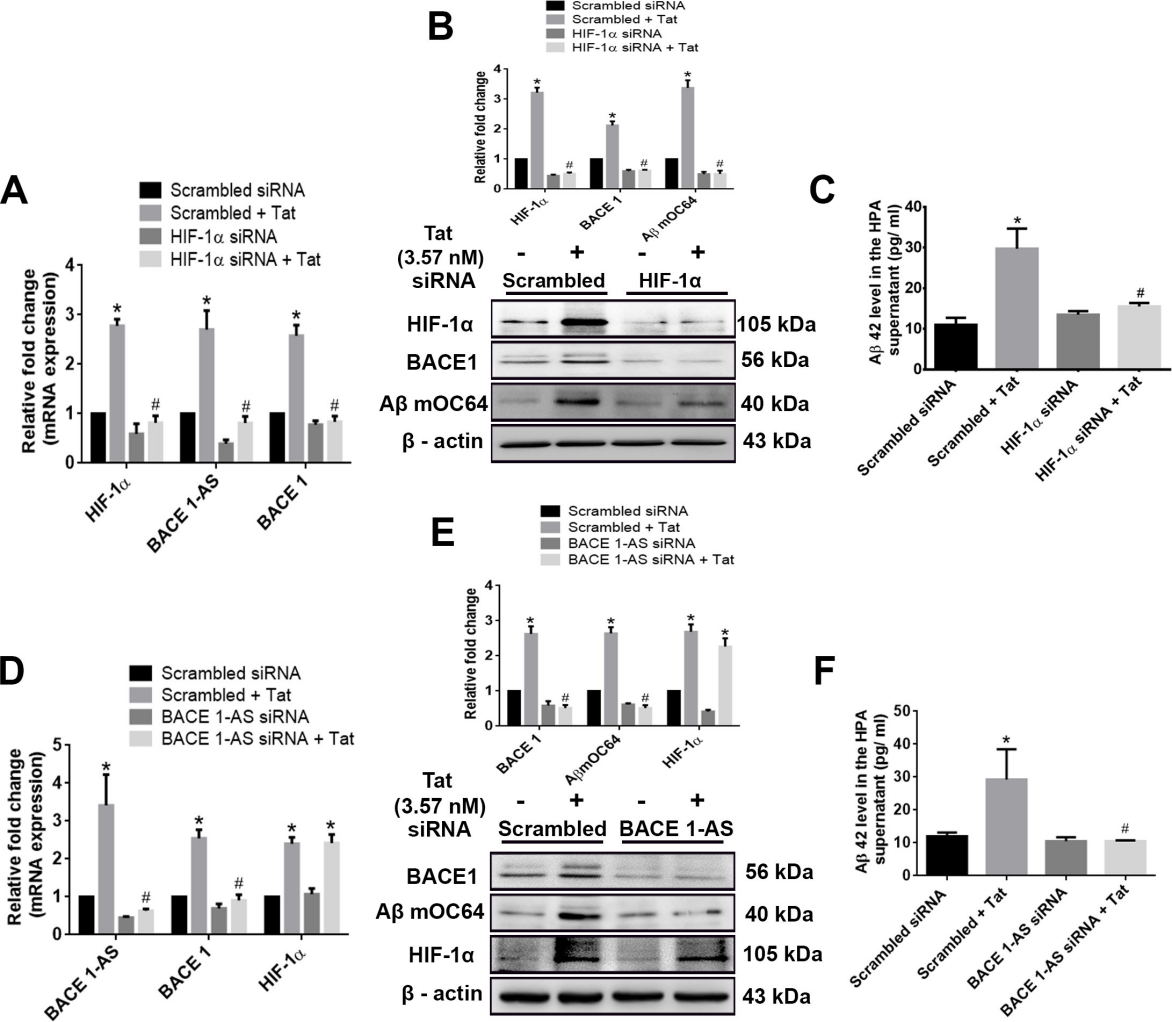
HIV-1 Tat–mediated regulation of HIF-1α, BACE1-AS, and BACE1 in modulating astrocytic amyloidosis. (A) qPCR analysis demonstrating expression of HIF-1α, BACE1-AS, and BACE1 mRNA in HPAs transfected with either HIF-1α or scrambled siRNA in the presence or absence of HIV-1 Tat (3.57 nM; 24 hours). (B) Representative western blots showing the expression of HIF-1α, BACE1, and Aβ mOC64 proteins in HPAs transfected with either HIF-1α or scrambled siRNA. (C) ELISA showing the protein levels of Aβ 42 in the supernatant fluids of HPAs transfected with either HIF-1α or scrambled siRNA. (D) qPCR demonstrating expression of BACE1-AS, BACE1, and HIF-1α RNA in HPAs transfected with either BACE1-AS or scrambled siRNA. (E) Representative western blots showing the expressions of BACE1, Aβ mOC64, and HIF-1α in HPA transfected with either BACE1-AS or scrambled siRNA. (F) ELISA showing the protein levels of Aβ42 in the supernatant of HPA transfected with either BACE1-AS or scrambled siRNA. Data are presented as mean ± SEM; *n* = 6. One-way ANOVA followed by Bonferroni post hoc test was used to determine the statistical significance: *P < 0.05 versus control, ^#^*P* < 0.05 versus Tat. The data underlying this figure may be found in S7 Data. Aβ, amyloid beta; BACE1, β-site cleaving enzyme; BACE1-AS, BACE1‐antisense transcript; HIF-1α, hypoxia-inducible factor; HPA, human primary astrocyte; qPCR, quantative polymerase chain reaction; siRNA, small interfering RNA; Tat, transactivator of transcription.

Having determined that HIV-1 Tat induced the expression of both HIF-1α and lncRNA BACE1-AS in HPAs, we next sought to determine whether the induction of HIF-1α had an impact on the expression of lncRNA BACE1-AS in these cells using the gene silencing approach. One-way ANOVA followed by Bonferroni post hoc test was employed for determining statistical significance among different groups. HPAs were transfected with either *HIF-1α* small interfering RNA (siRNA) or scrambled siRNA followed by exposure of cells to Tat (3.57 nM) and assessed for the expression of BACE1-AS, BACE1 RNA, and protein, as well as for the expression of Aβ mOC64 and Aβ 42 proteins. Scrambled siRNA-transfected HPAs followed by Tat exposure resulted in significant increase *(*P* < 0.05) in the mRNA expression of HIF-1, BACE1, BACE1-AS, protein levels of HIF-1α, BACE1, AβmOC64, as well as secreted Aβ 1–42 in the supernatant compared to that without treatment. HPAs transfected with HIF-1α siRNA with/without Tat exposure resulted in significant decrease #(*P* < 0.05) in the expression of BACE1-AS and BACE1 RNA as well as BACE1, Aβ mOC64, and the secreted Aβ42 proteins (Fig 7A, 7B and 7C) compared to Tat-exposed HPAs with scrambled siRNA. To further validate these findings, we next silenced the BACE1-AS gene by transfecting the cells with BACE1-AS siRNA, followed by exposure of cells to Tat and assessing cell lysates for the expression of BACE1-AS, BACE1 RNA, and protein, as well as Aβ mOC64 and Aβ 42 proteins. As shown in Fig 7D, 7E and 7F, in BACE1-AS siRNA-transfected HPAs with/without exposure to Tat, the result was a significant decrease #(*P* < 0.05) in the expression of BACE1 RNA and protein, and Aβ mOC64 and Aβ42 was observed, compared to Tat-exposed HPAs with scrambled siRNA. Expression of HIF1-α in siRNA-transfected cells, however, remained unchanged in the presence of Tat.

**Fig 8:**
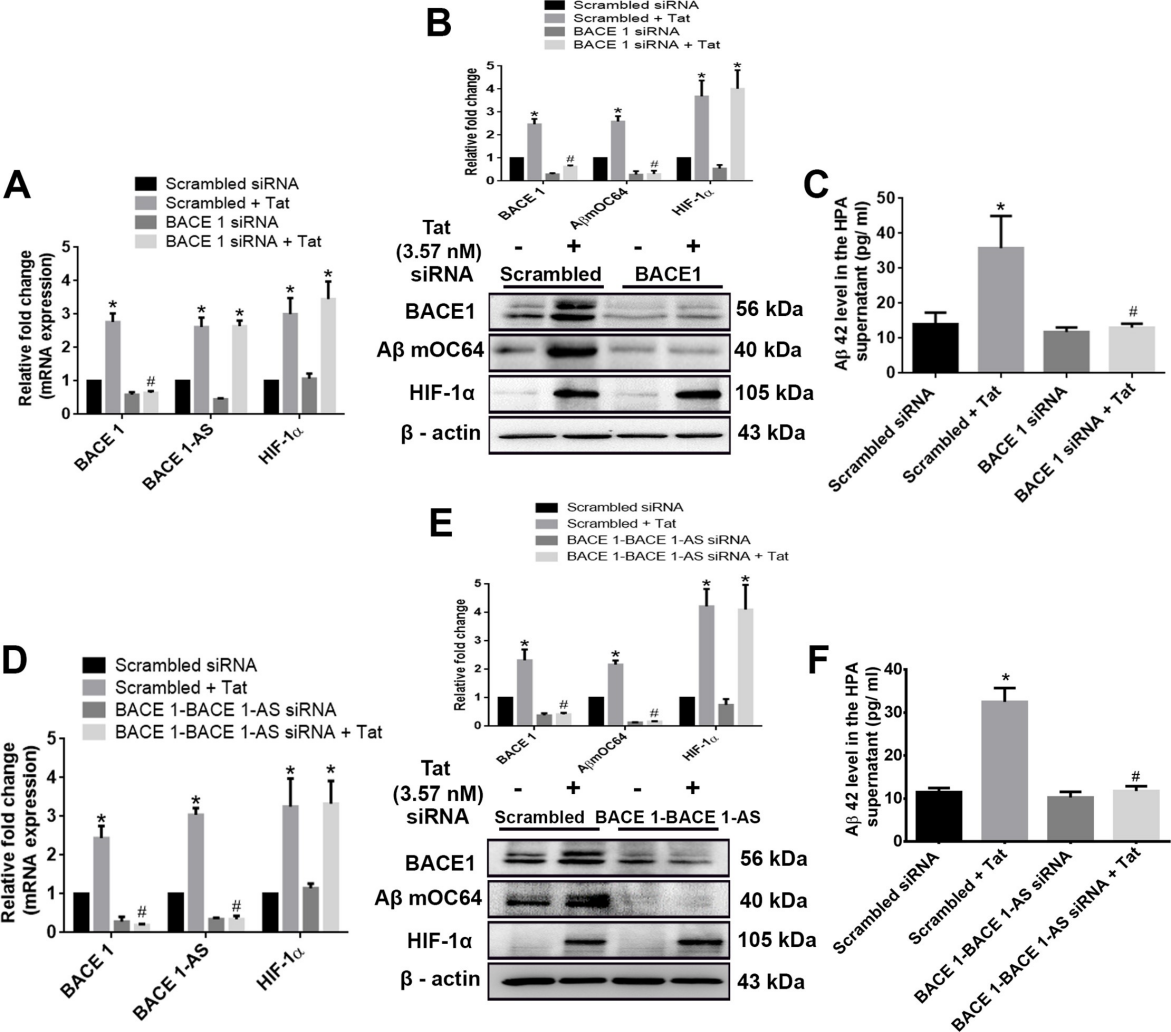
Role of BACE1 and/or BACE1-AS in HIV-1 Tat–mediated astrocytic amyloidosis. (A) qPCR showing expression of BACE1, BACE1-AS, and HIF-1α RNA in HPAs transfected with either BACE1 or scrambled siRNA. (B) Representative western blots showing the expressions of BACE1, Aβ mOC64, and HIF-1α in HPA transfected with either BACE1 or scrambled siRNA. (C) ELISA showing the protein levels of Aβ 42 in the supernatant of HPA transfected with either BACE1 or scrambled siRNA. (D) qPCR showing mRNA expressions of BACE1, BACE1-AS, and HIF-1α in HPA transfected with either BACE1–BACE1-AS or scrambled siRNA. (E) Representative western blots showing the expressions of BACE1, Aβ mOC64, and HIF-1α in HPA transfected with either BACE1–BACE1-AS or scrambled siRNA. (F) ELISA showing the protein levels of Aβ42 in the supernatant of HPA transfected with either BACE1–BACE1-AS or scrambled siRNA. Data are presented as mean ± SEM; *n* = 6. One-way ANOVA followed by Bonferroni post hoc test was used to determine the statistical significance: *P < 0.05 versus control, ^#^*P* < 0.05 versus Tat. The data underlying this figure may be found in S8 Data. Aβ, amyloid beta; BACE1, β-site cleaving enzyme; BACE1-AS, BACE1‐antisense transcript; HIF-1α, hypoxia-inducible factor; HPA, human primary astrocyte; qPCR, quantative polymerase chain reaction; siRNA, small interfering RNA; Tat, transactivator of transcription.

To further ascertain the pathway of Tat-mediated induction of amyloidosis, we next silenced BACE1 in HPAs followed by exposure of the cells to Tat. As shown in Fig 8A, 8B and 8C, in BACE1, siRNA-transfected astrocytes with/without Tat exposure resulted in significant decrease #(*P* < 0.05) in the expression of Aβ mOC64 and secreted Aβ42 compared to Tat-exposed HPAs with scrambled siRNA, while the expression of HIF-1α and BACE1-AS remained unchanged, similar to that in the scrambled siRNA group.

Because BACE1-AS has been shown to bind with the complementary region on the BACE1 gene to induce its transcription [26], we next sought to confirm the phenomenon of concordant regulation of BACE1 with BACE1-AS. For this, we silenced the overlapping binding region of BACE1 with BACE1-AS using siRNA specific for the binding sequence. In cells transfected with the siRNA for the overlapping binding sequence of BACE1/ BACE1-AS with/without Tat exposure, this resulted in significant decrease #(*P* < 0.05) in the expression of BACE1-AS, BACE1, Aβ mOC64 AS, as well as secreted Aβ42 compared to Tat-exposed HPAs with scrambled siRNA. Expression of HIF-1α in the transfected cells, however, remained unchanged in the presence of Tat (Fig 8D, 8E and 8F).

### The HIF-1α/lncRNA BACE1-AS complex binds with the BACE1 promoter in HPAs

**Fig 9:**
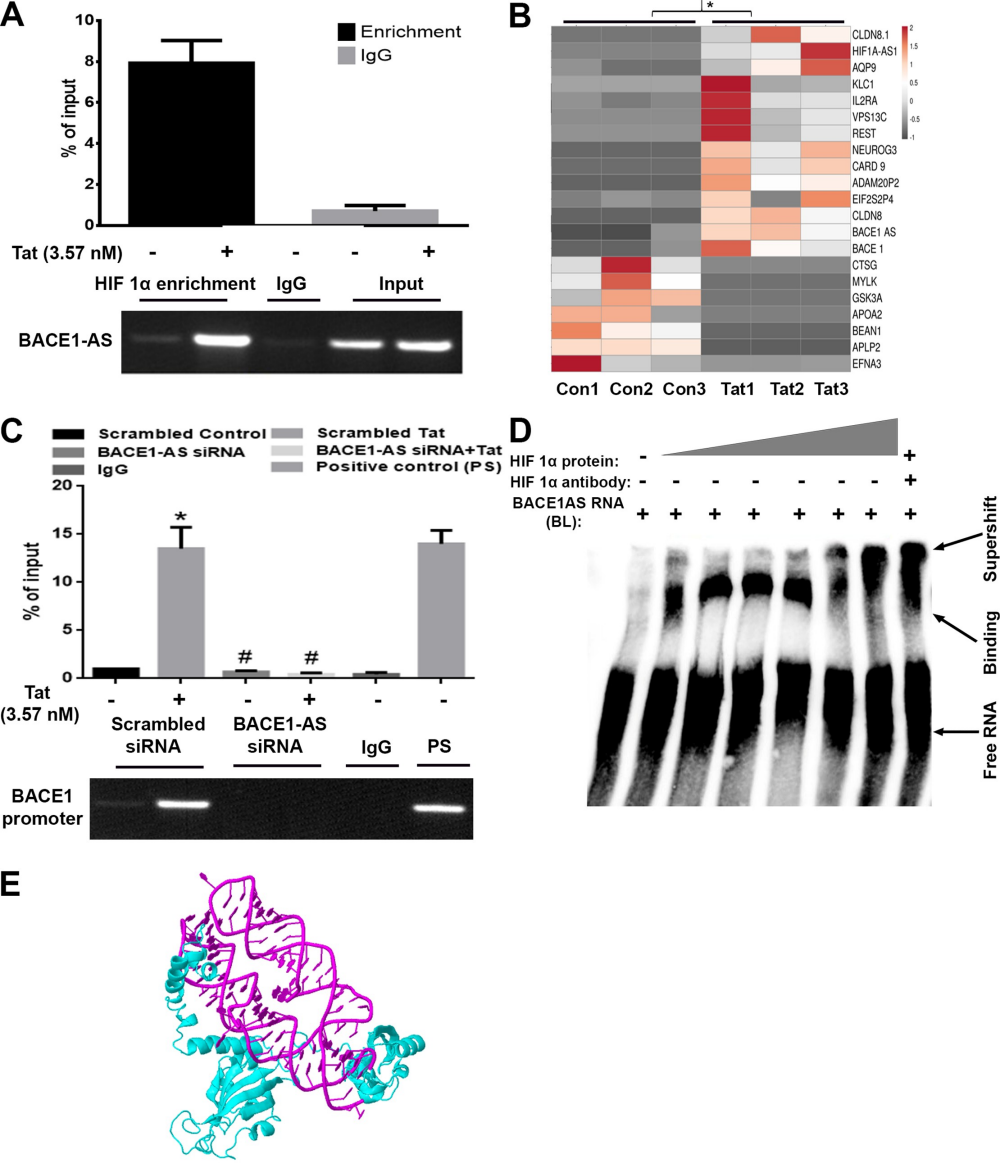
HIF-1α and BACE1-AS complex regulates the transcription of BACE1 in HPAs. (A) RIP assay demonstrating enrichment of BACE1-AS RNA (qPCR) in HIF-1α bound RNA complexes. (B) RNA-seq data from HIF-1α pulled down RNA-enriched product from RIP assay. (C) ChIP assay showing enrichment of BACE1 promoter in the HIF-1α–bound DNA complexes. ChIP was also done in the presence of BACE1-AS siRNA-transfected cells. PCR products at the end of ChIP assay were subjected to agarose gel electrophoresis, demonstrating enrichment of the BACE1 promoter. (D) Representative EMSA image showing dose-dependent binding of HIF-1α to BACE1-AS RNA. (E) Simulated structure of HIF-1α binding to lncRNA BACE-1AS. Data are presented as mean ± SEM; *n* = 6. Student *t* test/one-way ANOVA was employed to determine the statistical significance between two groups: *P < 0.05 versus control. The data underlying this figure may be found in S9 Data. BACE1, β-site cleaving enzyme; BACE1-AS, BACE1‐antisense transcript; BL, biotin-labeled; ChIP, chromatin immunoprecipitation; EMSA, electrophorectic mobility shift assay; HIF-1α, hypoxia-inducible factor; HPA, human primary astrocyte; IgG, immunoglobulin G; lncRNA, long noncoding RNA; PS, positive control; qPCR, quantative polymerase chain reaction; RIP, RNA immunoprecipitation; RNA-seq, RNA-sequencing; siRNA, small interfering RNA.

Based on the fact that the regulation of BACE1-AS by HIF-1α has not been reported thus far, we next sought to assess the nature of interaction of the two using the RNA immunoprecipitation (RIP) assay. In the presence of Tat, the RIP assay showed significant up-regulation *(*P* < 0.05) of the BACE1-AS in the HIF-1α–enriched fraction (HIF-1α protein–bound enriched RNA) (Fig 9A) compared to the untreated group (as determined by *t* test), and this was further confirmed by RNA array of this enriched component, which also showed significant up-regulation *(*P* < 0.05) of BACE1-AS in the Tat-exposed HIF-1α–enriched component compared to that without Tat exposure (Fig 9B). The next step was to perform the chromatin immunoprecipitation (ChIP) assay to assess the binding of the HIF-1α/BACE1-AS complex to the BACE1 promoter. To assess the role of BACE1-AS binding to HIF-1a in mediating transcription of BACE1, HPAs were transfected with either scrambled or BACE1-AS siRNAs followed by exposure of cells to Tat and pulldown of the HIF-1α–bound DNA fraction by ChIP assay. As shown in Fig 9C, in the scrambled siRNA-transfected cells, there was significant enrichment *(*P* < 0.05) of the BACE1 promoter in the HIF-1α protein–bound DNA fraction in the presence of Tat, as also evidenced by the presence of BACE 1 promoter PCR product in the agarose gel compared to that of the untreated group. While in BACE1-AS siRNA-transfected cells exposed to Tat, HIF-1α was unable to bind to the BACE1 promoter, i.e., BACE1-AS siRNA-transfected cells with/without Tat exposure, which resulted in significant decrease #(*P* < 0.05) in the binding of HIF-1α to the BACE1 promoter (as evident by one-way ANOVA, followed by Bonferroni post hoc test). Further confirmation of the binding of HIF-1α to BACE1-AS was also carried out using the electrophorectic mobility shift assay (EMSA). EMSA findings demonstrated that purified HIF-1α protein bound to the in vitro transcribed BACE1-AS RNA in a dose-dependent manner. Furthermore, in the presence of HIF-1α–neutralizing antibody, the HIF1α/BACE1-AS binding complex showed a supershift (Fig 9D). Additionally, we generated a simulated structure using the website http://biophy.hust.edu.cn/3dRPC/result/207188473 to further evaluate the specific interaction(s) of HIF-1α with BACE1-AS, demonstrating several interactions between HIF-1α protein (blue) and BACE1-AS RNA (pink) (Fig 9E).

### Differential regulation of HIV-1 Tat–mediated induction of HIF-1α expression

**Fig 10:**
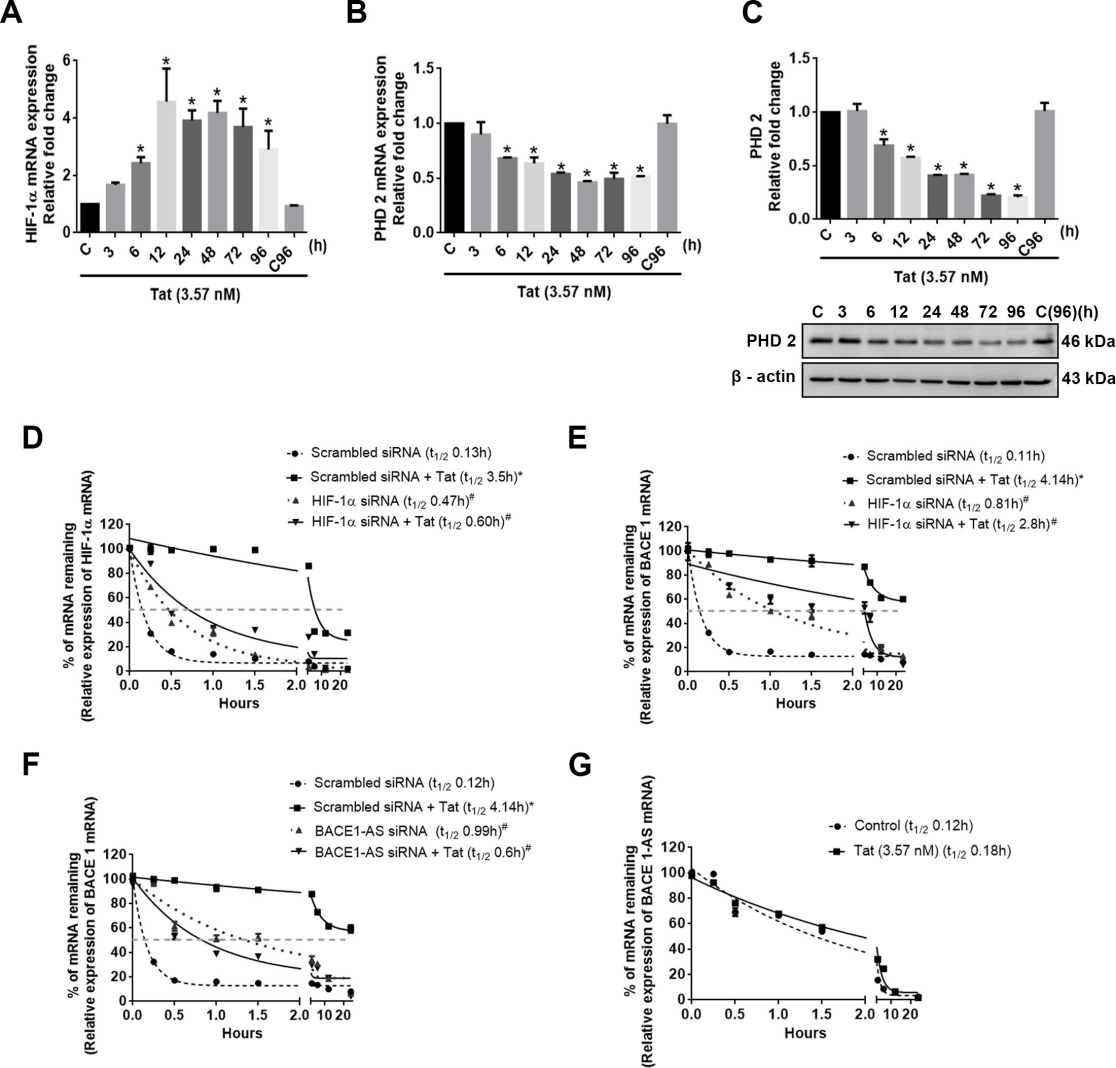
Molecular mechanism(s) involved in HIV-1 Tat–mediated up-regulation of HIF-1α in HPAs. (A) qPCR showing expression of HIF-1α mRNA in HPAs exposed to HIV-1 Tat (3.57 nM) at the indicated time points. (B) qPCR showing expression of PHD-2 in HPAs exposed to HIV-1 Tat (3.57 nM) at the indicated time points. GAPDH was used as an internal control for mRNA expression. (C) Representative western blot showing time-dependent down-regulation of PHD-2 in HPAs exposed to HIV-1 Tat at the indicated time points. β-actin was used as an internal control. (D) Regression analysis showing stabilization of HIF-1α mRNA in HPAs transfected with either scrambled or HIF-1α siRNA in the presence or absence of HIV-1 Tat. (E) Regression lines showing mRNA stabilization and indicating the half-lives of BACE1 in HPA transfected with scrambled/HIF-1α siRNA, with/without HIV-1 Tat exposure. (F) Regression lines showing mRNA stabilization and indicating the half-lives of BACE1 in HPA transfected with scrambled/BACE1-AS siRNA, with/without HIV-1 Tat exposure. (G) Regression lines showing mRNA stabilization and indicating the half-lives of BACE1-AS in HPA with/without HIV-1 Tat exposure. Data are presented as mean ± SEM; *n* = 6. One-way ANOVA followed by Bonferroni post hoc test was used to determine the statistical significance between multiple groups: *P < 0.05 versus control, ^#^*P* < 0.05 versus Tat. The data underlying this figure may be found in S10 Data. BACE1, β-site cleaving enzyme; BACE1-AS, BACE1‐antisense transcript; GAPDH, glyceraldehyde 3-phosphate dehydrogenase; HIF-1α, hypoxia-inducible factor; HPA, human primary astrocyte; PHD-2, prolyl hydroxylase 2; qPCR, quantitative polymerase chain reaction; siRNA, small interfering RNA; Tat, transactivator of transcription.

In HPA Tat was found to cause a significant time-dependent up-regulation *(*P* < 0.05) of both the HIF1A mRNA (Fig 10A) and protein (as earlier in Fig 6A and 6B). We next sought to assess whether Tat exposure could also impact the posttranslational regulation of HIF-1α. Under normoxic conditions, prolyl hydroxylase 2 (PHD-2) degrades HIF-1α by prolyl hydroxylation, thereby inhibiting its stability [34]. In the current study, we found that HIV-1 Tat significantly *(*P* < 0.05) down-regulated the mRNA (Fig 10B) and protein (Fig 10C) expression of PHD-2 in a time-dependent manner. Because posttranscriptional modifications could also play a role in the stability of HIF-1A, we next sought to assess the half-life of HIF-1A in HPAs exposed to Tat. HPAs were exposed to Tat for 12 hours followed by treatment with actinomycin D for varying times to block de novo mRNA synthesis, and cell lysates were assessed for expression of HIF-1A RNA. As shown in Fig 10D, in the presence of Tat there was significant increase *(*P* < 0.05) in stability of the HIF-1α mRNA (t_1/2_ = 3.5 hours) compared with cells not exposed to Tat (t_1/2_ = 0.13 hours). We also assessed the half-life of BACE1 mRNA in HPAs exposed to Tat and actinomycin D. As shown in Fig 10E and 10F, in the presence of Tat, similar to HIF-1α, there was significant increase *(*P* < 0.05) in stability of BACE1 mRNA (t_1/2_ = 4.14 hours) compared with cells not exposed to Tat (t_1/2_ = 0.11 hours). To further validate the role of HIF-1A/BACE1-AS binding on the stability of BACE1 mRNA, HPAs were transfected with scrambled/HIF1A/BACE-1AS siRNAs and assessed for BACE1 mRNA stability in the presence of Tat using actinomycin D. As shown in Fig 10E and 10F, in cells knocked down for either HIF-1α or BACE1-AS, there was a significant decrease #(*P* < 0.05) in the stability of BACE1 mRNA compared to Tat-exposed scrambled siRNA-transfected cells. Stability of BACE1-AS remained unchanged in HPAs exposed to Tat (Fig 10G). All the abovementioned statistical analysis was performed using one-way ANOVA followed by Bonferroni post hoc test, for determining significance among different groups.

### In situ hybridization (RNA FISH) of BACE1-AS in the brains of patients infected with HIV

**Fig 11:**
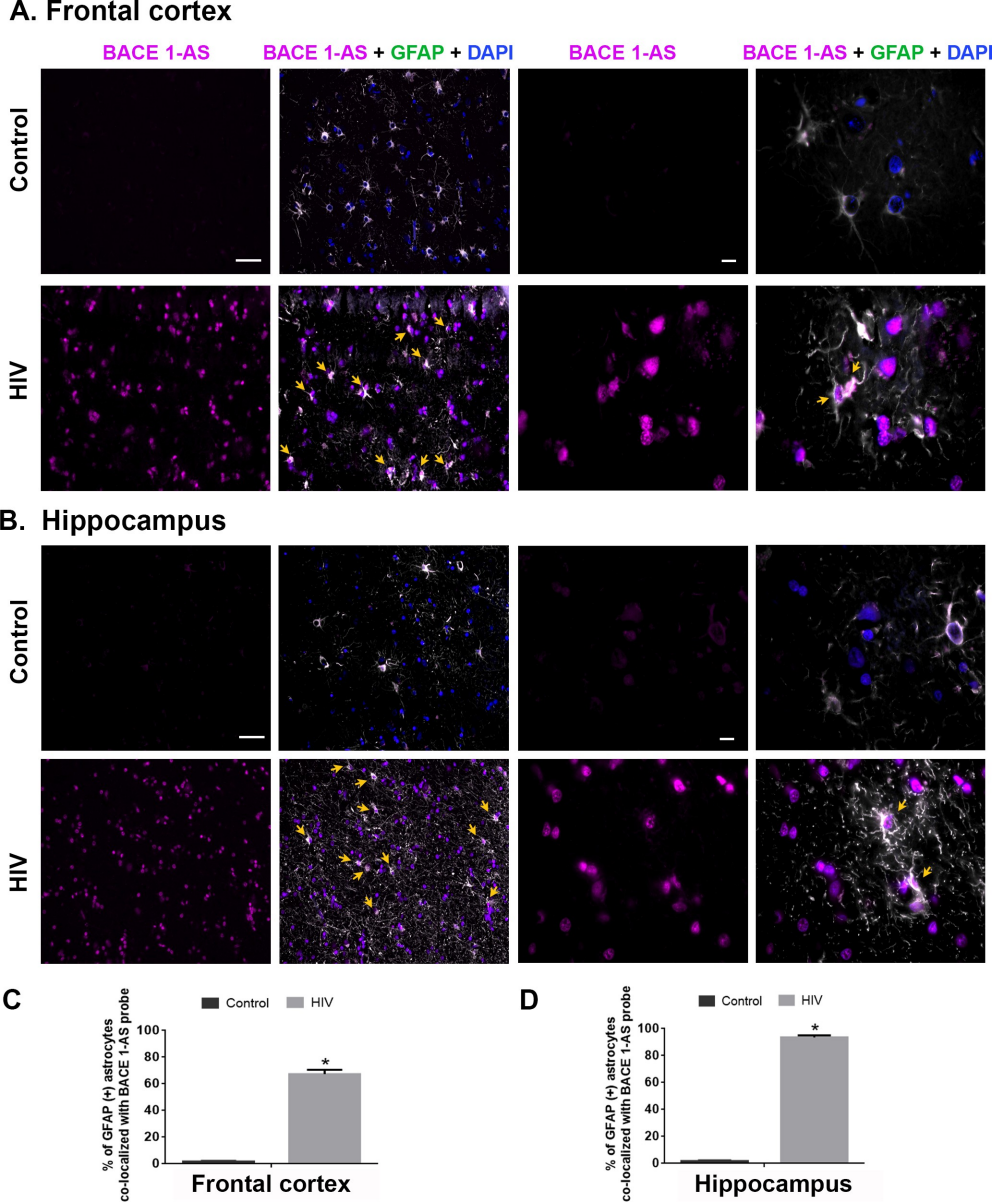
Region-specific expression of BACE1-AS RNA in the brains of patients infected with HIV. Representative FISH and immunofluorecence photomicrographs showing differential expression of BACE1-AS RNA in GFAP^+^ astrocytes in the FC (A) and Hp (B) of control patients and patients who are HIV positive. Scale bar, 10 μm. Quantitative analysis of GFAP^+^ astrocytes (%) colocalizing with BACE1-AS RNA in the FC (C) and Hp (D) of control patients and patients who are HIV positive. Ten fields from FCs per patient were analyzed with *n* = 3 patients. Data are presented as mean ± SEM. Student *t* test was used to determine the statistical significance between multiple groups: *P < 0.05 versus control. Arrows indicate GFAP-positive astrocytes colocalized with BACE1-AS RNA. The data underlying this figure may be found in S11 Data. BACE1-AS, BACE1‐antisense transcript; FC, frontal cortex; FISH, fluorescence insitu hybridization; GFAP, glial fibrillary acidic protein; Hp, hippocampus.

Based on our in vitro findings on the role of BACE-1AS in Tat-mediated induction of amyloidosis, we next sought to validate the expression of BACE1-AS RNA in the section of archival brains from patients infected with HIV. As shown in Fig 11A and 11B there was increased expression of BACE1-AS RNA in the FC and Hp regions of individuals infected with HIV compared with uninfected individuals who exhibited mild or negligible expression of BACE-1AS RNA. In both the FC and Hp, most of the BACE1-AS–expressing cells were also GFAP positive. Quantification data showed a significant increase in BACE1-AS and colocalization *(*P* < 0.05) of GFAP^+^ astrocytes, thereby validating the in vitro findings of BACE1-AS production by astrocytes (Fig 11C and 11D) (as determined by *t* test).

### HIV-1 Tat–induced astrocytic amyloidosis mediated by HIF-1α–lncRNA BACE1-AS pathway

**Fig 12:**
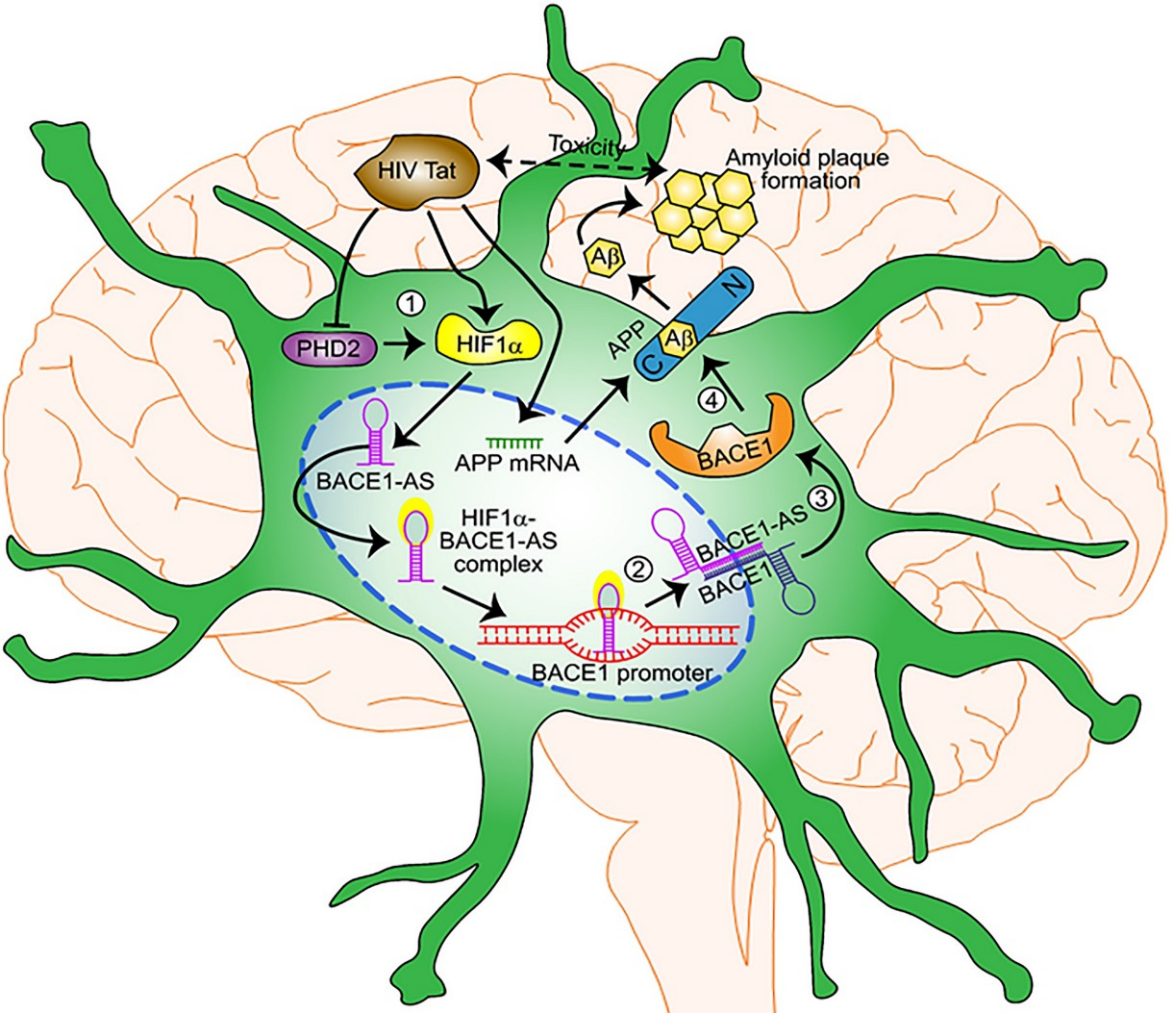
Schematic representation of HIV-1 Tat–induced astrocytic amyloidosis mediated by HIF-1α–lncRNA BACE1-AS axis. HPAs exposed to HIV-1 Tat showed increased expression and accumulation of HIF-1α involving differential regulatory mechanisms—transcription, RNA stabilization (posttranscription), translation, and posttranslational stabilization (decreased expression of PHD-2) (1). Upon accumulation, HIF-1α is translocated into the nucleus, where it forms a complex with the lncRNA BACE1-AS (which is also up-regulated following HIV-1 Tat exposure).The HIF-1α–lncRNA BACE1-AS complex, in turn, binds to the BACE1 promoter to induce its transcription (2). Following transcriptional induction of BACE1 mRNA, it subsequently binds to the BACE1-AS, resulting, in turn, in stabilization of BACE1 mRNA (3). This then leads to increased translation and activity of BACE1 (4). Additionally, HIV-1 Tat has also been shown to induce the expression of APP mRNA, resulting in up-regulated expression of APP protein, which then is subject to cleavage by BACE1, leading to the release of various Aβ forms into the extracellular space. Oligomerization of the toxic Aβ forms leads to deposition of insoluble amyloid plaques in select brain regions, which are neurotoxic and can also independently interact with HIV-1 Tat to further aggravate HAND-associated toxicity. APP, amyloid precursor protein; Aβ, amyloid beta; BACE1, β-site cleaving enzyme; BACE1-AS, BACE1‐antisense transcript; HAND, HIV-associated neurocognitive disorder; HIF-1α, hypoxia-inducible factor; HPA, human primary astrocyte; lncRNA, long noncoding RNA; PHD-2, prolyl hydroxylase 2; Tat, transactivator of transcription. *Original creation*.

HPAs treated with HIV-1 Tat showed an increased expression of HIF-1α by differential mechanisms—transcription, RNA stabilization (posttranscription), translation, posttranslational stabilization (decreased expression of PHD-2). Upon accumulation, HIF-1α is translocated in the nucleus, where it forms a complex with the lncRNA BACE1-AS (which is also up-regulated upon HIV-1 Tat treatment). This complex (HIF-1α–lncRNA BACE1-AS) binds to the promoter of BACE1 to induce its transcription. Following the mRNA expression of BACE1, BACE1-AS binds to it and causes RNA stabilization for long hours. This RNA stabilization results in enhanced translation and activity of BACE1. HIV-1 Tat has also been observed to increase the mRNA expression of APP, resulting in up-regulated protein expression of APP. Hereafter, BACE1 cleaves the APP to release Aβ varieties in the extracellular space. These amyloids oligomerize to form depositions of insoluble amyloid plaques that are neurotoxic and can promote HAND (Fig 12). Fig 12 has been created by us and is an original creation.

## Discussion

Several reports indicate increased numbers of people living worldwide with HIV/AIDS, and among them an estimated 50% exhibit some form of HAND [35]. Despite the dramatic success of combined antiretroviral therapy in suppressing viremia, as the infected individuals continue to live longer, the prevalence of HAND and its association with comorbidities such as AD-like symptomatology are poised to be on the rise. The pathophysiology of observed deficits in HAND remains incompletely understood but has been implicated to be potentiated by aging, consistent with the interaction of aging as observed for most neurodegenerative diseases [36]. Epidemiological studies have also suggested comparable concentrations of Aβ42 in the CSF of HAND patients to those detected in the brains of patients with dementia of the Alzheimer type. The Aβ42 depositions in the patients were mainly found to be intraneuronal and sometimes perivascular [37]. Given the expanding insights for therapeutics to address abnormal Aβ metabolism in AD, clear demonstration of the role of similar pathophysiologic properties in HAND could be of substantial importance. While the role of neuronal deposition of toxic amyloids has been well documented, the contributory role of non-neuronal cells in the deposition of toxic amyloid proteins remains less well studied. The present study for the first time identifies the role of astrocytes in HIV-1 Tat–mediated amyloidosis, with the involvement of the HIF-1α–lncRNA BACE1-AS complex in this process.

Astrocytes are one of the most abundant cell types in the CNS and are involved in several critical physiological functions, such as blood-brain barrier homeostasis, regulation of axonal outgrowth, formation of intracellular communication networks, and inflammation [38] [39–41]. Recent findings have also delineated the role of astrocytes in HIV-mediated neuroinflammation [42–44] and dysregulated autophagy [45]. The present study demonstrated a unique role of astrocytes in HAND. We tested our hypothesis that astrocytes are one of the producers of amyloids in the presence of HIV-1/HIV-1 Tat. We first examined astrocytic amyloidosis ex vivo in the archival tissue sections of SIV-infected rhesus macaques. In the select brain regions of rhesus macaques chronically infected with SIV, there was increased amyloidosis, as evidenced by immunostaining and western blotting for amyloids, specifically in the FC, PC, Hp, basal ganglia (BG), and BS. In contrast, there was no change observed in both the OC and Thal regions in SIV compared with the saline administered macaques. These findings were also validated by RNA-seq data that demonstrated increased expression of APP, BACE1, and HIF-1α RNA in the brains of SIV-infected macaques. We also validated these findings by immunostaining, wherein there was increased amyloid deposition in the GFAP^+^ astrocytes in the brains of SIV-infected macaques (S1 Fig). Using in situ hybridization, we demonstrated the expression of APP RNA in the astrocytes of SIV-infected macaque brains, thus underscoring the process of astrocytic amyloidosis in the archival brain sections of SIV-infected macaques. Additionally, Aβ 1–40, HIF-1α, and BACE1 were also observed to be differentially up-regulated in macaque brain regions (S2 and S3 Figs).

Increased p-Tau expression (pathophysiological marker for neurodegeneration in AD) was also observed in the brain regions of SIV-infected macaques (S4 Fig). Previous reports by Mankowski and colleagues (2002) [46] had demonstrated the presence of β-APP in the astrocytes and in the neurons of SIVgp41-infected macaque brains. Similar to these findings, we also observed the presence of amyloids in the astrocytes of SIV/HIV-infected brains, with a region-specific expression. Although amyloidopathy has been well studied in HIV-infected brains [8,47,48], this study for the first time demonstrates the up-regulated expression of AD markers such as APP, Aβ, p-Tau, and BACE1 that can be associated with HAND. We found increased expression of the AD markers in both individuals infected with HIV with asymptomatic neurocognitive impairment, as well as in the cognitively impaired group. Validation of these markers was also done in the positive control groups that included individuals infected with HIV with AD pathology as well as those with ischemic brain damage (also HIV^+^).

These data thus underscore the role of amyloid pathogenesis in HIV infection. The presence of these markers in the brains of individuals infected with HIV with asymptomatic neurocognitive impairment further implicates that these markers can likely serve as biomarkers for the development of cognitive impairment in HAND patients on ART therapy. Another interesting aspect of this study was the observation of significant down-regulation of the Aβ40/42 ratio in both asymptomatic as well as cognitively impaired patients diagnosed with HIV, thereby confirming increased expression of the neurotoxic Aβ42 form in individuals infected with HIV with varying degrees of HAND. In situ hybridization coupled with immunostaining showed clear presence of APP RNA in GFAP-positive astrocytes in both the FC and Hp, thereby confirming the process of astrocytic amyloidosis in cognitively impaired patients diagnosed with HIV. In addition to GFAP^+^ cells, other cells (GFAP negative) also showed significant amyloid production, thereby suggesting multiple sources of amyloids producing cells in the CNS. Based on the abililty of neurons to also express amyloids, we quantified the contribution of both astrocytes and neurons in amyloidosis in the brain sections of SIV^+^ macaques. As shown in S6 Fig, both neurons and astrocytes contributed about equally to the process of amyloidosis, as evidenced by FISH, using the APP probe.

Further mechanistic exploration of the process of astrocytic amyloidosis involved use of an in vitro model of HPAs stimulated with HIV-1 Tat followed by assessing the synthesis and secretion of various forms of the amyloid proteins (APP, Aβ 42), the synthesizing enzyme—BACE1 and its activity. Similar to findings in vivo, we also found that exposure of HPAs to HIV-1 Tat (as a surrogate of HIV infection) resulted in up-regulation of the toxic amyloid forms, thereby underpinning the role of HIV-1 Tat in astrocytic amyloidosis. To decipher whether the amyloids present in the astrocytes were a result of increased astrocytic phagocytosis or that the cells inherently produced increased amyloids, we performed the phagocytosis assay in HPAs cultured in presence or absence of HIV-1 Tat. As shown in S9 Fig, HPAs cultured in the presence of HIV-1 Tat exhibited minimal phagocytic activity, thus leading to the speculation that astrocytic amyloidosis in presence of HIV-1 Tat was a result of HIV-1 Tat-mediated production of amyloids and not due to astrocytic phagocytic activity.

While reports on the role of HIV-1/ HIV-1 Tat in neuronal amyloidosis are extant [13,37], this is the first report of Tat-mediated glial amyloidosis. Experiments with several studies have also shown the involvement of ischemic brain damage in amyloidosis [49,50].

Additionally, the involvement of hypoxia-induced HIF-1α has been well documented in amyloidosis associated with AD [27,51]. Similar to these findings, we have also observed that HIV-1 Tat could up-regulate the expression of HIF-1α in HPA, leading to its nuclear translocation (Fig 6 and S7 Fig), in turn, resulting in the binding of HIF-1α to lncRNA BACE1-AS promoter leading to its increased synthesis (Figs 7–9). Using various approaches such as EMSA and RIP assay, we also validated the physical binding of HIF-1α to lncRNA BACE1-AS. Up-regulation of BACE1-AS was also observed in the HIF-1α RNA–enriched component (RIP assay) in Tat-exposed astrocytes compared to that of control by RNA sequencing, thus further validating the physical interaction of HIF-1α with BACE-1AS. EMSA data demonstrated clear binding of the in vitro transcribed BACE1-AS RNA with the purified HIF-1α protein. It must be noted that structural simulation of the HIF-1α protein also revealed several motifs that could bind with the BACE1-AS RNA. Additionally, using the gene silencing approach we have also demonstrated HIF-1α as a master regulator and an upstream component that regulates the expression of BACE1-AS. Furthermore, our findings also showed that the HIF-1α and BACE1-AS complex could regulate BACE1 synthesis both transcriptionally and posttranscriptionally, as confirmed by ChIP assay. Additionally following the synthesis of BACE1, BACE1-AS can further bind with the overlapping region of BACE1 mRNA to increase its stability. In keeping with these findings, BACE1-AS mediated stability of BACE1 mRNA has also been demonstrated in an animal model of AD [26]. Multiple mechanisms of increased expression and stability of the of APP cleaving enzyme BACE1 [52] could thus contribute to increased cleavage and release of the toxic 42 form via the cleavage of APP. The role of Aβ42 as a neurotoxic protein in cognitive impairment associated with AD has been well documented [52].

Next, we sought out to explore the upstream mechanisms involved in Tat-mediated induction of HIF-1α expression. Tat could induce the expression of HIF-1α in a multistep process involving transcription and posttranscriptional and translational, as well as posttranslational, stability by decreasing the expression of PHD-2 enzyme, which is involved in degradation of HIF-1α under normoxic conditions. To further implicate the role of PHD-2 in HIF-1–mediated amyloidosis, we used gene silencing and overexpression approaches. As shown in S8 Fig, silencing of PHD-2 up-regulated HIF-1α–mediated amyloidosis and, in contrast, overexpressing PHD-2 mitigated this process. This is a very novel finding, as the role of PHD-2 in amyloidosis has not been reported earlier. This opens up future avenues for research in amyloid pathophysiology. Further, it was also shown that Tat-induced stability of BACE1 mRNA was regulated by the HIF-1α and BACE1-AS complex. Tat failed to induce the stabilization of BACE1-AS (Figs 6 and 10). Further research on identification of other additional targets of HIF-1α that could be regulated by BACE1-AS RNA is warranted. Using FC and Hp of both SIV-infected monkeys and patients infected with HIV, we also demonstrated the presence and colocalization of BACE1-AS RNA in GFAP-positive astrocytes using the in situ hybridization approach (Fig 11 and S5 Fig). Several studies have shown different regulators of HIF-1α [53], e.g., HIV-1 infection and proteins such as Tat elevate levels of reactive oxygen species [54–56], which in turn was shown to induce the expression and activation of HIF-1α [57–59], the Factor Inhibiting HIF (FIH), an asparaginyl hydroxylase, suppresses HIF-1 transcriptional activity in normoxia by preventing co-activator recruitment [60,61], WD repeat and SOCS box-containing protein 1 (WSB1), was also found to promote HIF-1α stabilization [62]; additionally, the receptor of activated protein C kinase, heat shock protein, or Sentrin/SUMO-specific protease 1 [63–65] was also reported to regulate the HIF-1 promoter. Transcription and translation of HIF-1α has also shown to be regulated by major pro-survival pathways, namely extracellular-signal-regulated kinase (ERK)/ mitogen activated protein kinase (MAPK), Janus kinase-signal transducer and activator of transcription (JAK/STAT), and phosphatidylinositol 3-kinase (PI3K)/ Akt/ mammalian target of rapamycin (mTOR) [66]. HIV-1 infection and protein-induced HIF-1α mediated metabolic alteration have also been shown to dysregulate cellular functionality through metabolic alteration [58]. Expression of HIF-1α can thus be regulated by different mechanisms in the presence of HIV-1 Tat; however, in this study we have demonstrated the direct mechanisms involving Tat-mediated HIF-1α regulation. Additionallly, HIF-1α and BACE 1 were also observed to be up-regulated differentially in macaque brain regions (S2 Fig).

In brief, our studies implicate that HIV-1 Tat/HIV can induce the expression and stability of the transcription factor HIF-1α, which, upon translocation into the nucleus, binds to the BACE1-AS RNA to induce its synthesis. The HIF-1α–BACE1-AS complex, in turn, regulates the expression of BACE1 via multistep processes, resulting in increased expression and activity of the BACE1 enzyme, which subsequently leads to cleavage of the transmembrane protein APP (up-regulated independently by Tat) to its toxic Aβ forms. These findings are in keeping with the published reports by Hategan and colleagues, wherein Tat and amyloid beta aggregates were shown to form multifibrillar structures both in vitro and in animal models [19]. There are, however, conflicting reports about deposition of fibrillar brain amyloids in individuals infected with HIV with HAND, as assessed by MRI using Pittsburgh Compound B. In this study, the authors found no correlation between low levels of CSF Aβ 42 and Pittsburgh Compound B mean cortical binding potential (MCBP)in individuals infected with HIV. On the other hand, patients diagnosed with AD with symptomatology exhibited Pittsburgh Compound B positive plaques. A possible explanation for this discrepancy could be attributed to several factors, including the age of the patients, sample size, region of the brain assessed, and methods for collection, as mentioned by the authors themselves [67–69], while many other studies reports amyloid deposition in the brains of HAND patients [9,15,48,70–76] that are in line with our study. Although both AD and HAND brains show deposition of plaques, there are marked differences in plaque deposition in HAND and AD patients [17]. AD has been characterized by deposition of senile plaques [77], while diffused neuritic plaques are observed in HAND [78,79]. Interestingly, soluble amyloids either remain unaltered or increased in AD [77], while in HAND patients, soluble amyloids are decreased [78].

While the focus of this study is on astrocytic amyloidosis, the contribution of neuronal amyloidosis cannot be overruled. Our cell culture findings indicate that HIV-1 Tat induces astrocytic amyloidosis as an early event (12 hours) while that in the human neurons this is a later event, with induction of amyloidosis approximately 48 hours post HIV-1 Tat exposure (S10 Fig). These findigns are in keeping with a previous study by Chen and colleagues, 2013 [14], also demonstrating amyloid production in neurons approximately 48 hours post HIV-1 Tat exposure. It can thus be speculated that astocytic amyloidosis is an early trigger by HIV-1 Tat leading to increased accumulation of these toxic isoforms, which in turn could be redistributed to different sites and cells types by extraellular vesicle–mediated seeding [80]. Another striking difference noted between Tat-mediated amyloidosis in neurons versus astrocytes is the unique involvement of HIF-1α and PHD-2 in astrocytes versus the neurons (S10 Fig). While the process of amyloidosis is well documented in the neurons, this is the first report of Tat/HIV/SIV-mediated induction of astrocytic amyloidosis and is associated with neurocognitive impairment involving the HIF-1α–BACE1-AS complex. The HIF-1α–BACE1-AS lncRNA complex can be targeted for future development of adjunctive therapies for HAND patients on cART therapy.

## Materials and methods

### Reagents

The following antibodies or reagents used in this study were from the indicated sources: reagents: HIV-1 Tat 101 (Immunodiaognistics, Woburn, MA), cobalt chloride (Sigma-Aldrich, *St*. *Louis*, *MO*, 232696), Actinomycin D (Sigma, *St*. *Louis*, *MO*, A9415), SP6/T7 Transcription Kit (Sigma, St. Louis, MO, 10 999 644 001), Biotin-16-UTP (Sigma, St. Louis, MO, 11 388 908 910), HindIII (NEB, Ipswich, MA,R0104S), EcoRI (NEB, Ipswich, MA, R0101S), Tth111I (NEB, Ipswich, MA, R0185S), Platinum Taq DNA Polymerase High Fidelity (ThermoFisher, Waltham, MA,11304011), 10 mM dNTP mix (ThermoFisher, Waltham, MA, 18427–088), TrackIt 1 Kb Plus DNA Ladder (ThermoFisher, Waltham, MA, 10488–085), QIAquick Gel Extraction Kit (Qiagen, Hilden, Germany, 28704), NEB 5-alpha Competent E. coli (High Efficiency) (NEB, Ipswich, MA, C2987I), T4 DNA Ligase (NEB, Ipswich, MA, M0202S), Light Shift Chemiluminescent RNA EMSA (REMSA) Kit (Thermo, Waltham, MA, 20158), HIF1A purified protein Recombinant Human HIF-1 alpha protein (Abcam, Cambridge, MA, ab154478), DEPC (Sigma, St. Louis, MO, 40718), Positive charged nylon membrane (Ambion, Austin, TX, AM10104), ssRNA Ladder (NEB, Ipswich, MA, N0364S), RNA loading dye (NEB, Ipswich, MA, BO363), 10X MOPS buffer (KD Medical, Columbia, MD, RGF-6170), 10X TBE Electrophoresis buffer (Thermo Scientific, Waltham, MA, B52), Formaldehyde (Sigma, St. Louis, MO, 252549), RNA gel extraction kit (Zymo Research, R1011); antibodies: HIF-1α (Novus Biological Company, Littleton, CO, NB100-449, Millipore Sigma, St. Louis, MO,MAB5382MI), APP (Abcam, Cambridge, MA, ab15272), AβmOC64 (Abcam, Cambridge, MA, ab201060), Aβ 1–42 (Abcam, Cambridge, MA, ab10148), Aβ 1–40 (Abcam, Cambridge, MA, ab76317), BACE1 (Abcam, ab63954), p-Tau (Abcam, Cambridge, MA, ab76128), Tau (Abcam, Cambridge, MA, ab9778), MAP 2 (Abcam, Cambridge, MA, ab5392), GFAP (Sigma-Aldrich, St. Louis, MO, G3893), Lamin B1 (Abcam, Cambridge, MA, ab8982), PHD-2 (Abcam, Cambridge, MA, ab133630), goat anti-rabbit (sc-2004, Santa Cruz Biotechnology, Dallas, TX), goat anti-mouse (sc-2005, Santa Cruz Biotechnology, Dallas, TX), and β-actin (Sigma Aldrich, St. Louis, MO, A5316) were purchased from commercial vendors, as mentioned. HA-HIF1 alpha-pcDNA3 was a gift from William Kaelin (Adgene, Watertown, MA, plasmid #18949).

### Macaque studies

Archival brain tissues from rhesus macaques were used in this study. Briefly, the groups included four control (saline-injected) macaques and three SIVR71/17E chronically infected macaques (approximately 52 weeks). Details of virus load and disease pathogenesis have been described previously [81,82]. After 52 weeks of saline or SIV infection, the animals were humanely killed and different brain regions were dissected, namely FC, PC, OC, BS, striatum, Thal, and Cer for further experimentation.

### Human studies

Human brain samples were received from The National NNTC. The human patient samples were from donors 45–60 years of age. They were divided into 5 groups: Group 1 (HIV^−^, *n* = 3), Group 2 (HIV^+^, asymptomatic neurocognitive impairment, *n* = 4), Group 3 (HIV^+^, mild neurocognitive disorder, *n* = 4), Group 4 (HIV^+^, hypoxic/ ischemic damage, *n* = 4), Group 5 (HIV^+^, neurofibrillary pathology, *n* = 4). The samples used were from individuals with no diagnosable abuse/dependence on drugs (cocaine, opiate, methamphetamine) and did not suffer from CNS infections or encephalitis. Frozen samples from FC were used for the determination of protein expression, while the paraffin fixed sections from the FC/Hp were used for immunostaining and in situ hybridization.

### Cell culture

HPA were obtained from ScienCell Research Laboratories (Carlsbad, CA, 1800) and were cultured in astrocyte media with 2% FBS (ScienCell Research Laboratories, Carlsbad, CA, 0010), 1% astrocyte growth supplement (ScienCell Research Laboratories, Carlsbad, CA, 1852) and 1% penicillin-streptomycin solution (ScienCell Research Laboratories, Carlsbad, CA, 0503) in a 5% CO_2_-humidified incubator at 37°C. HPAs were used up to 5 passages as per the manufacturer’s protocol at seeding densities of 0.3 × 10^6^/well for a 6-well plate, 0.05 × 10^6^/well for a 24-well plate, and 2 × 10^6^ for a 100-mm petri dish. Cells were serum starved prior to exposure to HIV-1 Tat.

### Real-Time qPCR

Total RNA was isolated from the HPA and FC of rhesus macaques brain tissues using Quick-RNA MicroPrep kit (Zymo Research Corporation, Irvine, CA, R1051) as per the manufacturer's protocol. One microgram of total RNA was used for the synthesis of complementary DNA, and RT-PCR was performed as described previously [83,84]. The commercial primers (Thermo Fisher Scientific, Waltham, MA,) used for HPA–APP (Hs_00169098), BACE1 (Hs_01121195), BACE1-AS (Hs_04232267), HIF-1α (Hs_00153153), ELGN1 (Hs_00254392), GAPDH (Hs_002786624), RNU2 (Hs_02786874), VEGF A (Hs00900055_m1), MMP-2 (Hs01548727_m1), *BACE1 promoter* forward 5′-GGCCACCAGTTGGATTCAGCTT-3′, and *BACE1 promoter* reverse 5′-GGAAACAGGGAAGGTGCGGTT-3′ were designed using Oligo Perfect Designer, an online-based software (Life Technologies, Carlsbad, CA), and were synthesized from the same company. Normalization was done with *GAPDH*, and the fold change in expression was obtained. The specificity of the RT-qPCR was controlled using a non-template control.

### Western blotting

HPAs were washed once with PBS and lysed using the Mammalian Cell Lysis kit (Sigma-Aldrich, St. Louis, MO, MCL1-1KT). After centrifugation of the cell lysates at 12,000*g* for 10 minutes at 4°C, the protein content of the supernatant was quantified by a BCA assay using Pierce BCA Protein Assay Kit (Thermo Fisher Scientific, Waltham, MA, 23227) according to the manufacturer’s protocol. Similarly, brain lysates from SIV-infected monkeys and patients infected with HIV were homogenized in Mammalian Cell Lysis buffer, centrifuged at 12000*g* for 10 minutes at 4°C, and the protein content of the supernatant was quantified by a BCA assay using Pierce BCA Protein Assay Kit, followed by the performance of western blot, as described previously (62). Image J (v1.4.3.67; NIH, Bethesda, MD) software was used for quantification [85].

### ELISA

HPAs (0.3 × 10^**6**^ cells per well) were seeded into 6-well plates and incubated overnight at 37°C in a humidified, 5% CO_**2**_ incubator. The next day, after serum starvation, cells were treated with 3.57 nM of HIV-1 Tat, and the cell lysates as well as the supernatant fluids were collected after 0, 3, 6, 12, 24, 48, 72, and 96 hours of treatment. Similarly, cell supernatants were also collected after transfection of cells with different targeted siRNAs (HIF1α, BACE1, BACE1-AS, and BACE1–BACE1-AS) and HIV-1 Tat exposure. Cell lysates and supernatant fluids were used for Aβ 42 detection by ELISA using a human Aβ 42 ELISA kit (Thermo Fischer Scientific, Waltham, MA, KHB3544) according to the manufacturer’s instructions. Human FC tissues were homogenized in PBS, centrifuged, and the supernatants were used for detection of Aβ 42 and Aβ 40 by ELISA using a human Aβ 42 (Thermo Fischer Scientific, Waltham, MA, KHB3544) and Aβ 40 ELISA kit (Thermo Fischer Scientific, Waltham, MA, KHB3482), respectively, according to the manufacturer’s instructions.

### β secretase activity

HPAs were cultured in 6-well plates in specified conditions, as mentioned before, and starved at 70% confluency. After 24 hours of HIV-1 Tat exposure to HPA, cells were washed once in 1× cold PBS and resuspended in 100 μL extraction buffer, and assessed for BACE1 activity using the Beta-Secretase Activity Assay Kit fluorometric kit (Abcam, Cambridge, MA, ab65357) as per the manufacturer’s protocol.

### Nuclear and cytoplasmic extraction

HPAs were cultured in 6-well plates in specified conditions, as mentioned before, and starved at 70% confluency. After 24 hours of HIV-1 Tat exposure to HPA, cells were washed with 1× cold PBS, harvested, and centrifuged at 500*g* for 5 minutes, and the nucleus/cytoplasmic protein fractions were extracted using the NE-PER Nuclear and Cytoplasmic Extraction Reagents kit (Thermo Fischer Scientific, Waltham, MA, 78833) as per the manufacturer’s protocol.

HPA under similar culture conditions and after 12 hours of HIV-1 Tat treatment were washed with 1× cold PBS, harvested in ice-cold lysis solution, and the nucleus/cytoplasmic RNA fractions were extracted using the SurePrep Nuclear or Cytoplasmic RNA Purification Kit (Thermo Fischer Scientific, Waltham, MA, BP2805-25) as per the manufacturer’s protocol.

### Immunocytochemistry

HPAs were seeded on the coverslips (11-mm) in a 24-well plate at a density of 0.05 × 10^6^ cells per well at 37°C in a humidified, 5% CO_2_ incubator for 24 hours. Upon confluency, serum-starved HPAs were transfected with HIF-1α–overexpressing plasmid and were exposed to HIV-1 Tat (3.57 nM) for 24 hours, followed by fixation of cells and incubation with respective antibodies, as described previously [62]. Fluorescence images were captured on a Zeiss Observer using a Z1 inverted microscope (Carl Zeiss, Thornwood, NY) and analyzed using the AxioVs 40 Version 4.8.0.0 software (Carl Zeiss MicroImaging GmbH).

### Immunohistochemistry

Formalin-fixed paraffin-embedded (FFPE) tissue sections of different brain regions of saline, SIV^**+**^ macaques, and control and HIV^**+**^ brain sections were deparaffinized and rehydrated using decreasing percentage of ethanol followed by antigen retrieval, blocking, and incubation with respective antibodies, as described previously [62]. Fluorescent images were acquired using Z1 inverted microscope (Carl Zeiss, Thornwood, NY) and analyzed using the AxioVs 40 Version 4.8.0.0 software (Carl Zeiss MicroImaging GmbH).

### RNA sequencing

Total RNA was extracted from FCs of the above monkeys, control and Tat-treated HPA samples, using Quick-RNA MicroPrep kit (Zymo Research Corporation, R1051) as per the manufacturer's protocol. Similarly, control and Tat-exposed HPA were used to perform RIP assay: HIF-1α pulled-down RNA immunoprecipitated samples were used for RNA sequencing, as well. cDNA libraries were generated with the mRNA-Seq sample preparation kit (Illumina, San Diego, CA). The paired-end sequencing was performed on an Illumina Hiseq 4000 (LC Bio, China) following the vendor's recommended protocol. StringTie [86] was used to determine the expression levels of mRNAs by calculating the FPKM. Differentially expressed mRNAs were selected with log_2_ (fold change) >1 or log_2_ (fold change) <−1 and with statistical significance (*P* < 0.05) by R package Ballgown [86]. Heatmaps were generated in R using the heatmap.2 function from the gplots package.

### mRNA stability assay

For mRNA stability assays 10 μg/mL actinomycin D (Act D, Sigma-Aldrich, St. Louis, MO) was added to cultured HPAs in the presence or absence of HIV-1 Tat (3.57 nM) for 0, 0.25, 0.5, 1, 1.5, 3, 6, 12, 24 hours. A similar protocol was followed after transfection with either HIF-1α or BACE1-AS siRNA. At selected time points following Act D treatment, total RNA was isolated and HIF-1α/BACE1/BACE1-AS RNA expression was detected by real-time RT-qPCR. Fold change in gene expression determined from RT-qPCR assay was then used to calculate the percentage of mRNA remaining, following Act D treatment. For exponential one-phase decay, nonlinear regression was used to calculate the half-life of the mRNA and plot the graph.

### siRNA transfection

HPAs were seeded in 6-well plates (0.3 × 10^6^ cells per well) and incubated overnight at 37°C in a humidified, 5% CO_2_ incubator. The next day, cells were transfected with either human *HIF-1α* siRNA (Santa Cruz Biotechnology, Dallas, TX, sc-35561), human *BACE1* siRNA (Santa Cruz Biotechnology, Dallas, TX, sc-37224), *BACE1-BACE1-AS* siRNA (Thermo Fischer Scientific, Waltham, MA, assay ID: 148407),*BACE1-AS* siRNA (Integrated DNA Technologies, Coralville, IA, customized), or HIF-1α–overexpressing plasmid (Adgene, Watertown, MA, 18948), as described previously [62]. Knockdown efficiencies were confirmed by western blotting and qPCR.

### In situ hybridization (RNA FISH and Immunofluorescence)

Custom Stellaris FISH Probes were designed against BACE1 antisense (BACE1-AS) RNA and APP mRNA by utilizing the Stellaris FISH Probe Designer (Biosearch Technologies, Petaluma, CA), available online at www.biosearchtech.com/stellarisdesigner. The human and macaque FFPE tissue sections were hybridized with the Stellaris FISH Probe set labeled with 670 Dye (Biosearch Technologies Petaluma, CA,), following the manufacturer’s instructions available online at www.biosearchtech.com/stellarisprotocols. Briefly, FFPE tissue sections of different brain regions of saline, SIV^+^ macaques, control, and HIV^+^ brain sections were deparaffinized and rehydrated using a decreasing percentage of ethanol. Antigen retrieval was performed by boiling the slides in 0.01 M Tris-EDTA buffer, pH 9, for 40 minutes. The brain sections were permeabilized with 0.1% Triton X-100 in 1X PBS for 10 minutes at room temperature, followed by blocking with 10% goat serum for 1 hour. After blocking, the sections were incubated with GFAP (1:500) primary antibody for 1 hour at room temperature. Secondary Alexa Fluor 594 goat anti-mouse and Fluor Alexa Fluor 488 goat anti-chicken were added for 1 hour, followed by hybridization as per the Stellaris FISH manufacturer’s protocol, and after, the brain sections were mounted with Prolong gold anti-fade reagent DAPI. Fluorescent images were acquired using Z1 inverted microscope (Carl Zeiss, Thornwood, NY) and analyzed using the AxioVs 40 Version 4.8.0.0 software (Carl Zeiss MicroImaging GmbH).

### RIP assay

HPAs were seeded in a 100-mm petri dish at the density of 2 × 10^6^ cells and incubated overnight at 37°C in a humidified, 5% CO_2_ incubator. Cells were serum starved at approximately 70%–80% confluency, followed by exposure to HIV-1 Tat. Control and HIV-1 Tat–treated HPAs were harvested and RIP assay was performed with EZ-Magna RIP kit (Millipore Sigma, St. Louis, MO, 17–701) as per the manufacturer’s protocol. HIF-1α antibody (Millipore Sigma, St. Louis, MO, MAB5382) was used to perform the immunoprecipitation of RNA-binding protein/RNA complexes. Isolated RNAs from the input and enrichment fractions were used to either prepare cDNA using the verso cDNA synthesis kit (Thermo Fischer Scientific, Waltham, MA, AB 1453A) or for RNA sequencing. The reverse transcribed RNA was analyzed with the 7500 Fast Real-Time PCR System (Applied Biosystems, Grand Island, NY) with the TaqMan Universal PCR Master Mix (Applied Biosystems, Grand Island, NY 4304437, Thermo Fisher Scientific, Waltham, MA,), and BACE1-AS primers were used for qRT-PCR.

### ChIP assay

Similar to the RIP assay, HPAs were seeded in a 100-mm petri dish at the density of approximately 2 × 10^6^ cells and incubated overnight at 37°C in a humidified, 5% CO_2_ incubator. Cells were serum starved at approximately 70%–80% confluency, followed by HIV-1 Tat exposure. Control and HIV-1 Tat–treated HPAs were harvested, and ChIP assay was performed using the EZ-Magna ChIP A/G ChIP kit (Millipore Sigma, St. Louis, MO, 17–10086) as per the manufacturer’s protocol. HIF-1α antibody (Millipore Sigma, St. Louis, MO, MAB5382) was used to perform the immunoprecipitation of DNA-binding protein/DNA complexes. Isolated DNAs were analyzed using the 7500 Fast Real-Time PCR System (Applied Biosystems, Grand Island, NY) with the RT^2^ SYBR Green Fluor qPCR Mastermix (Qiagen, Hilden, Germany, 330510) or TaqMan Universal PCR Master Mix (Applied Biosystems, 4304437, Thermo Fisher Scientific, Waltham, MA,) for the BACE1 promoter. PCR products were subjected to agarose gel electrophoresis to confirm the binding of HIF-1α to the promoter of BACE1.

### Cloning

The full length of BACE1-AS cDNA was amplified with forward (BACE1-AS_F840: ATAcaagcttgGGCTCACCGCAACCTCCACCGT containing a HindIII site) and reverse (BACE1-AS_R840: CCGgaattcAAAAGCACTAAACAAGGTATTTTTATTTTCAAGTTTGG containing an EcoRI site) primers. The PCR product was digested with HindIII and EcoRI and ligated into similarly digested p18.

### In vitro transcription

Plasmid DNA (full length—840 bp) was linearized with EcoR1, and biotin-labeled RNA/unlabeled RNA was synthesized using SP6/T7 Transcription Kit (Sigma, St. Louis, MO, 10 999 644 001) as per the manufacturer’s protocol. Hereafter, purified RNA was run in an RNA gel to confirm the purity and size. Thereafter, gel containing RNA was excised and purified using RNA gel extraction kit (Zymo Research, Irvine, CA, R1011).

### EMSA

EMSA was performed using purified HIF-1 recombinant protein, nuclear extracts of Tat 101–exposed, and HIF-1α–overexpressing HPA and purified labeled/unlabeled BACE1-AS RNA (*Eco*R I)–digested 840 bp cloned BACE1-AS DNA sequence followed by in vitro transcription, using Light Shift Chemiluminescent RNA EMSA (REMSA) Kit (Thermo, Waltham, MA, 20158) as per the manufacturer’s protocol. To ascertain the binding of HIF-1α with BACE1-AS, HIF-1α–neutralizing antibody was used.

### Statistical analysis

The data are represented as mean ± SEM. One-way ANOVA followed by Bonferroni post hoc test was employed to compare within the multiple experimental groups, Student *t* test was employed to compare between two groups, and nonlinear regression was performed using the GraphPad Prism software (Version 5). For in vitro study, three replicates per sample and six independent sets of experiments were analyzed. Statistical analysis for which probability levels were less than 0.05 were considered statistically significant.

### Ethics statement

Rhesus macaques (*Macaca mulatta*) were purchased from the Caribbean Research Primate Center and individually housed in two dedicated rooms within the AAALAC-approved animal facility at the University of Kansas Medical Center. The monkeys were housed under 12-hour light–dark cycles and given laboratory chow and water ad libitum along with daily snacks. Animals were also given a variety of environmental enrichments. The animals were selected as negative for tuberculosis, herpes B virus, and simian retrovirus. The animal protocol (2009–1815) was approved by the local animal care committee (IACUC) at the University of Kansas (the corresponding author was previous affiliated to this institute) in accordance with the *Guide for the Care and USE of Laboratory Animals*.

The cases for this study were selected from an archive of banked brain specimens in the NNTC. NNTC studies were conducted in accordance with human patient protection protocols at participating institutions. The following offices maintained the institutional review boards (IRBs) that provided oversight for the protection of human subjects: (1) Texas NeuroAIDS Research Center (TNRC) under the University of Texas Medical Branch at Galveston, Texas: IRB-approved projects 98–402 and 03–195. TNRC operates under Federal Wide Assurance number FWA 00002729 on file with the United States Department of Health and Human Services (DHHS). (2) California NeuroAIDS Tissue Network (CNTN) under University of California, San Diego (UCSD) IRB-approved projects 171024 and 080323. UCSD operates under Federal Wide Assurance number FWA00004495 on file with the US DHHS. The UCSD IRBs are registered with DHHS under IORG number 0000210. (3) National Neurological AIDS Bank (NNAB) under the University of California at Los Angeles, David Geffen School of Medicine at Los Angeles, CA, IRB-approval number 10–000525. NNAB operates under Federal Wide Assurance number FWA 00004642 on file with the US DHHS. (4) Manhattan HIV Brain Bank (MHBB) under Icahn School of Medicine at Mount Sinai (ISMMS) IRB-approved project HS11-00388. ISMMS operates under Federal Wide Assurances FWA00005656 and FWA0005651 on file with the US DHHS. Written informed consent was obtained for all patients prior to autopsy.

## Supporting information

S1 Text Expression of Aβ 1–42 and co-localization with GFAP in brain regions of SIV-infected macaques. Aβ, amyloid beta; GFAP, glial fibrillary acidic protein; SIV, simian immunodeficincy virus.
(DOCX)

S2 Text Expression of HIF-1α and BACE1 in SIV-infected macaques. BACE1, β-site cleaving enzyme; HIF-1α, hypoxia-inducible factor; SIV, simian immunodeficincy virus.
(DOCX)

S3 Text Differential expression of Aβ1–40 in the brains of SIV-infected macaques. Aβ, amyloid beta; SIV, simian immunodeficincy virus.
(DOCX)

S4 Text Differential expression of p-Tau in the brains of SIV-infected macaques. SIV, simian immunodeficincy virus.
(DOCX)

S5 Text In situ hybridization (RNA FISH) of BACE1-AS RNA in the brains of SIV-infected macaques. BACE1-AS, BACE1‐antisense transcript; RNA FISH, RNA fluorescent insitu hybridization; SIV, simian immunodeficiency virus.
(DOCX)

S6 Text In situ hybridization (RNA FISH) of APP RNA in the brains of SIV-infected macaques. APP, amyloid precursor protein; RNA FISH, RNA fluorescent insitu hybridization; SIV, simian immunodeficiency virus
(DOCX)

S7 Text Increased Tat-mediated nuclear translocation of HIF-1α in HIF-1α–overexpressing HPA. HIF-1α, hypoxia-inducible factor; HPA, human primary astrocyte; Tat, transactivator of transcription
(DOCX)

S8 Text PHD-2 in HIV-1 Tat-mediated amyloidosis. PHD-2, prolyl hydroxylase 2; Tat, transactivator of transcription
(DOCX)

S9 Text Phagocytic activity of HIV-1 Tat–exposed HPAs. HPA, human primary astrocyte; Tat, transactivator of transcription
(DOCX)

S10 Text Amyloidosis in HIV-1 Tat–exposed neurons. Tat, transactivator of transcription
(DOCX)

S1 Fig Brain region–specific expression of Aβ1–42 in SIV-infected macaques. Representative fluorescent photomicrographs showing differential expression of Aβ 1–42 in GFAP^+^ astrocytes in the different brain regions of saline and SIV^+^ macaques. Scale bar, 10 μm. Saline, *n* = 4; SIV, *n* = 3. Aβ, amyloid beta; GFAP, glial fibrillary acidic protein; SIV, simian immunodeficincy virus.
(TIF)

S2 Fig Expression of HIF-1α and BACE1 protein in the brain regions of SIV-infected macaques. (A) Representative western blots showing the expression of HIF-1α in different brain regions—FC, PC, Cer, BS, OC, and Thal of saline- and SIV-infected macaques. (B) Representative western blots showing the expression of BACE1 in different brain regions—FC, PC, Cer, BS, OC, and Thal of saline- and SIV-infected macaques. β-actin was used as an internal control. *n* = 6. Data are presented as mean ± SEM; saline, *n* = 4; SIV, *n* = 3. The data underlying this figure may be found in S12 Data. BACE1, β-site cleaving enzyme; BS, brain stem; Cer, cerebellum; FC, frontal cortex; HIF-1α, hypoxia-inducible factor; OC, occipital cortex; PC, parietal cortex; SIV, simian immunodeficincy virus; Thal, thalamus.
(TIF)

S3 Fig Brain region–specific expression of Aβ1–40 in SIV-infected macaques. Representative fluorescent photomicrographs showing differential expression of Aβ1–40 co-immunostained with GFAP^+^ astrocytes in the FC (A), PC (B), Hp (C), BG (D), and Cer (E) of saline- and SIV-infected macaques. *n* = 4. Scale bar, 10 μm. Aβ, amyloid beta; BG, basal ganglia; Cer, cerebellum; FC, frontal cortex; GFAP, glial fibrillary acidic protein; Hp, hippocampus; PC, parietal cortex; SIV, simian immunodeficincy virus.
(TIF)

S4 Fig Brain region–specific expression of p-Tau in SIV-infected macaques. Representative fluorescent photomicrographs showing differential expression of p-Tau co-immunostained with MAP2^+^ neurons in the FC (A), PC (B), Hp (C), BG (D), and Cer (E) of saline- and SIV-infected macaques. *n* = 4. Scale bar, 10 μm. BG, basal ganglia; Cer, cerebellum; FC, frontal cortex; Hp, hippocampus; MAP2, microtublule associated protein 2; PC, parietal cortex; SIV, simian immunodeficincy virus.
(TIF)

S5 Fig Expression of BACE1-AS RNA in various brain regions of SIV-infected macaques by in situ hybridization. Representative FISH and immunofluorescence photomicrographs showing differential expression of BACE1-AS RNA co-immunostained with GFAP^+^ astrocytes or MAP2^+^ neurons in the FC (A) and Hp (B) of saline- and SIV-infected macaques. Scale bar, 10 μm. *n* = 4. Arrows indicate GFAP-positive astrocytes colocalized with BACE1-AS RNA. BACE1-AS, BACE1‐antisense transcript; FC, frontal cortex; FISH, fluorescent insitu hybridization; GFAP, glial fibrillary acidic protein; Hp, hippocampus; MAP2, microtublule associated protein-2; SIV, simian immunodeficiency virus.
(TIF)

S6 Fig Expression of APP RNA in various brain regions of SIV-infected macaques by in situ hybridization. Representative FISH and IF photomicrographs in the FC showing differential expression of APP RNA co-immunostained with GFAP^+^ astrocytes or MAP2^+^ neurons (A) and quantative analysis of percent of GFAP^+^ astrocytes or MAP2^+^ neurons colocalized with APP RNA in the FC (C). Representative FISH and IF photomicrographs in the Hp showing differential expression of APP RNA co-immunostained with GFAP^+^ astrocytes or MAP2^+^ neurons (B) and quantative analysis of percent of GFAP^+^ astrocytes or MAP2^+^ neurons colocalized with APP RNA in the Hp (D). Scale bar, 10 μm. *n* = 4. Data are presented as mean ± SEM; *n* = 3. Student *t* test was used to determine the statistical significance between two groups: *P < 0.05 versus control. The data underlying this figure may be found in S13 Data. APP, amyloid precursor protein; FC, frontal cortex; FISH, fluorescent insitu hybridization; GFAP, glial fibrillary acidic protein; Hp, hippocampus; MAP2, microtubule associated protein 2; SIV, simian immunodeficiency virus.
(TIF)

S7 Fig HIV-1 Tat–mediated nuclear translocation of HIF-1α in HIF-1α–overexpressing HPAs. (A) Representative western blot showing expression of HIF-1α in endogenous and overexpressing HPA in the presence or absence of HIV-1 Tat (3.57 nM). (B) Representative fluorescent photomicrographs showing increased expression of HIF-1α in HIF-1α–overexpressing HPAs in the presence or absence of HIV-1 Tat (3.57 nM; 30 minutes). Scale bar, 10 μm. (C) RNA-seq data representing heatmaps for dysregulated genes—APP, HIF-1α, BACE1, BACE1-AS, and GFAP in control or Tat-exposed HPAs. (D) RNA-seq data of the HIF-1α–bound RNA complexes (RIP assay). Data are presented as mean ± SEM; *n* = 6. One-way ANOVA followed by Bonferroni post hoc test was used to determine the statistical significance between multiple groups: *P < 0.05 versus control. The data underlying this figure may be found in S14 Data. APP, amyloid precursor protein; BACE1, β-site cleaving enzyme; BACE1-AS, BACE1‐antisense transcript; GFAP, glial fibrillary acidic protein; HPA, human primary astrocyte; HIF-1α, hypoxia-inducible factor; RIP, RNA immunoprecipitation; RNA-seq, RNA-sequencing; Tat, transactivator of transcription
(TIF)

S8 Fig HIV-1 Tat–mediated regulation of PHD-2 in modulating astrocytic amyloidosis. (A) qPCR analysis demonstrating expression of PHD-2, HIF-1α, BACE1-AS, BACE1, and APP mRNAs in HPAs transfected with either PHD-2/scrambled siRNA in the presence or absence of HIV-1 Tat (3.57 nM; 24 hours), (B) Representative western blots showing the expression of PHD-2, HIF-1α, BACE1, APP, and Aβ mOC64 proteins in HPAs transfected with either PHD-2 or scrambled siRNA, (C) qPCR analysis demonstrating expression of PHD-2, HIF-1α, BACE1-AS, BACE1, and APP mRNAs in HPAs transfected with PHD-2 overexpressing plasmid in the presence or absence of HIV-1 Tat (3.57 nM; 24 hours), (D) representative western blots showing the expression of PHD-2, HIF-1α, BACE1, APP, and Aβ mOC64 proteins in HPAs transfected with PHD-2 overexpressing plasmid in the presence or absence of HIV-1 Tat (3.57 nM; 24 hours). Data are presented as mean ± SEM; *n* = 6. One-way ANOVA followed by Bonferroni post hoc test was used to determine the statistical significance: *P < 0.05 versus control, ^#^*P* < 0.05 versus Tat. The data underlying this figure may be found in S15 Data. APP, amyloid precursor protein; Aβ, amyloid beta; BACE1, β-site cleaving enzyme; BACE1-AS, BACE1‐antisense transcript; HIF-1α, hypoxia-inducible factor; HPA, human primary astrocyte; qPCR, quantitative polymerase chain reaction; PHD-2, prolyl hydroxylase 2; siRNA, small interfering RNA; Tat, transactivator of transcription.
(TIF)

S9 Fig Phagocytic activity in HIV-1 Tat–exposed HPAs. Representative immunocytochemistry images showing the phagocytic potential of GFAP^+^ HPAs in control and Tat–exposed cells (3.57 nM; 24 hours). Scale bar, 10 μm. *n* = 6. GFAP, glial fibrillary acidic protein; HPA, human primary astrocyte; Tat, transactivator of transcription.
(TIF)

S10 Fig HIV-1 Tat–mediated amyloidosis in human neurons. qPCR analysis showing expression of APP, BACE1, BACE1-AS, HIF-1, and PHD-2 mRNAs in human primary neurons exposed to HIV-1 Tat (3.57 nM, 24 hours). GAPDH was used as an internal control for mRNA expression (A). qPCR analysis showing expression of APP, BACE1, BACE1-AS, HIF-1, and PHD-2 mRNAs in SHSY-5Y cells exposed to HIV-1 Tat (3.57 nM, 24 hours). GAPDH was used as an internal control for mRNA expression (B). Western blot analysis showing expression of HIF-1α, BACE 1, APP, Aβ mOC64, and PHD-2 proteins in SHSY-5Y cells exposed to HIV-1 Tat (3.57 nM, 24 hours). β-actin was used as an internal control (C). qPCR analysis showing expression of APP, BACE1, BACE1-AS, HIF-1α, and PHD-2 mRNAs in SHSY-5Y cells exposed to HIV-1 Tat (3.57 nM, 48 hours). GAPDH was used as an internal control for mRNA expression (D). Western blot analysis showing expression of HIF-1α, BACE 1, APP, Aβ mOC64, and PHD-2 proteins in SHSY-5Y cells exposed to HIV-1 Tat (3.57 nM, 48 hours). β-actin was used as an internal control (E). Data are presented as mean ± SEM; *n* = 6. Student *t* test was used to determine the statistical significance: *P < 0.05 versus control. The data underlying this figure may be found in S16 Data. APP, amyloid precursor protein; Aβ, amyloid beta; BACE1, β-site cleaving enzyme; BACE1-AS, BACE1‐antisense transcript; GAPDH, glyceraldehyde 3-phosphate dehydrogenase; HIF-1α, hypoxia inducible factor-1α; PHD-2, prolyl hydroxylase 2; qPCR, quantitative polymerase chain reaction; SHSY-5Y, neuroblastoma cell line; Tat, transactivator of transcription
(TIF)

S1 Raw Images (PDF)

S1 Methods (DOCX)

S1 Data (XLSX)

S2 Data (XLSX)

S3 Data (XLSX)

S4 Data (XLSX)

S5 Data (XLSX)

S6 Data (XLSX)

S7 Data (XLSX)

S8 Data (XLSX)

S9 Data (XLSX)

S10 Data (XLSX)

S11 Data (XLSX)

S12 Data (XLSX)

S13 Data (XLSX)

S14 Data (XLSX)

S15 Data (XLSX)

S16 Data (XLSX)

## Abbreviations

AD: Alzheimer Disease
AIF1: allograft inflammatory factor 1
APOE ε4: apolipoprotein E4
APP: amyloid precursor protein
Aβ: amyloid beta
BACE1: β-site cleaving enzyme
BACE1-AS: BACE1‐antisense transcript
BG: basal ganglia
BL: biotin-labeled
BS: brain stem
cART: combined antiretroviral therapy
Cer: cerebellum
ChIP: chromatin immunoprecipitation
CNS: central nervous system
CNTN: California NeuroAIDS Tissue Network
CSF: cerebrospinal fluid
DHHS: Department of Health and Human Services
EMSA: electrophorectic mobility shift assay
ERK: extracellular-signal-regulated kinase
FC: frontal cortex
FFPE: formalin-fixed paraffin-embedded
FIH: Factor Inhibiting HIF
GAPDH: glyceraldehyde 3-phosphate dehydrogenase
GFAP: glial fibrillary acidic protein
HAND: HIV-associated neurocognitive disorder
HIF-1α: hypoxia-inducible factor
Hp: hippocampus
HPA: human primary astrocyte
IgG: immunoglobulin G
IRB: institutional review board
ISMMS: Icahn School of Medicine at Mount Sinai
JAK-STAT: Janus kinase/signal transducers and activators of transcription
lncRNA: long noncoding RNA
MAPK: mitogen activated protein kinase
MAP2: microtublule associated protein 2
MCBP: mean cortical binding potential
MCP-1: monocyte chemoattractant protein-1
MHBB: Manhattan HIV Brain Bank
mTOR: mammalian target of rapamycin
NNTC: NeuroAIDS Tissue Consortium
OC: occipital cortex
PC: parietal cortex
PHD-2: prolyl hydroxylase 2
PI3K: phosphatidylinositol 3-kinase
PS: positive control
qPCR: quantative polymerase chain reaction
RIP: RNA immunoprecipitation
RNA FISH: RNA fluorescent insitu hybridization
RNA-seq: RNA sequencing
siRNA: small interfering RNA
SIV: simian immunodeficiency virus
Tat: transactivator of transcription
Thal: thalamus
TNRC: Texas NeuroAIDS Research Center
UCSD: University of California, San Diego
WSB1: WD repeat and SOCS box-containing protein 1
