# Supplementary material for: HIV-1 Tat-mediated astrocytic amyloidosis involves the HIF-1α/lncRNA BACE1-AS axis
Source: PLoS Biol. 2020 May 26;18(5):e3000660. doi: 10.1371/journal.pbio.3000660 (PMC7274476; doi:10.1371/journal.pbio.3000660)
Supplement: S1 Text — Aβ, amyloid beta; GFAP, glial fibrillary acidic protein; SIV, simian immunodeficincy virus. (DOCX) [file pbio.3000660.s001.docx]

**Expression of Aβ 1-42 and co-localization with GFAP in brain regions of SIV-infected macaques:** The brain sections from different brain regions (FC, PC, Hippocampus (Hippo), Basal Ganglia (BG), Cer and BS, were co-immunostained for the expression of Aβ1-42 in GFAP+ astrocytes. As shown in S1 Fig, there was increased co localization of Aβ1-42 in GFAP+ astrocytes in all of the brain regions of SIV-infected macaques compared with those of the saline group (S1A, 1B, 1C, 1D, 1E and 1F Fig). Intriguingly however, within the various brain regions of the SIV+ macaques there appeared to be differential expression of the toxic Aβ1-42 protein. More intense staining of Aβ1-42 appeared to be present in the FC, Hippo and the BG compared to other regions tested.
