# Supplementary material for: HIV-1 Tat-mediated astrocytic amyloidosis involves the HIF-1α/lncRNA BACE1-AS axis
Source: PLoS Biol. 2020 May 26;18(5):e3000660. doi: 10.1371/journal.pbio.3000660 (PMC7274476; doi:10.1371/journal.pbio.3000660)
Supplement: S2 Text — BACE1, β-site cleaving enzyme; HIF-1α, hypoxia-inducible factor; SIV, simian immunodeficincy virus. (DOCX) [file pbio.3000660.s002.docx]

**Expression of HIF-1α and BACE1 in SIV-infected macaques:** HIF-1α and BACE1 were differentially upregulated in the various brain regions of SIV infected macaques; with only the FC, PC, Cer for HIF-1α and FC, Cer for BACE 1 exhibiting significant upregulation *(*P*<0.05) in the SIV+ group compared with the saline injected macaques (S2A and 2B Fig).
