# Supplementary material for: HIV-1 Tat-mediated astrocytic amyloidosis involves the HIF-1α/lncRNA BACE1-AS axis
Source: PLoS Biol. 2020 May 26;18(5):e3000660. doi: 10.1371/journal.pbio.3000660 (PMC7274476; doi:10.1371/journal.pbio.3000660)
Supplement: S3 Text — Aβ, amyloid beta; SIV, simian immunodeficincy virus. (DOCX) [file pbio.3000660.s003.docx]

**Differential expression of Aβ1-40 in the brains of SIV-infected macaques:** In addition to Aβ1-42, upregulation of Aβ1-40 variety was also observed and found to be differentially expressed in various brain regions of SIV-infected macaques, specifically in the astrocytes (S3 Fig).
