# Supplementary material for: HIV-1 Tat-mediated astrocytic amyloidosis involves the HIF-1α/lncRNA BACE1-AS axis
Source: PLoS Biol. 2020 May 26;18(5):e3000660. doi: 10.1371/journal.pbio.3000660 (PMC7274476; doi:10.1371/journal.pbio.3000660)
Supplement: S4 Text — SIV, simian immunodeficincy virus. (DOCX) [file pbio.3000660.s004.docx]

**Differential expression of p-Tau in the brains of SIV-infected macaques:** In addition to amyloid deposition by astrocytes, we also detected region specific upregulation of neuronal p-Tau in the brains of SIV-infected macaques (S4 Fig) compared with the uninfected controls.
