# Supplementary material for: HIV-1 Tat-mediated astrocytic amyloidosis involves the HIF-1α/lncRNA BACE1-AS axis
Source: PLoS Biol. 2020 May 26;18(5):e3000660. doi: 10.1371/journal.pbio.3000660 (PMC7274476; doi:10.1371/journal.pbio.3000660)
Supplement: S5 Text — BACE1-AS, BACE1‐antisense transcript; RNA FISH, RNA fluorescent insitu hybridization; SIV, simian immunodeficiency virus. (DOCX) [file pbio.3000660.s005.docx]

***In situ* hybridization (RNA FISH) of BACE1-AS RNA in the brains of SIV-infected macaques:** Archival FC and Hippo regions of SIV-infected macaques demonstrated increased upregulation of BACE1-AS RNA in the GFAP+ astrocytes compared with the expression in the same brain regions of the uninfected controls (S5A and 5B Fig).
