# Supplementary material for: HIV-1 Tat-mediated astrocytic amyloidosis involves the HIF-1α/lncRNA BACE1-AS axis
Source: PLoS Biol. 2020 May 26;18(5):e3000660. doi: 10.1371/journal.pbio.3000660 (PMC7274476; doi:10.1371/journal.pbio.3000660)
Supplement: S6 Text — APP, amyloid precursor protein; RNA FISH, RNA fluorescent insitu hybridization; SIV, simian immunodeficiency virus (DOCX) [file pbio.3000660.s006.docx]

***In situ* hybridization (RNA FISH) of APP RNA in the brains of SIV-infected macaques:** Archival FC and Hippo regions of SIV-infected macaques demonstrated increased upregulation of APP RNA in the GFAP+ astrocytes as well as MAP^+^ neurons compared with the expression in the same brain regions of the saline (S6A and 6B Fig). Quantitative analysis showed significant *(p<0.05) co localization of astrocytes and neurons with APP RNA both in the frontal cortex and hippocampus in SIV compared to saline group (S6C and 6D Fig).
