# Supplementary material for: HIV-1 Tat-mediated astrocytic amyloidosis involves the HIF-1α/lncRNA BACE1-AS axis
Source: PLoS Biol. 2020 May 26;18(5):e3000660. doi: 10.1371/journal.pbio.3000660 (PMC7274476; doi:10.1371/journal.pbio.3000660)
Supplement: S7 Text — HIF-1α, hypoxia-inducible factor; HPA, human primary astrocyte; Tat, transactivator of transcription (DOCX) [file pbio.3000660.s007.docx]

**Increased Tat-mediated nuclear translocation of HIF-1α in HIF-1α overexpressing HPA:** As shown in S7A Fig there was significant *(*P*<0.05) upregulation of HIF-1α in HIF-1α overexpressing HPAs in the presence or absence of Tat compared to that of control. Furthermore, as shown in S7B Fig, in the presence of Tat there was increased nuclear translocation of HIF-1α in HIF-1α overexpressing HPAs, as evidenced by immunofluorescence. Additionally, RNA sequencing of control and Tat exposed astrocytes showed that the mRNA expression of APP, HIF-1α, BACE1, BACE1-AS and GFAP were significantly upregulated *(p<0.05) in Tat treated HPA compared to that of control (S7C Fig).
