# Supplementary material for: HIV-1 Tat-mediated astrocytic amyloidosis involves the HIF-1α/lncRNA BACE1-AS axis
Source: PLoS Biol. 2020 May 26;18(5):e3000660. doi: 10.1371/journal.pbio.3000660 (PMC7274476; doi:10.1371/journal.pbio.3000660)
Supplement: S8 Text — PHD-2, prolyl hydroxylase 2; Tat, transactivator of transcription (DOCX) [file pbio.3000660.s008.docx]

**PHD-2 in HIV-1 Tat-mediated amyloidosis**: As shown in S8A and 8B Fig, exposure of scrambled siRNA transfected HPAs to HIV-1 Tat resulted in significantly *(p<0.05) increased expression of HIF-1α, APP, BACE 1, BACE1-AS and AβmOC64 with concomitantly decreased expression of PHD-2 compared to controls. Furthermore, silencing of PHD-2 resulted in significant increase *(p< 0.05) in expression of HIF-1, APP, BACE 1 mRNAs and BACE1-AS by real-time RT-PCR. Western blot showed significant upregulation *(p< 0.05) of HIF-1α, APP, AβmOC64 and BACE 1 proteins (S8B Fig.), with or without exposure to Tat compared to scrambled-siRNA transfected astrocytes (control).

To further validate the role PHD-2 in amyloidosis, HPAs were transfected with PHD-2 overexpressing plasmid. As shown in S8C Fig, overexpression of PHD-2, resulted in significant decrease #(p< 0.05) in expression of HIF-1α, APP, BACE 1 mRNAs and BACE1-AS by real-time RT-PCR. Western blotting showed significant downregulation #(p< 0.05) in expression of HIF-1α, APP, AβmOC64 and BACE 1 proteins (S8D Fig), with or without Tat exposure compared to Tat-exposed astrocytes, thus showing the intriguing role of PHD-2 in HIV-1 Tat mediated astrocytic amyloidosis.
