# Supplementary material for: HIV-1 Tat-mediated astrocytic amyloidosis involves the HIF-1α/lncRNA BACE1-AS axis
Source: PLoS Biol. 2020 May 26;18(5):e3000660. doi: 10.1371/journal.pbio.3000660 (PMC7274476; doi:10.1371/journal.pbio.3000660)
Supplement: S9 Text — HPA, human primary astrocyte; Tat, transactivator of transcription (DOCX) [file pbio.3000660.s009.docx]

**Phagocytic activity of HIV-1 Tat exposed HPAs:** Phagocytosis assay showed that HIV-1 Tat exposed HPAs in culture did not phagocytose any Fitc+ beads confirming no alteration in the phagocytic activity of astrocytes in presence or absence of Tat (S9 Fig). So, this depicts that the Aβ 42 observed in GFAP^+^ astrocytes is not due to phagocytosis of external amyloids, but are produced by the astrocytes.
