# Supplementary material for: HIV-1 Tat-mediated astrocytic amyloidosis involves the HIF-1α/lncRNA BACE1-AS axis
Source: PLoS Biol. 2020 May 26;18(5):e3000660. doi: 10.1371/journal.pbio.3000660 (PMC7274476; doi:10.1371/journal.pbio.3000660)
Supplement: S10 Text — Tat, transactivator of transcription (DOCX) [file pbio.3000660.s010.docx]

**Amyloidosis in HIV-1 Tat exposed neurons:** Exposure of human primary neurons to HIV-1 Tat (3.57 nM) for 24h failed to demonstrate an increase in HIF-1α, BACE1, BACE1-AS, APP mRNAs compared with control cells (S10A Fig). These findings were also validated in neuroblastoma cells (SHY-5Y) for both the protein & mRNA expression (S10B and 10C Fig). At 48 hrs post HIV-1 Tat exposure there was a significant increase *(p<0.05) in APP, BACE1 mRNAs and APP, Aβm0C64, BACE1 proteins, with no change in mRNA or protein expression of HIF-1α and PHD-2 (S10D and 10E Fig) indicating thus that HIV-1 Tat mediated neuronal amyloidosis is HIF-1α independent.
