# Supplementary figures and images for: HIV-1 Tat-mediated astrocytic amyloidosis involves the HIF-1α/lncRNA BACE1-AS axis
Source: PLoS Biol. 2020 May 26;18(5):e3000660. doi: 10.1371/journal.pbio.3000660 (PMC7274476; doi:10.1371/journal.pbio.3000660)

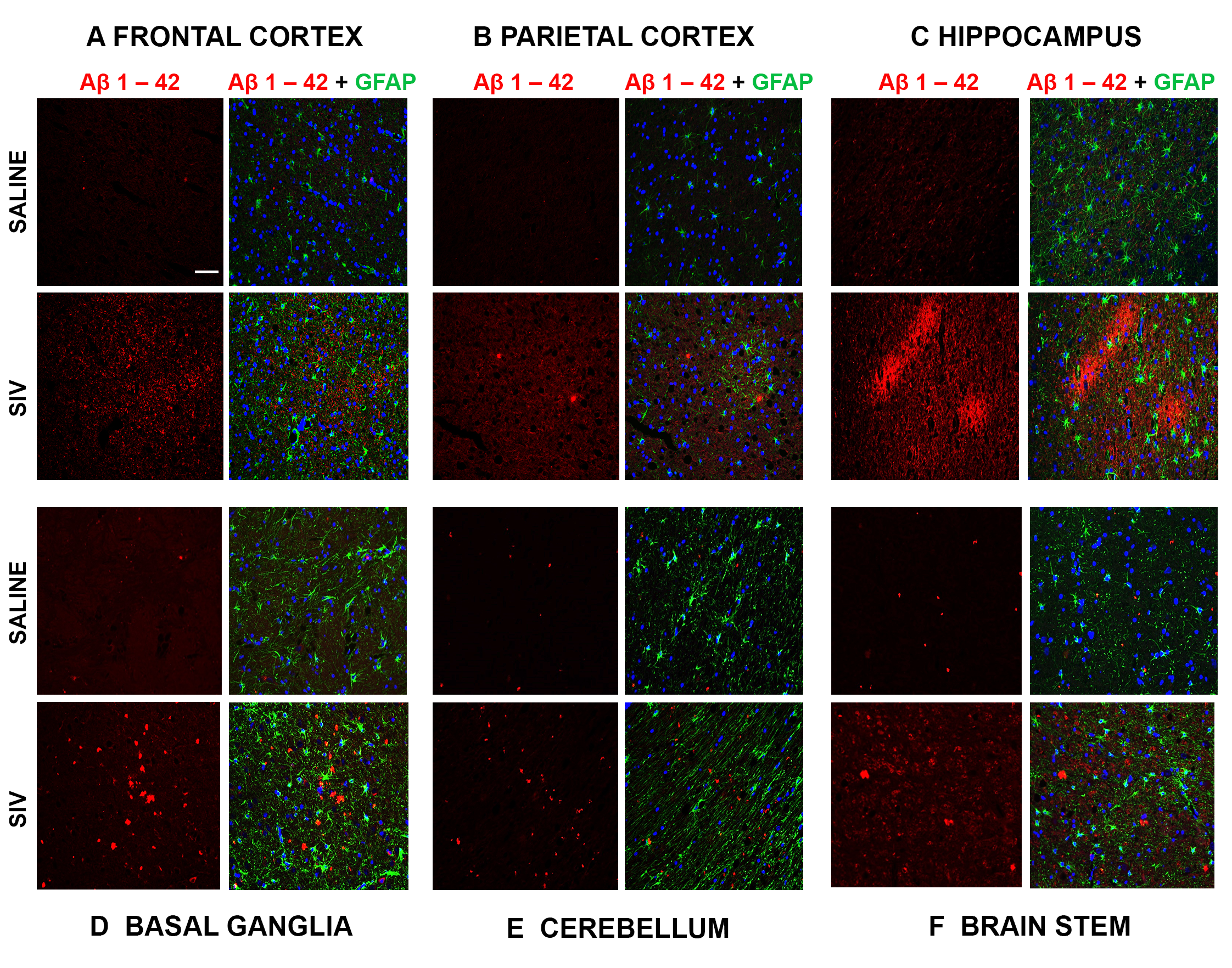

Supplement: S1 Fig — Representative fluorescent photomicrographs showing differential expression of Aβ 1–42 in GFAP+ astrocytes in the different brain regions of saline and SIV+ macaques. Scale bar, 10 μm. Saline, n = 4; SIV, n = 3. Aβ, amyloid beta; GFAP, glial fibrillary acidic protein; SIV, simian immunodeficincy virus. (TIF) [file pbio.3000660.s011.tif]

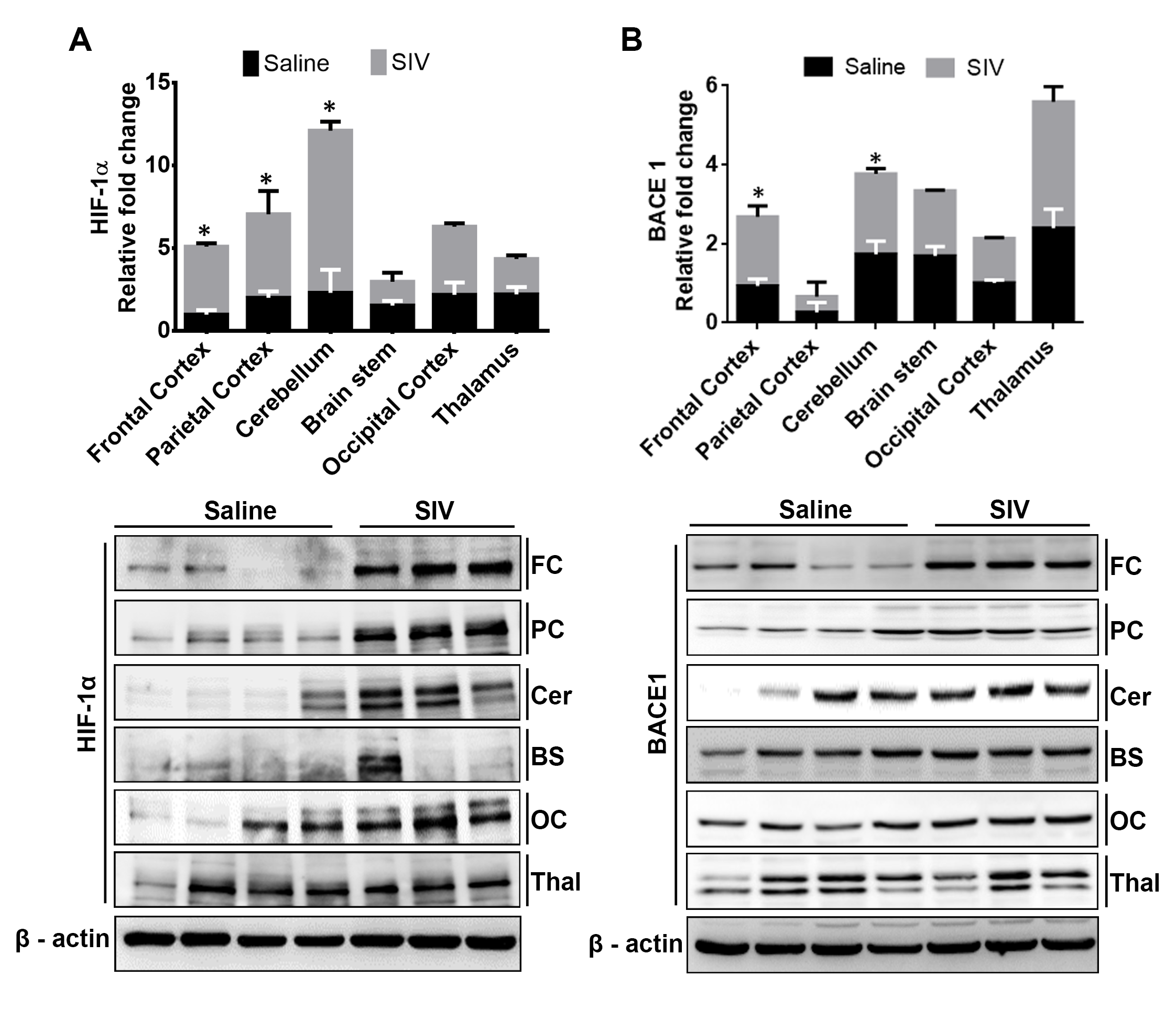

Supplement: S2 Fig — (A) Representative western blots showing the expression of HIF-1α in different brain regions—FC, PC, Cer, BS, OC, and Thal of saline- and SIV-infected macaques. (B) Representative western blots showing the expression of BACE1 in different brain regions—FC, PC, Cer, BS, OC, and Thal of saline- and SIV-infected macaques. β-actin was used as an internal control. n = 6. Data are presented as mean ± SEM; saline, n = 4; SIV, n = 3. The data underlying this figure may be found in S12 Data. BACE1, β-site cleaving enzyme; BS, brain stem; Cer, cerebellum; FC, frontal cortex; HIF-1α, hypoxia-inducible factor; OC, occipital cortex; PC, parietal cortex; SIV, simian immunodeficincy virus; Thal, thalamus. (TIF) [file pbio.3000660.s012.tif]

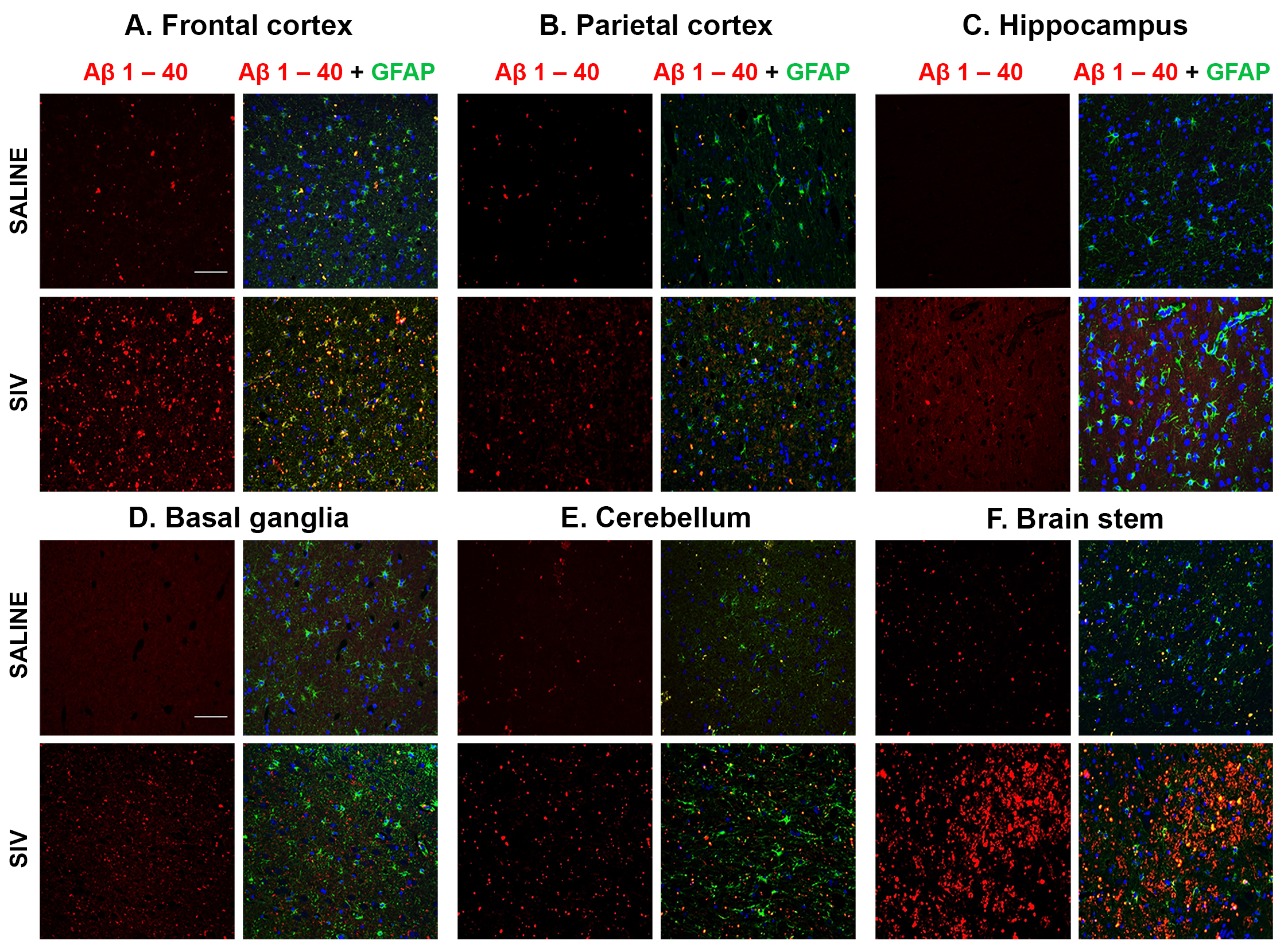

Supplement: S3 Fig — Representative fluorescent photomicrographs showing differential expression of Aβ1–40 co-immunostained with GFAP+ astrocytes in the FC (A), PC (B), Hp (C), BG (D), and Cer (E) of saline- and SIV-infected macaques. n = 4. Scale bar, 10 μm. Aβ, amyloid beta; BG, basal ganglia; Cer, cerebellum; FC, frontal cortex; GFAP, glial fibrillary acidic protein; Hp, hippocampus; PC, parietal cortex; SIV, simian immunodeficincy virus. (TIF) [file pbio.3000660.s013.tif]

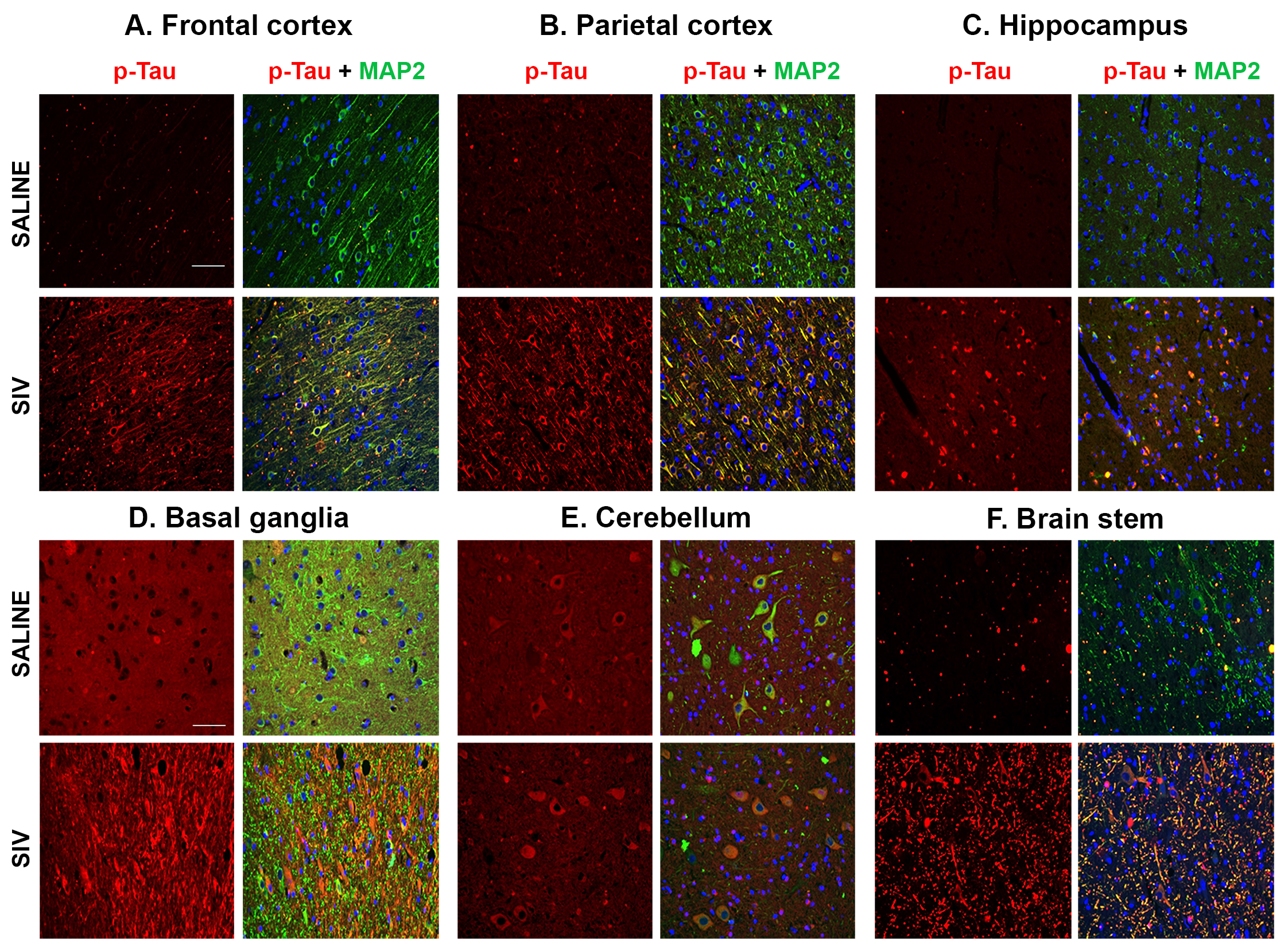

Supplement: S4 Fig — Representative fluorescent photomicrographs showing differential expression of p-Tau co-immunostained with MAP2+ neurons in the FC (A), PC (B), Hp (C), BG (D), and Cer (E) of saline- and SIV-infected macaques. n = 4. Scale bar, 10 μm. BG, basal ganglia; Cer, cerebellum; FC, frontal cortex; Hp, hippocampus; MAP2, microtublule associated protein 2; PC, parietal cortex; SIV, simian immunodeficincy virus. (TIF) [file pbio.3000660.s014.tif]

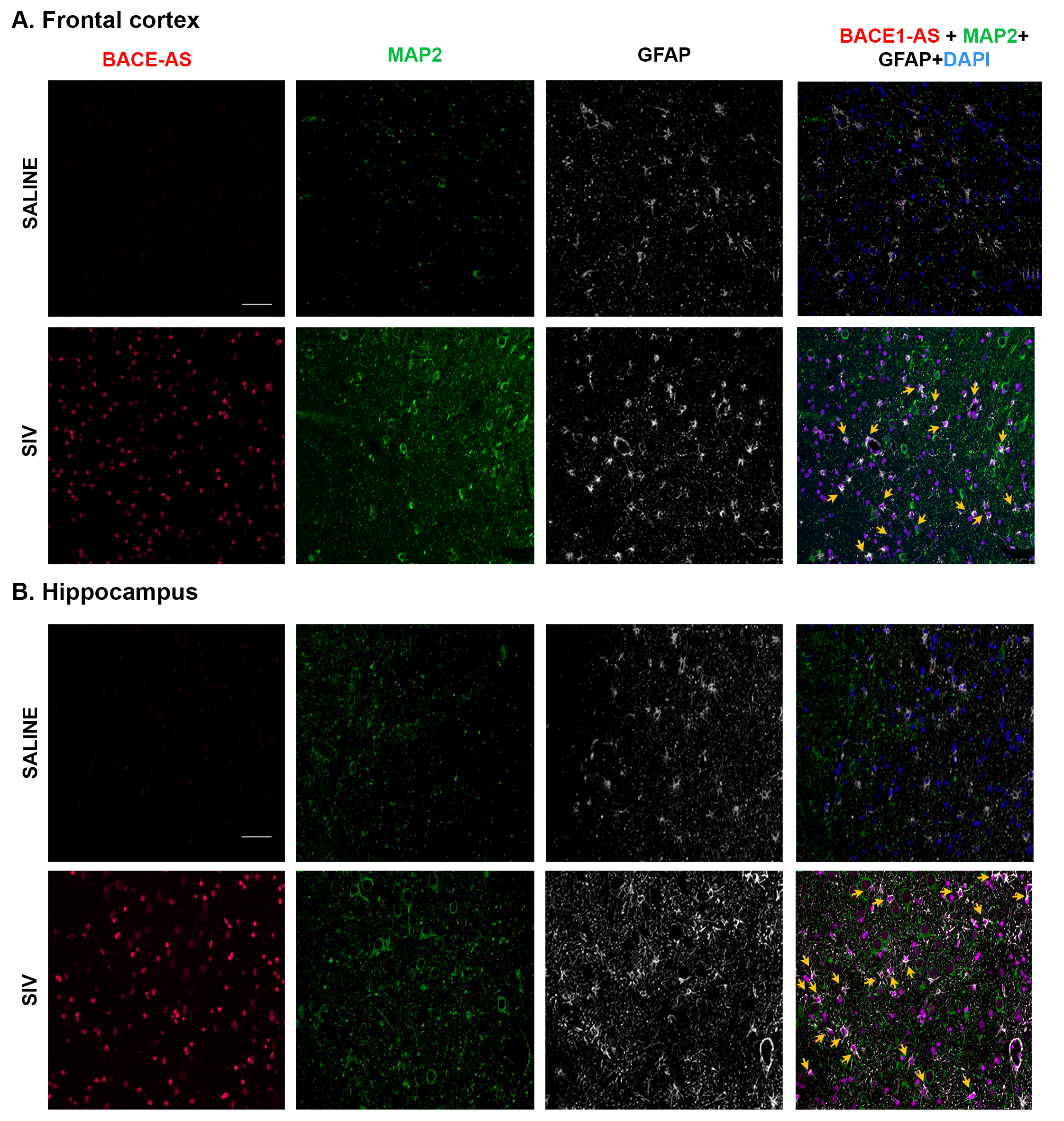

Supplement: S5 Fig — Representative FISH and immunofluorescence photomicrographs showing differential expression of BACE1-AS RNA co-immunostained with GFAP+ astrocytes or MAP2+ neurons in the FC (A) and Hp (B) of saline- and SIV-infected macaques. Scale bar, 10 μm. n = 4. Arrows indicate GFAP-positive astrocytes colocalized with BACE1-AS RNA. BACE1-AS, BACE1‐antisense transcript; FC, frontal cortex; FISH, fluorescent insitu hybridization; GFAP, glial fibrillary acidic protein; Hp, hippocampus; MAP2, microtublule associated protein-2; SIV, simian immunodeficiency virus. (TIF) [file pbio.3000660.s015.tif]

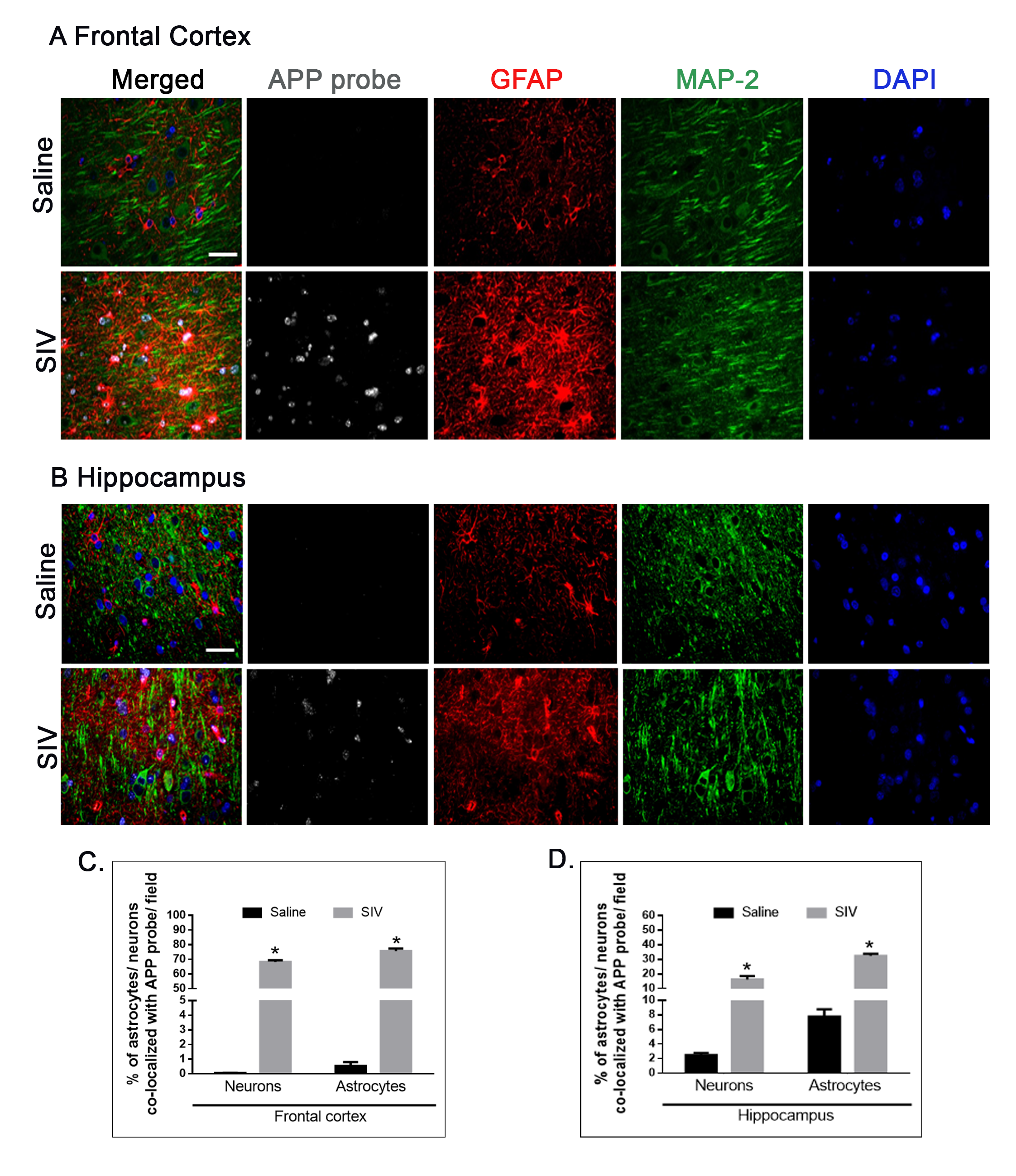

Supplement: S6 Fig — Representative FISH and IF photomicrographs in the FC showing differential expression of APP RNA co-immunostained with GFAP+ astrocytes or MAP2+ neurons (A) and quantative analysis of percent of GFAP+ astrocytes or MAP2+ neurons colocalized with APP RNA in the FC (C). Representative FISH and IF photomicrographs in the Hp showing differential expression of APP RNA co-immunostained with GFAP+ astrocytes or MAP2+ neurons (B) and quantative analysis of percent of GFAP+ astrocytes or MAP2+ neurons colocalized with APP RNA in the Hp (D). Scale bar, 10 μm. n = 4. Data are presented as mean ± SEM; n = 3. Student t test was used to determine the statistical significance between two groups: *P < 0.05 versus control. The data underlying this figure may be found in S13 Data. APP, amyloid precursor protein; FC, frontal cortex; FISH, fluorescent insitu hybridization; GFAP, glial fibrillary acidic protein; Hp, hippocampus; MAP2, microtubule associated protein 2; SIV, simian immunodeficiency virus. (TIF) [file pbio.3000660.s016.tif]

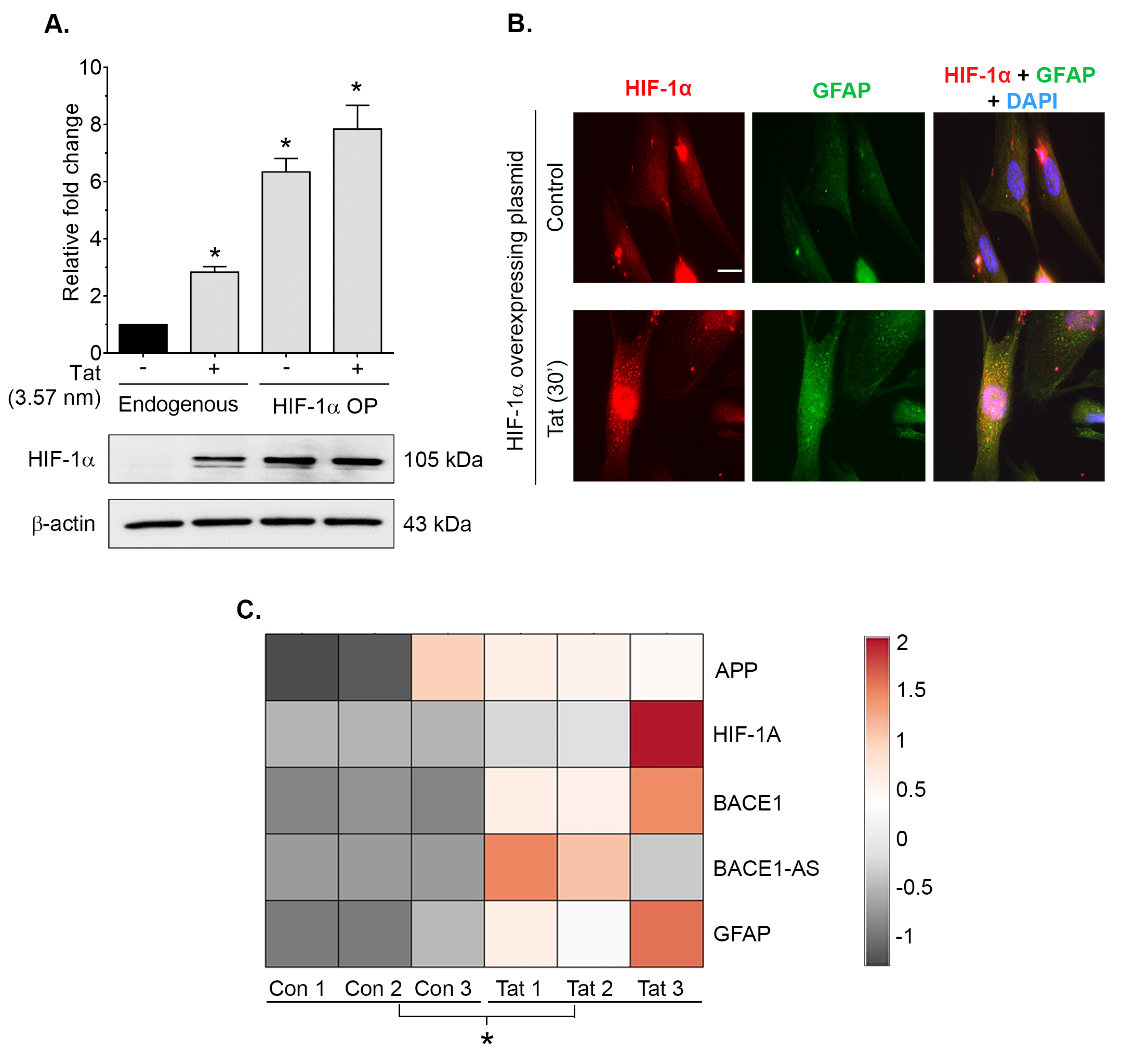

Supplement: S7 Fig — (A) Representative western blot showing expression of HIF-1α in endogenous and overexpressing HPA in the presence or absence of HIV-1 Tat (3.57 nM). (B) Representative fluorescent photomicrographs showing increased expression of HIF-1α in HIF-1α–overexpressing HPAs in the presence or absence of HIV-1 Tat (3.57 nM; 30 minutes). Scale bar, 10 μm. (C) RNA-seq data representing heatmaps for dysregulated genes—APP, HIF-1α, BACE1, BACE1-AS, and GFAP in control or Tat-exposed HPAs. (D) RNA-seq data of the HIF-1α–bound RNA complexes (RIP assay). Data are presented as mean ± SEM; n = 6. One-way ANOVA followed by Bonferroni post hoc test was used to determine the statistical significance between multiple groups: *P < 0.05 versus control. The data underlying this figure may be found in S14 Data. APP, amyloid precursor protein; BACE1, β-site cleaving enzyme; BACE1-AS, BACE1‐antisense transcript; GFAP, glial fibrillary acidic protein; HPA, human primary astrocyte; HIF-1α, hypoxia-inducible factor; RIP, RNA immunoprecipitation; RNA-seq, RNA-sequencing; Tat, transactivator of transcription (TIF) [file pbio.3000660.s017.tif]

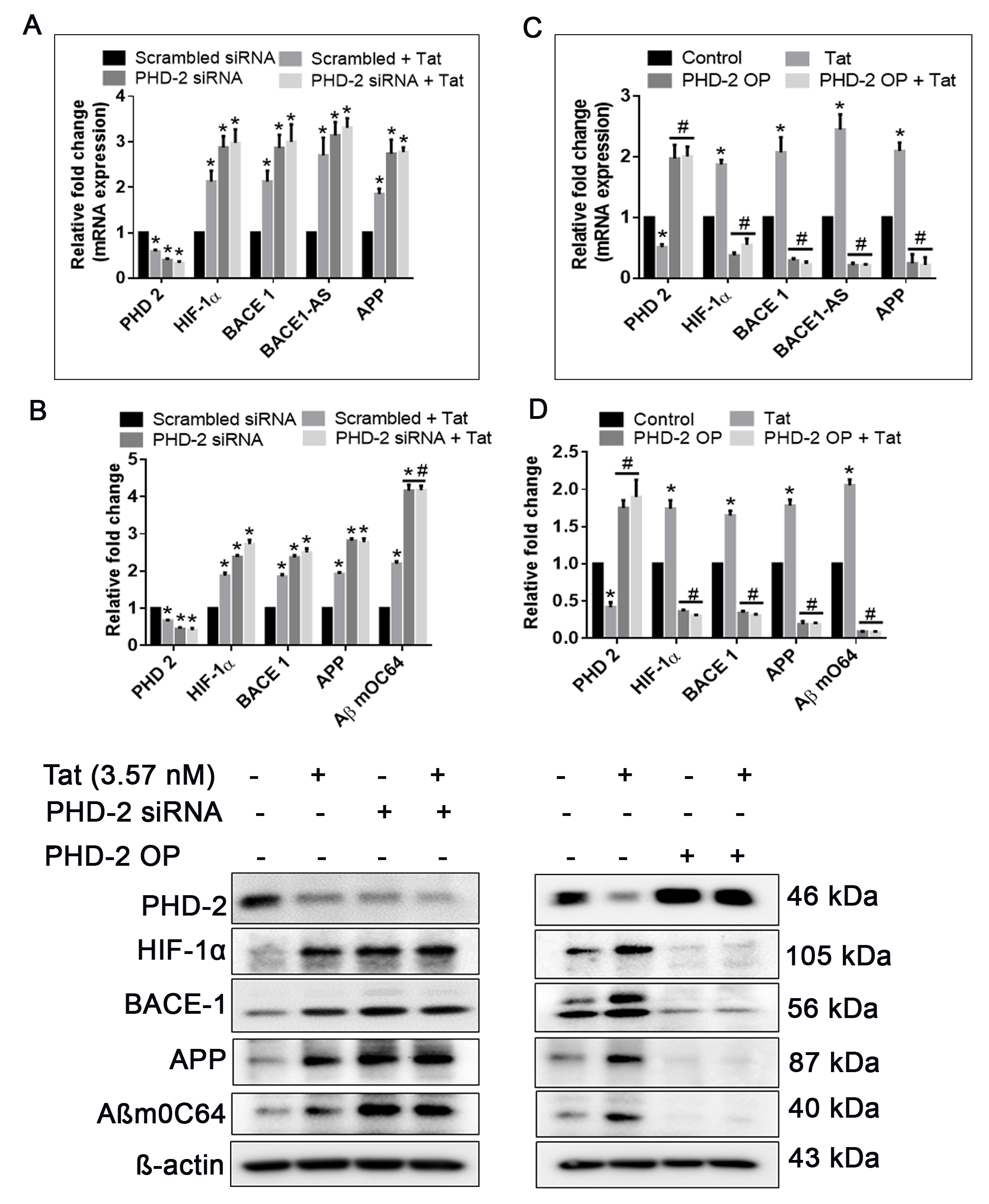

Supplement: S8 Fig — (A) qPCR analysis demonstrating expression of PHD-2, HIF-1α, BACE1-AS, BACE1, and APP mRNAs in HPAs transfected with either PHD-2/scrambled siRNA in the presence or absence of HIV-1 Tat (3.57 nM; 24 hours), (B) Representative western blots showing the expression of PHD-2, HIF-1α, BACE1, APP, and Aβ mOC64 proteins in HPAs transfected with either PHD-2 or scrambled siRNA, (C) qPCR analysis demonstrating expression of PHD-2, HIF-1α, BACE1-AS, BACE1, and APP mRNAs in HPAs transfected with PHD-2 overexpressing plasmid in the presence or absence of HIV-1 Tat (3.57 nM; 24 hours), (D) representative western blots showing the expression of PHD-2, HIF-1α, BACE1, APP, and Aβ mOC64 proteins in HPAs transfected with PHD-2 overexpressing plasmid in the presence or absence of HIV-1 Tat (3.57 nM; 24 hours). Data are presented as mean ± SEM; n = 6. One-way ANOVA followed by Bonferroni post hoc test was used to determine the statistical significance: *P < 0.05 versus control, #P < 0.05 versus Tat. The data underlying this figure may be found in S15 Data. APP, amyloid precursor protein; Aβ, amyloid beta; BACE1, β-site cleaving enzyme; BACE1-AS, BACE1‐antisense transcript; HIF-1α, hypoxia-inducible factor; HPA, human primary astrocyte; qPCR, quantitative polymerase chain reaction; PHD-2, prolyl hydroxylase 2; siRNA, small interfering RNA; Tat, transactivator of transcription. (TIF) [file pbio.3000660.s018.tif]

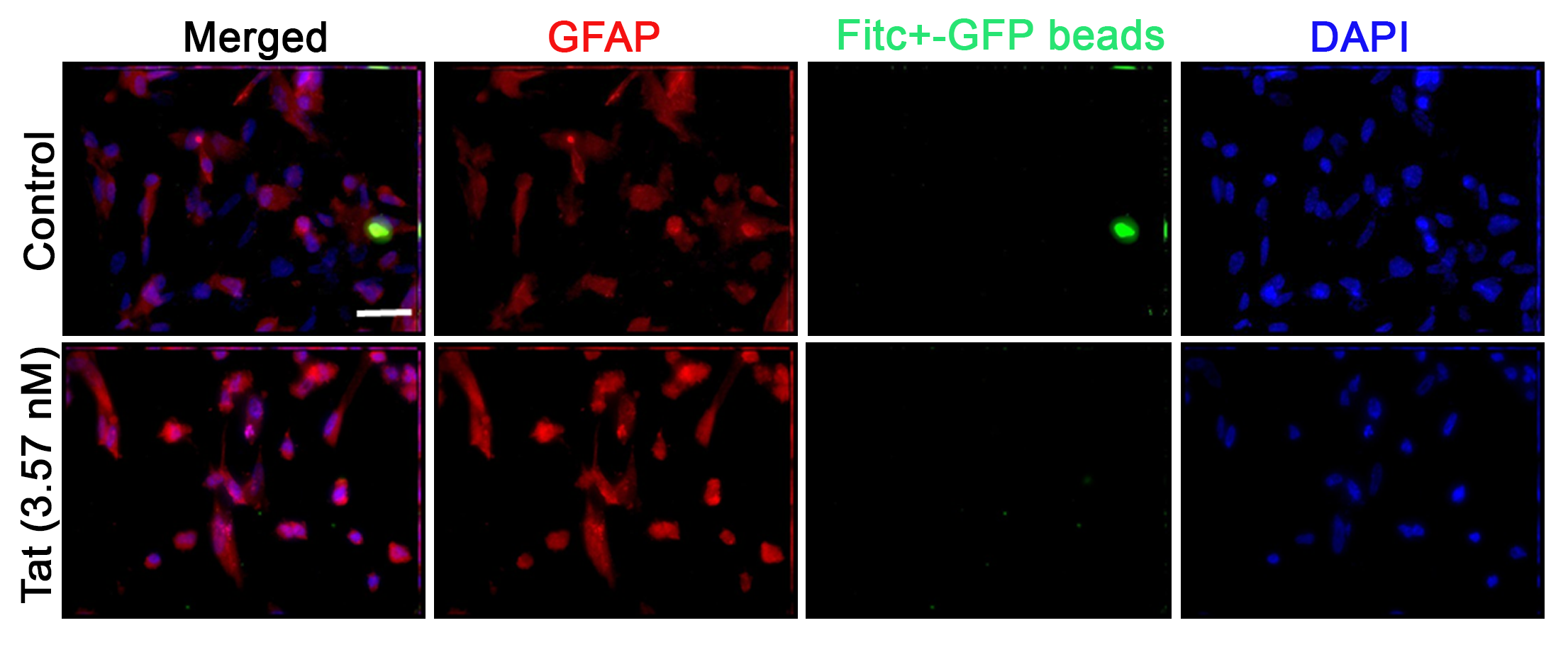

Supplement: S9 Fig — Representative immunocytochemistry images showing the phagocytic potential of GFAP+ HPAs in control and Tat–exposed cells (3.57 nM; 24 hours). Scale bar, 10 μm. n = 6. GFAP, glial fibrillary acidic protein; HPA, human primary astrocyte; Tat, transactivator of transcription. (TIF) [file pbio.3000660.s019.tif]

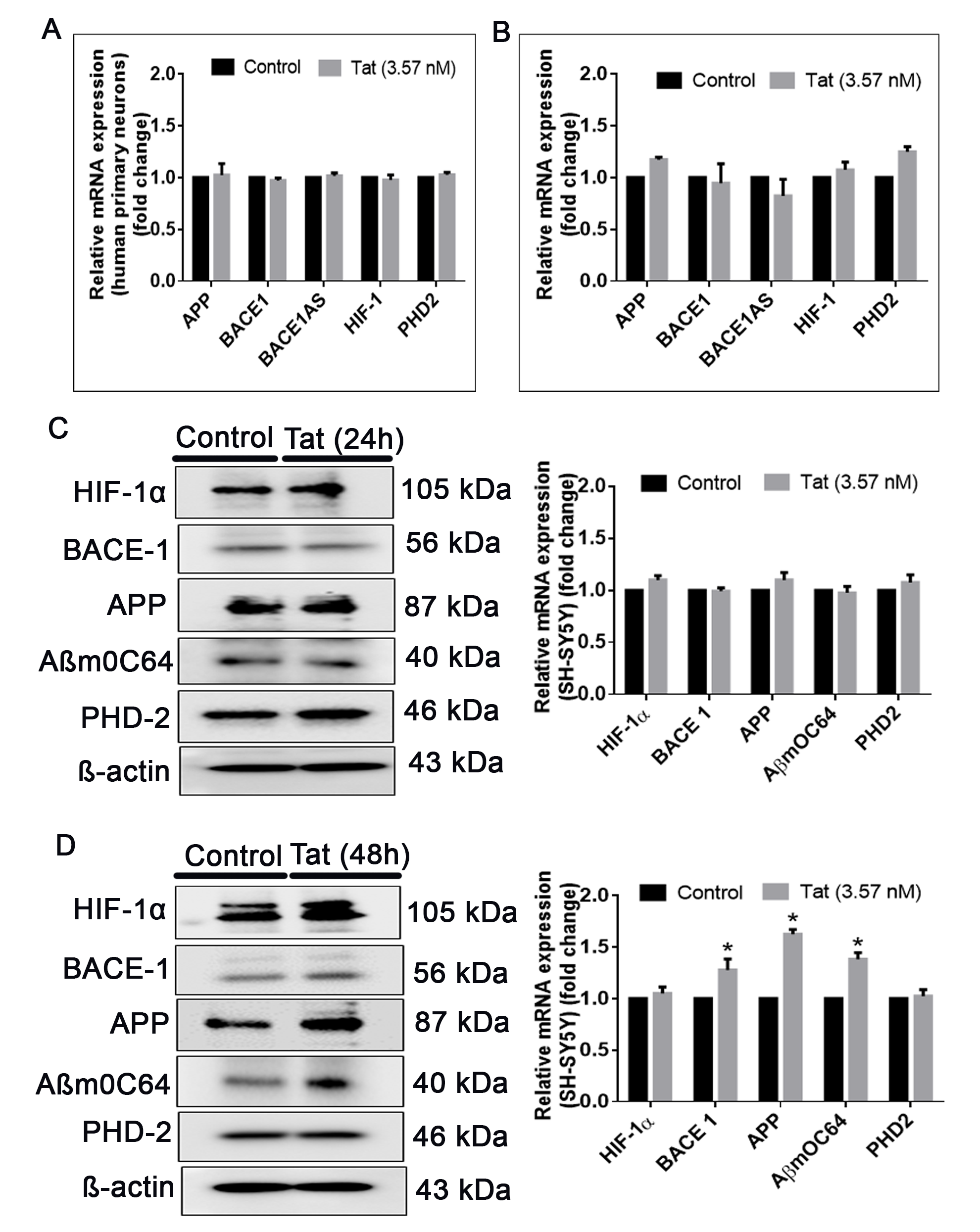

Supplement: S10 Fig — qPCR analysis showing expression of APP, BACE1, BACE1-AS, HIF-1, and PHD-2 mRNAs in human primary neurons exposed to HIV-1 Tat (3.57 nM, 24 hours). GAPDH was used as an internal control for mRNA expression (A). qPCR analysis showing expression of APP, BACE1, BACE1-AS, HIF-1, and PHD-2 mRNAs in SHSY-5Y cells exposed to HIV-1 Tat (3.57 nM, 24 hours). GAPDH was used as an internal control for mRNA expression (B). Western blot analysis showing expression of HIF-1α, BACE 1, APP, Aβ mOC64, and PHD-2 proteins in SHSY-5Y cells exposed to HIV-1 Tat (3.57 nM, 24 hours). β-actin was used as an internal control (C). qPCR analysis showing expression of APP, BACE1, BACE1-AS, HIF-1α, and PHD-2 mRNAs in SHSY-5Y cells exposed to HIV-1 Tat (3.57 nM, 48 hours). GAPDH was used as an internal control for mRNA expression (D). Western blot analysis showing expression of HIF-1α, BACE 1, APP, Aβ mOC64, and PHD-2 proteins in SHSY-5Y cells exposed to HIV-1 Tat (3.57 nM, 48 hours). β-actin was used as an internal control (E). Data are presented as mean ± SEM; n = 6. Student t test was used to determine the statistical significance: *P < 0.05 versus control. The data underlying this figure may be found in S16 Data. APP, amyloid precursor protein; Aβ, amyloid beta; BACE1, β-site cleaving enzyme; BACE1-AS, BACE1‐antisense transcript; GAPDH, glyceraldehyde 3-phosphate dehydrogenase; HIF-1α, hypoxia inducible factor-1α; PHD-2, prolyl hydroxylase 2; qPCR, quantitative polymerase chain reaction; SHSY-5Y, neuroblastoma cell line; Tat, transactivator of transcription (TIF) [file pbio.3000660.s020.tif]
