# Supplementary material for: HIV-1 Tat-mediated astrocytic amyloidosis involves the HIF-1α/lncRNA BACE1-AS axis
Source: PLoS Biol. 2020 May 26;18(5):e3000660. doi: 10.1371/journal.pbio.3000660 (PMC7274476; doi:10.1371/journal.pbio.3000660)

**Fig. 1A**

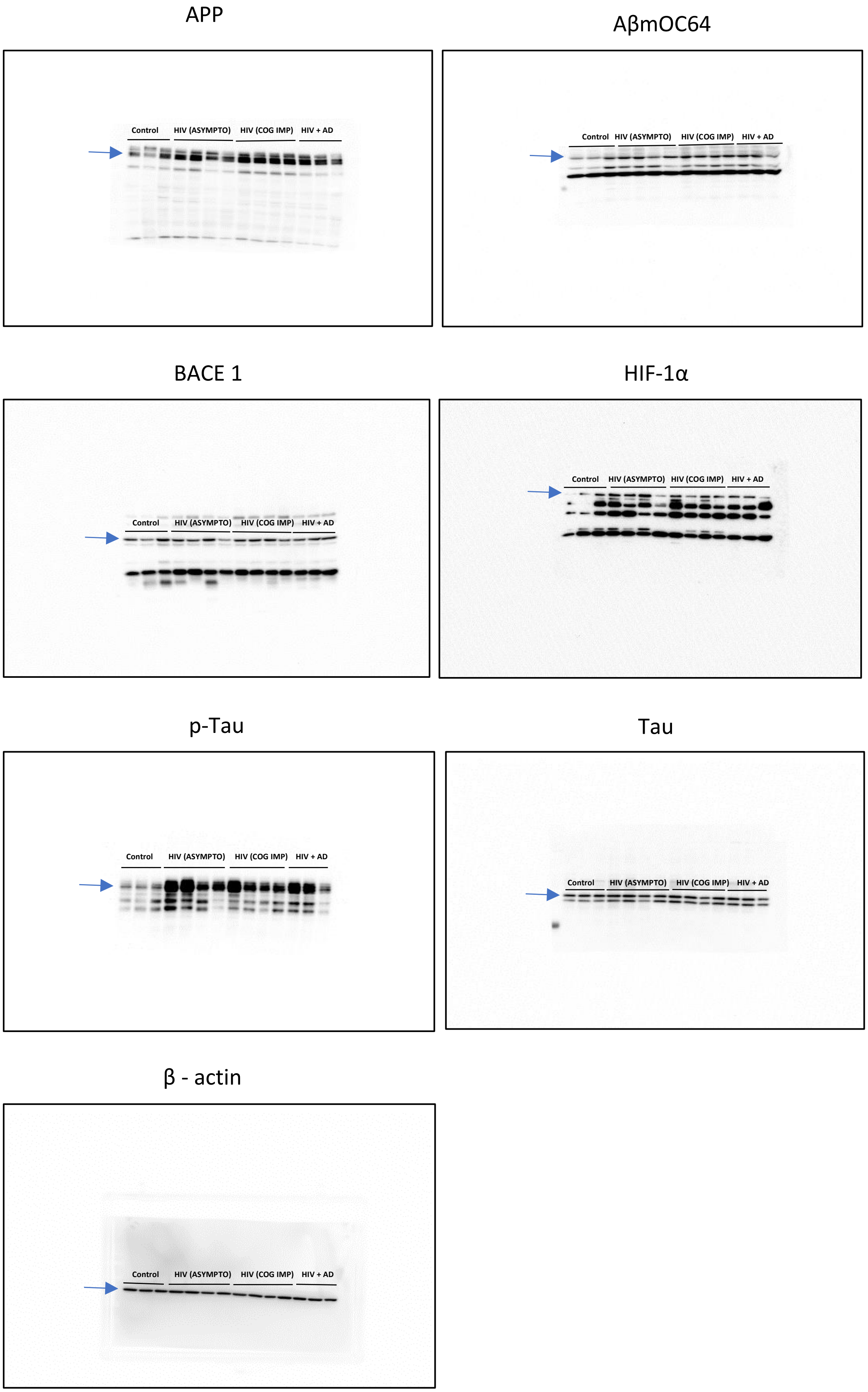
APP
Control
HIV (ASYMPTO)
HIV (COG IMP)
HIV + AD
AβmOC64
Control
HIV (ASYMPTO)
HIV (COG IMP)
HIV + AD
BACE 1
Control
HIV (ASYMPTO)
HIV (COG IMP)
HIV + AD
HIF-1α
Control
HIV (ASYMPTO)
HIV (COG IMP)
HIV + AD
p-Tau
Control
HIV (ASYMPTO)
HIV (COG IMP)
HIV + AD
Tau
Control
HIV (ASYMPTO)
HIV (COG IMP)
HIV + AD
β - actin
Control
HIV (ASYMPTO)
HIV (COG IMP)
HIV + AD

**Fig. 1B**

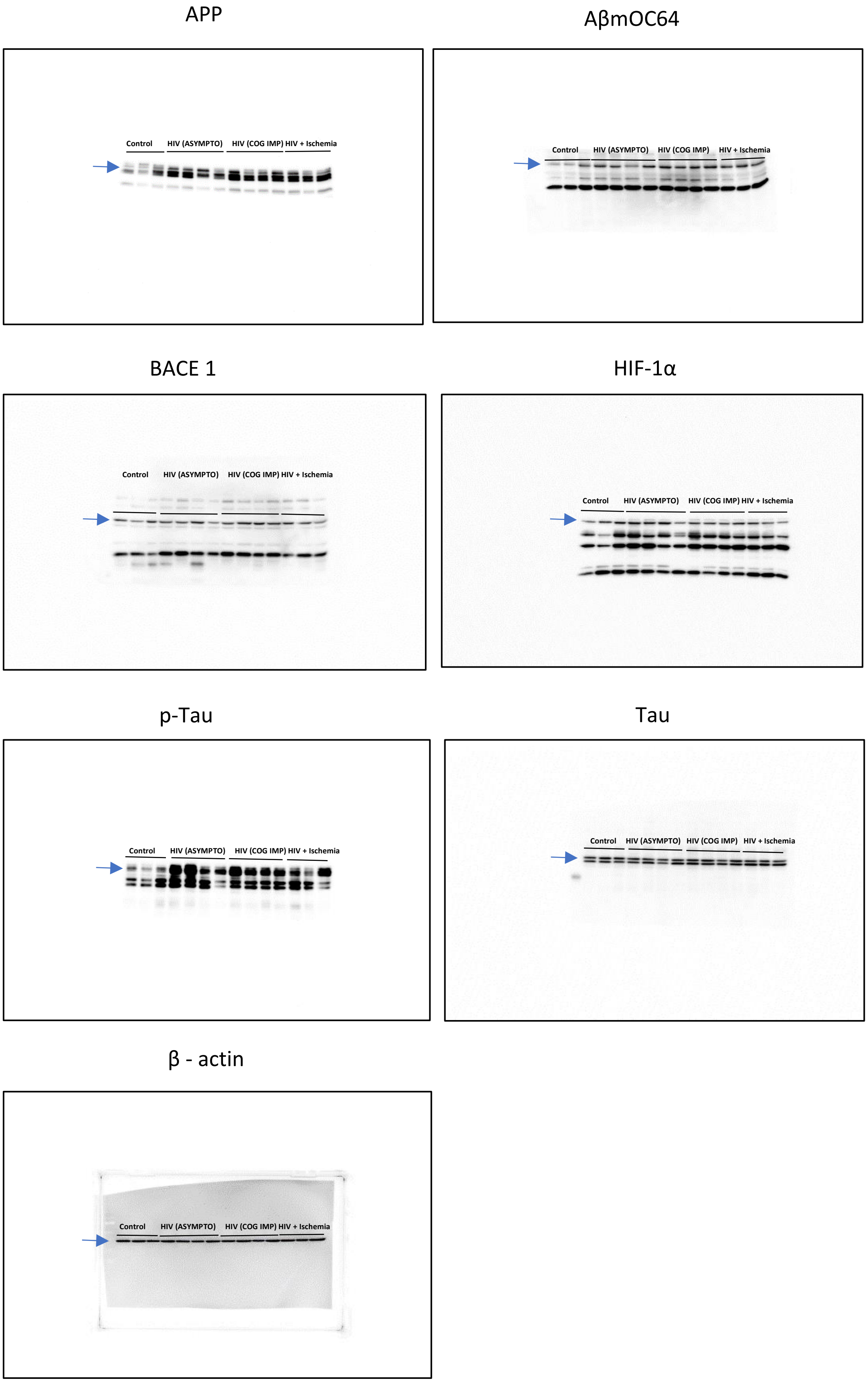
APP
Control
HIV (ASYMPTO)
HIV (COG IMP)
HIV + Ischemia
AβmOC64
BACE 1
HIF-1α
p-Tau
Tau
β - actin

**Fig. 3A – AβmOC64**

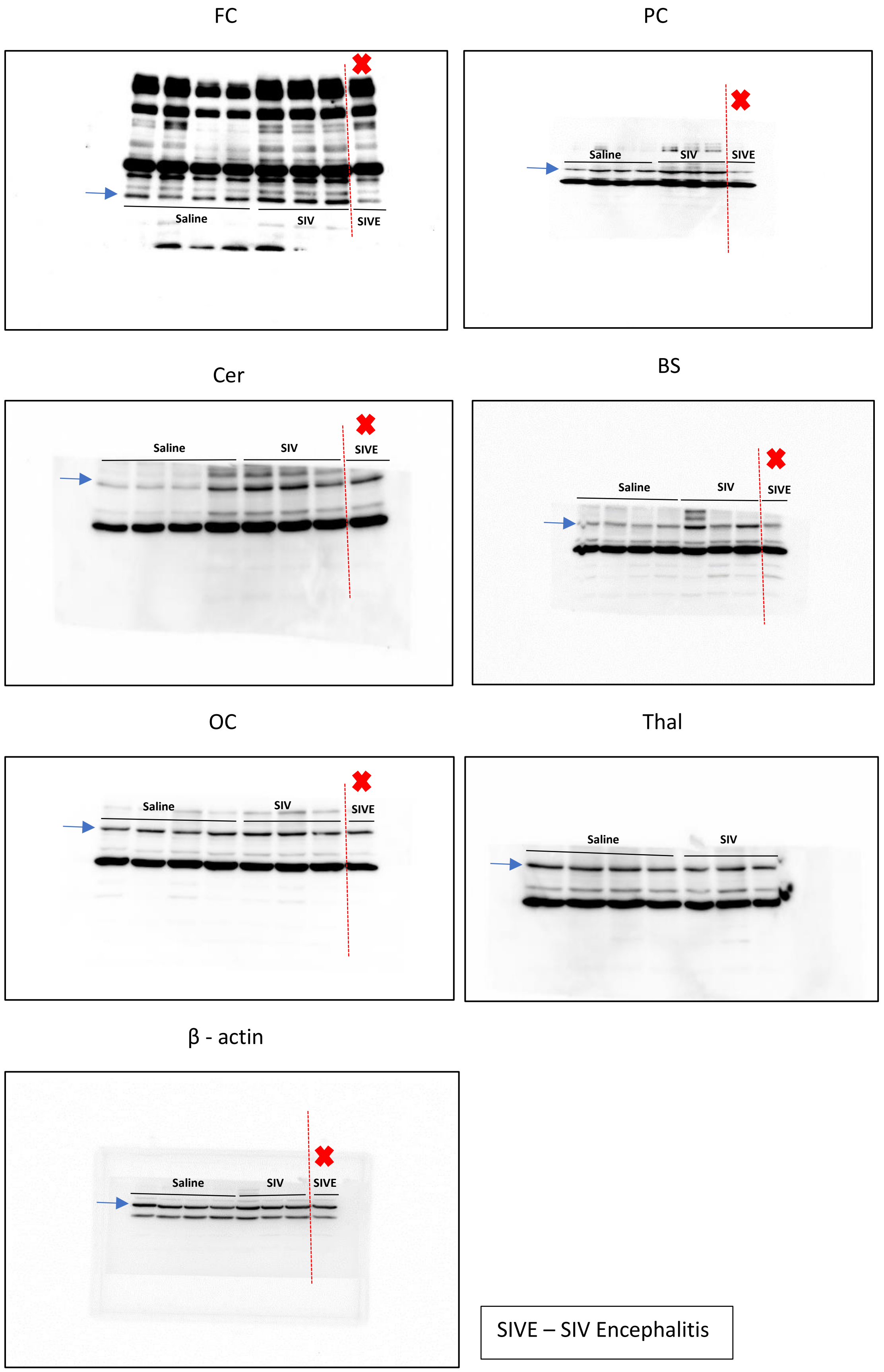

**Fig. 3B – APP**

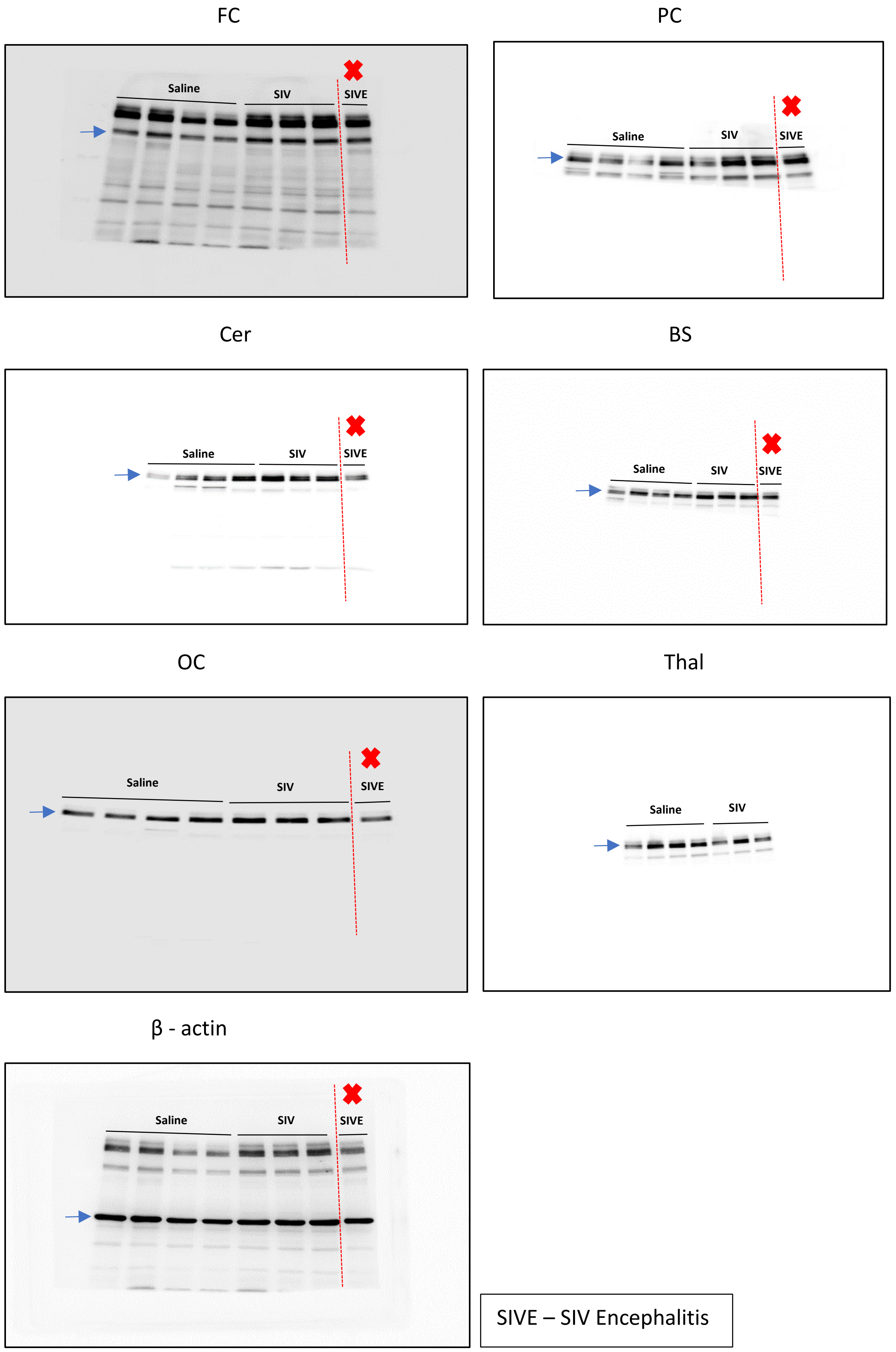
FC
Saline
SIV
SIVE
PC
Saline
SIV
SIVE
Cer
Saline
SIV
SIVE
BS
Saline
SIV
SIVE
OC
Saline
SIV
SIVE
Thal
Saline
SIV
β - actin
Saline
SIV
SIVE
SIVE – SIV Encephalitis

**Fig. 3C – p-Tau/Tau**

FC – p-Tau

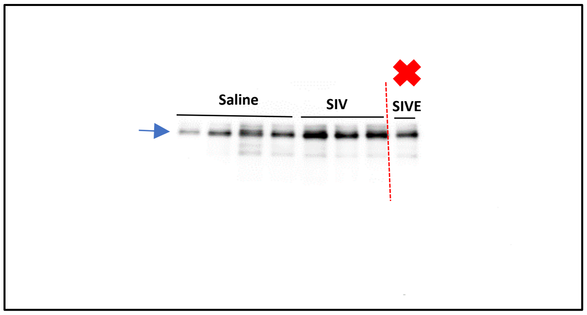


FC- Tau

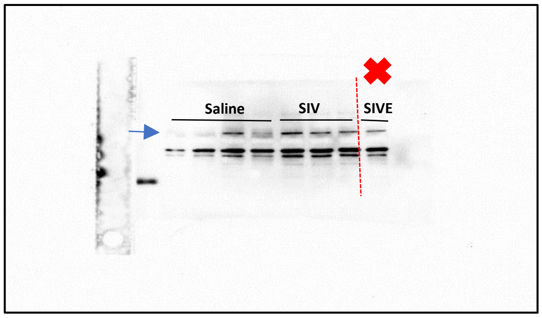


PC- p-Tau

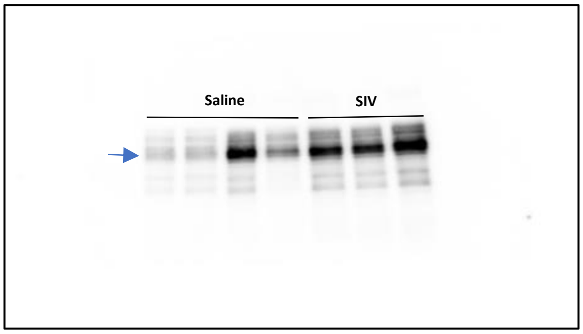


PC-Tau

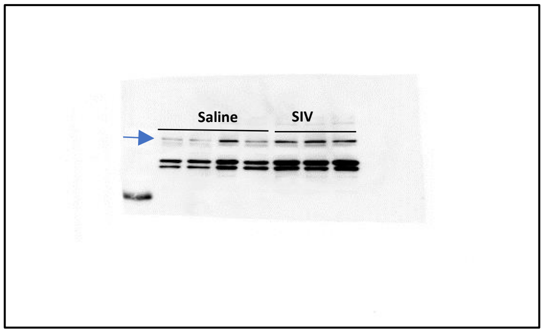


Cer-p-Tau

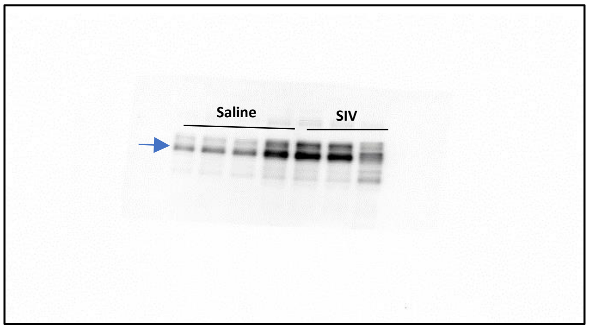


Cer-Tau

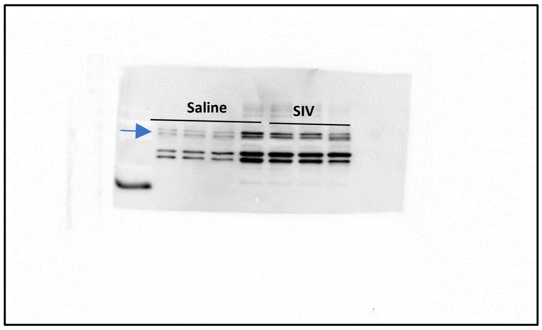


BS-p-Tau

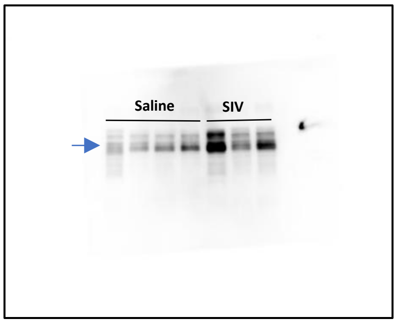


BS-Tau

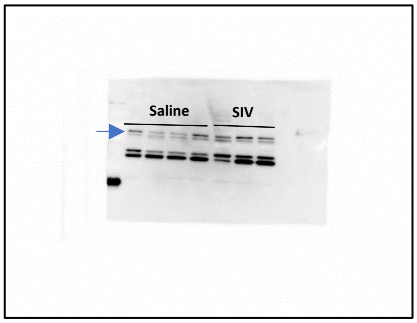


β - actin

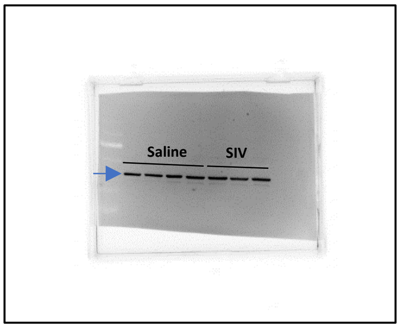


SIVE – SIV Encephalitis

**Fig. 5A**

AβmOC64

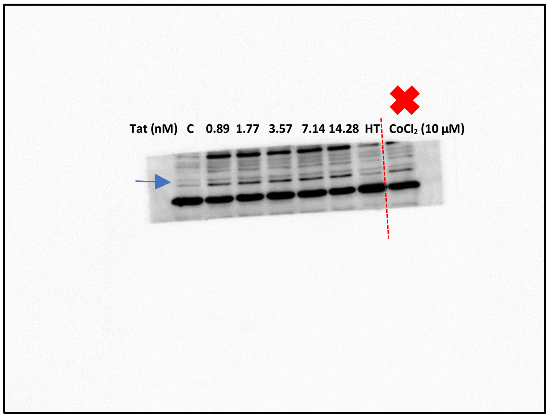


APP

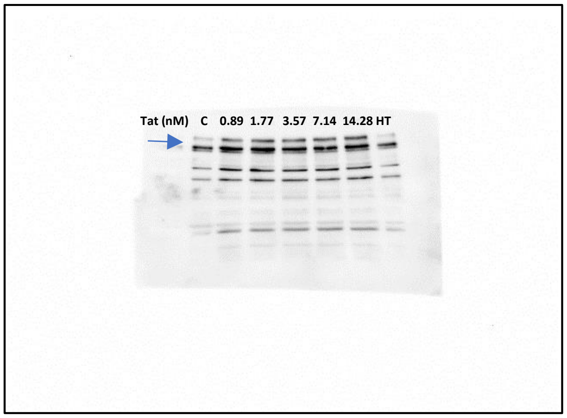


BACE 1

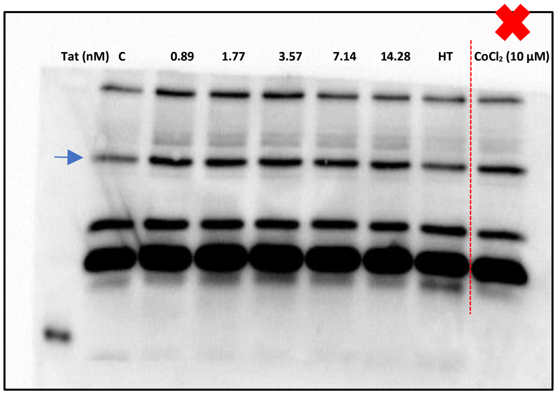


β - actin

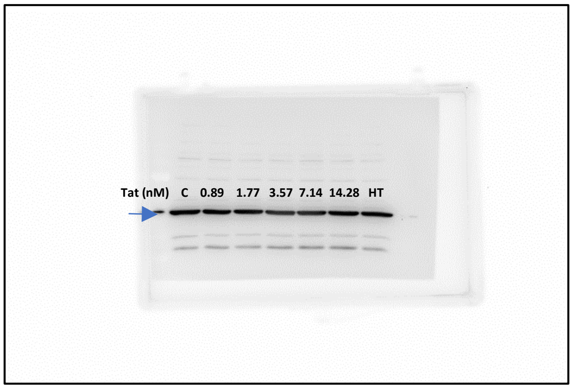

Fig. 5B

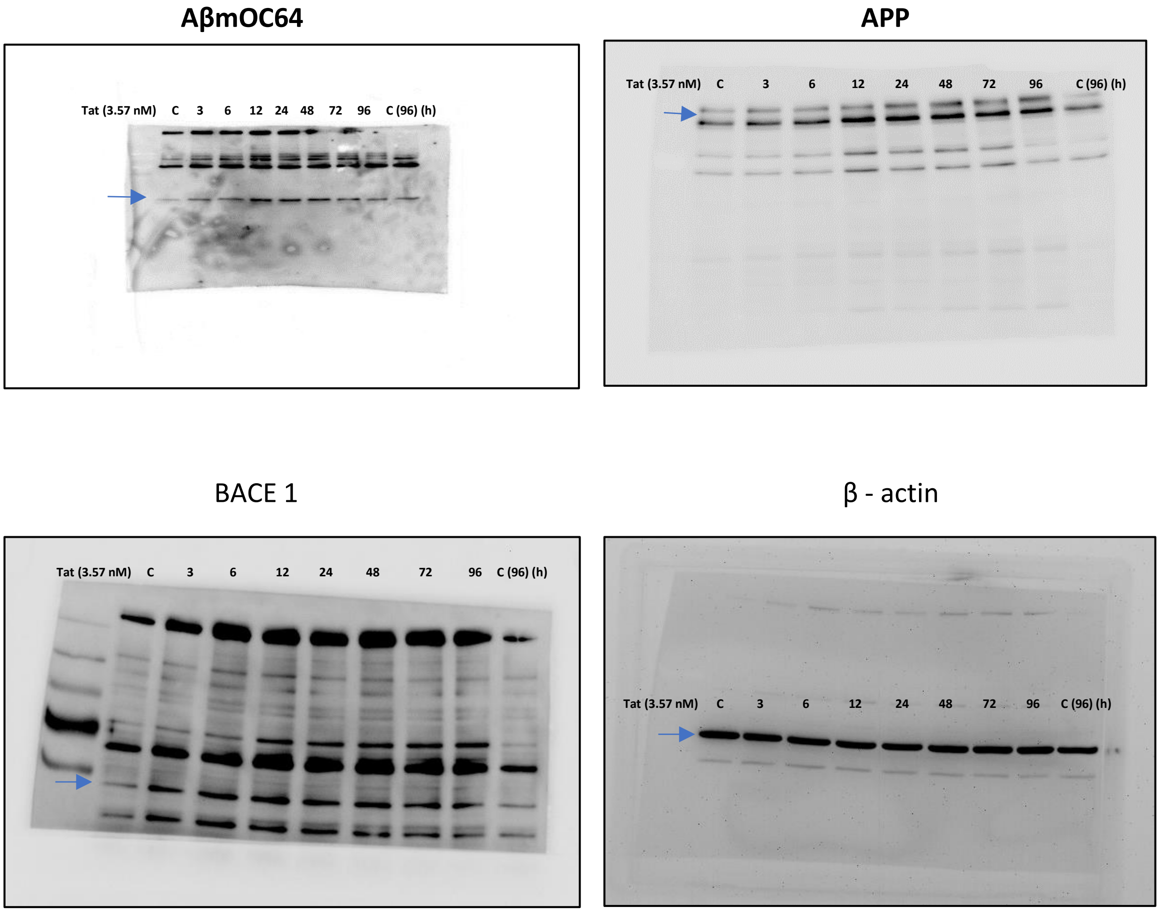
AβmOC64
Tat (3.57 nM) C 3 6 12 24 48 72 96 C (96) (h)
APP
Tat (3.57 nM) C 3 6 12 24 48 72 96 C (96) (h)
BACE 1
Tat (3.57 nM) C 3 6 12 24 48 72 96 C (96) (h)
β - actin
Tat (3.57 nM) C 3 6 12 24 48 72 96 C (96) (h)

**Fig. 6A**

HIF-1α

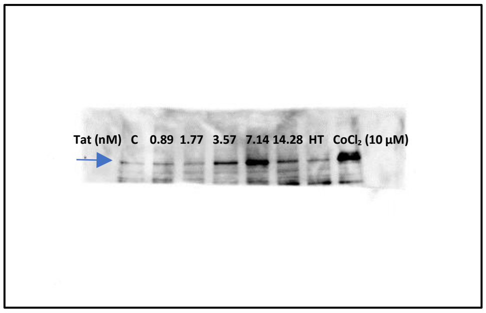


β - actin

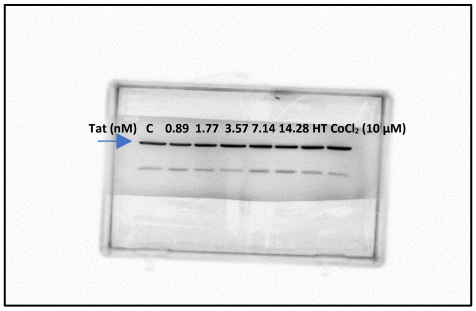


**Fig. 6B**

HIF-1α

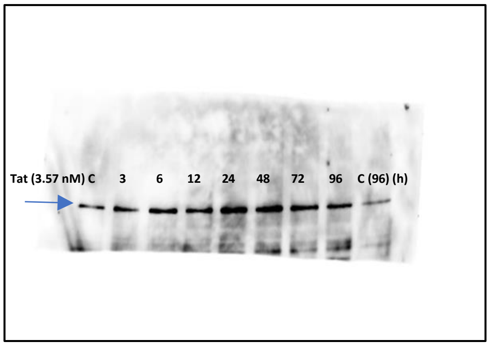


β - actin

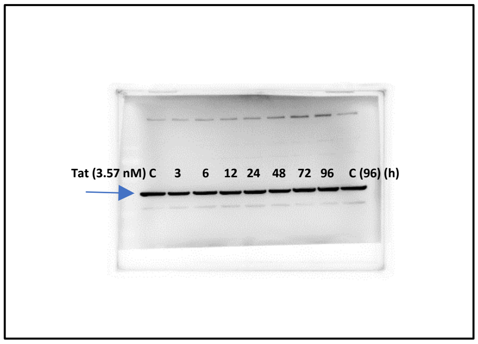


**Fig. 6C**

**Nucleus**

HIF-1α

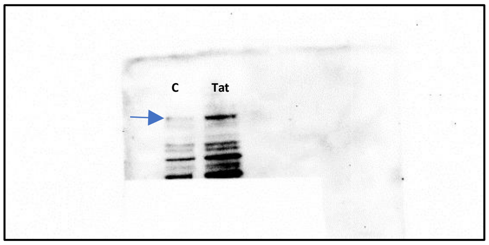


Lamin B1

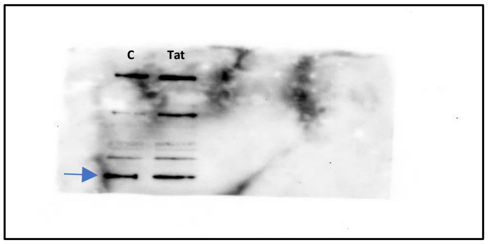


**Cytoplasm**

HIF-1α

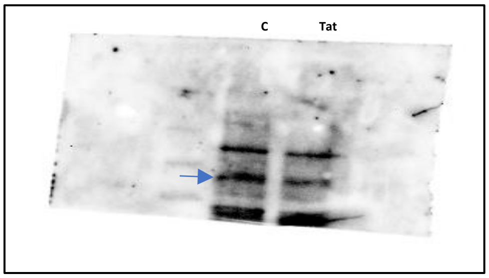


β - actin

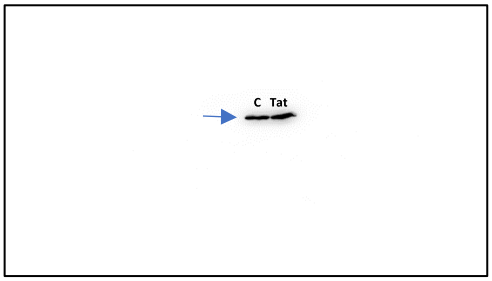

**Fig. 7B**

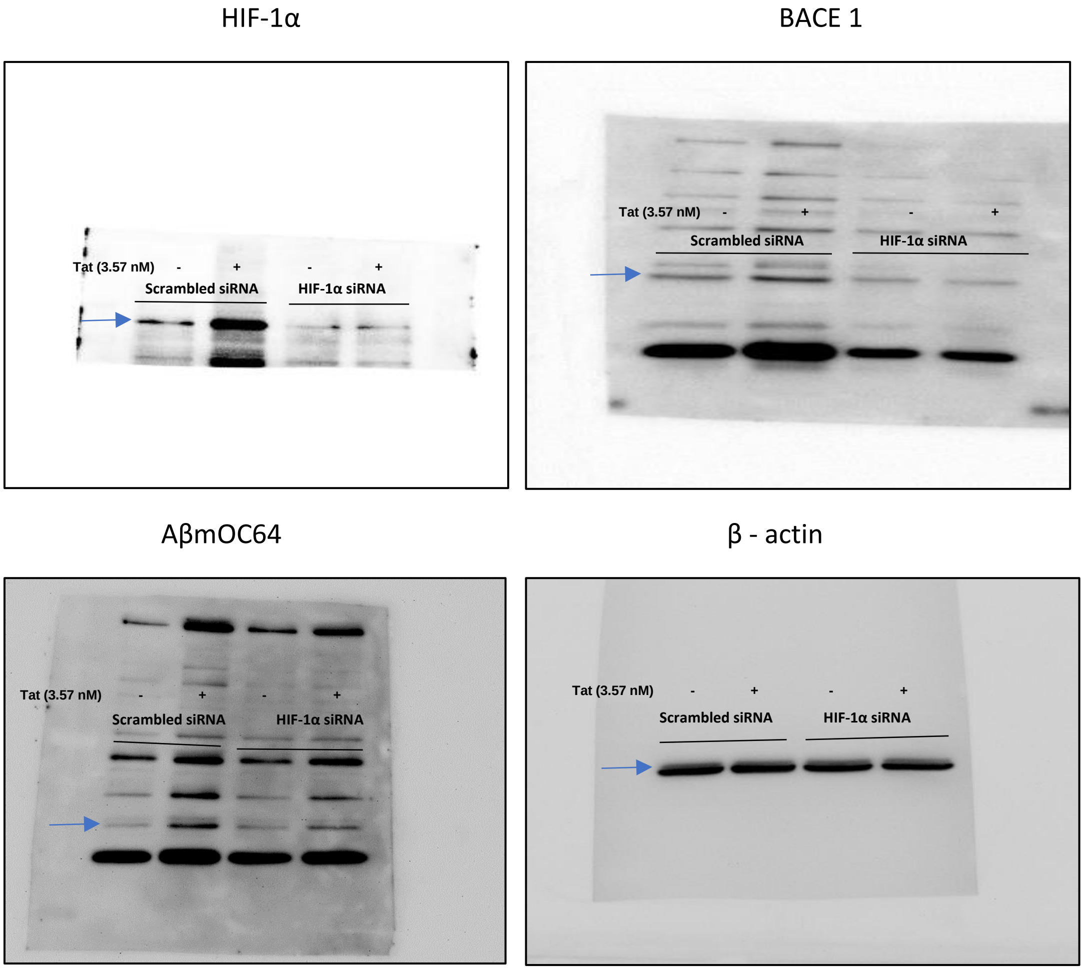

HIF-1α
BACE 1
Tat (3.57 nM) - + - +
Scrambled siRNA
HIF-1α siRNA
AβmOC64
β - actin

**Fig. 7E**

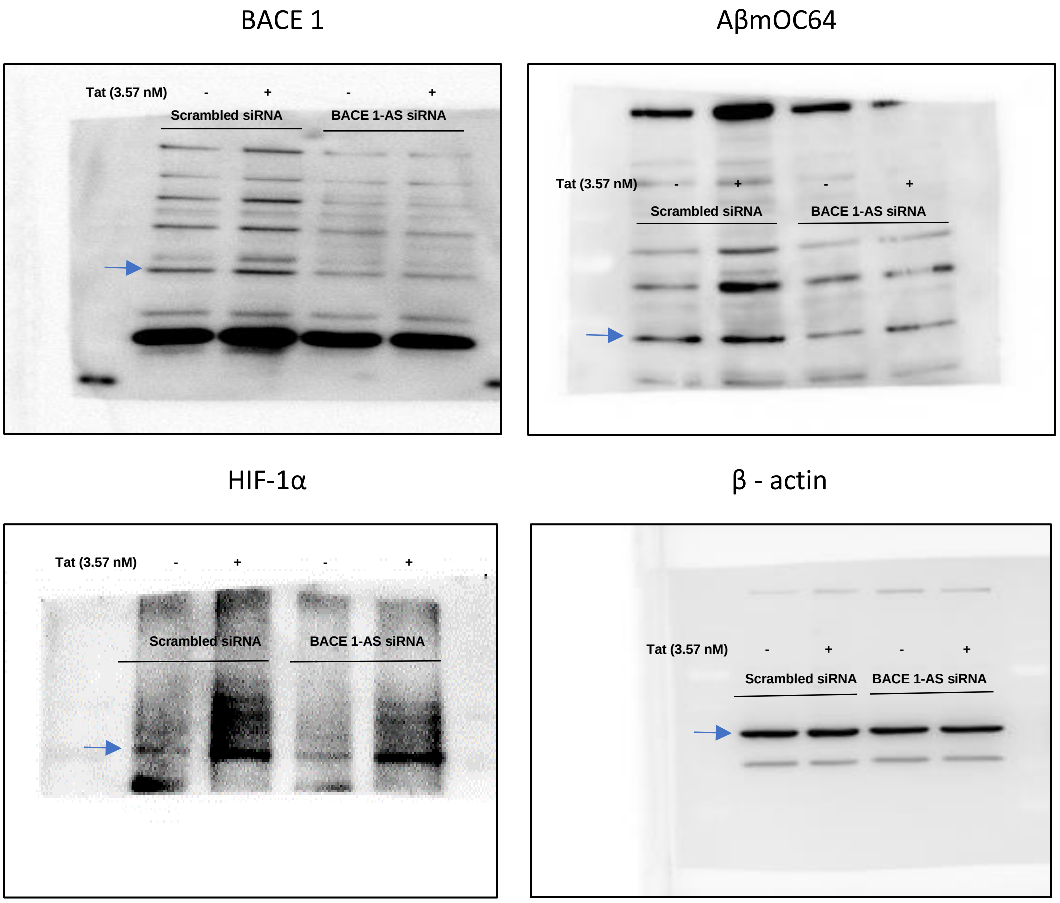
BACE 1
Tat (3.57 nM) - + - +
Scrambled siRNA
BACE 1-AS siRNA
AβmOC64
Tat (3.57 nM) - + - +
Scrambled siRNA
BACE 1-AS siRNA
HIF-1α
Tat (3.57 nM) - + - +
Scrambled siRNA
BACE 1-AS siRNA
β - actin
Tat (3.57 nM) - + - +
Scrambled siRNA
BACE 1-AS siRNA

**Fig. 8B**

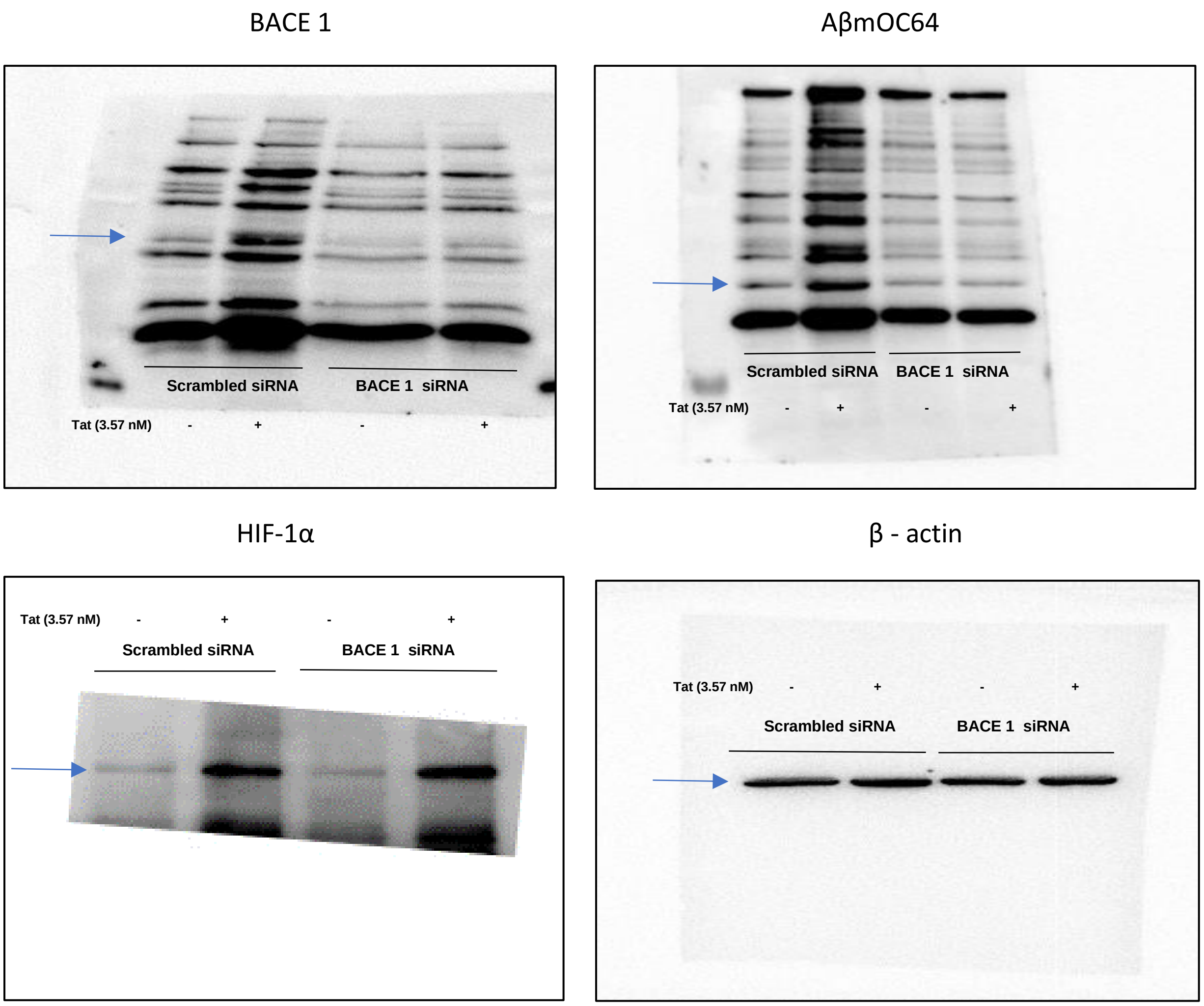
BACE 1
Scrambled siRNA
BACE 1 siRNA
Tat (3.57 nM) - + - +
AβmOC64
Scrambled siRNA
BACE 1 siRNA
Tat (3.57 nM) - + - +
HIF-1α
Tat (3.57 nM) - + - +
Scrambled siRNA
BACE 1 siRNA
β - actin
Tat (3.57 nM) - + - +
Scrambled siRNA
BACE 1 siRNA

**Fig. 8E**

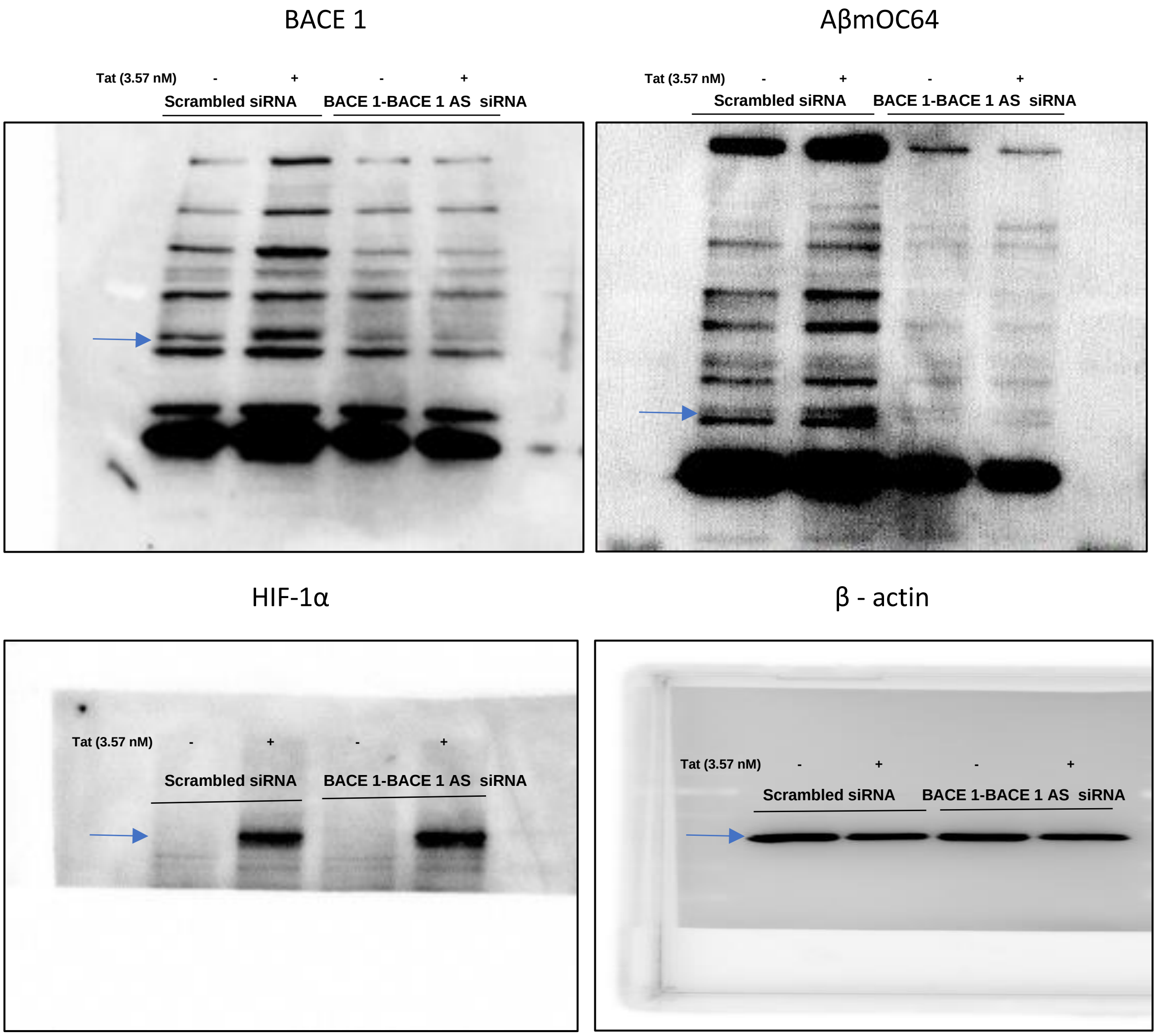
BACE 1
AβmOC64
Tat (3.57 nM) - + - +
Scrambled siRNA
BACE 1-BACE 1 AS siRNA
Tat (3.57 nM) - + - +
Scrambled siRNA
BACE 1-BACE 1 AS siRNA
HIF-1α
β - actin
Tat (3.57 nM) - + - +
Scrambled siRNA
BACE 1-BACE 1 AS siRNA
Tat (3.57 nM) - + - +
Scrambled siRNA
BACE 1-BACE 1 AS siRNA

Fig. 9D

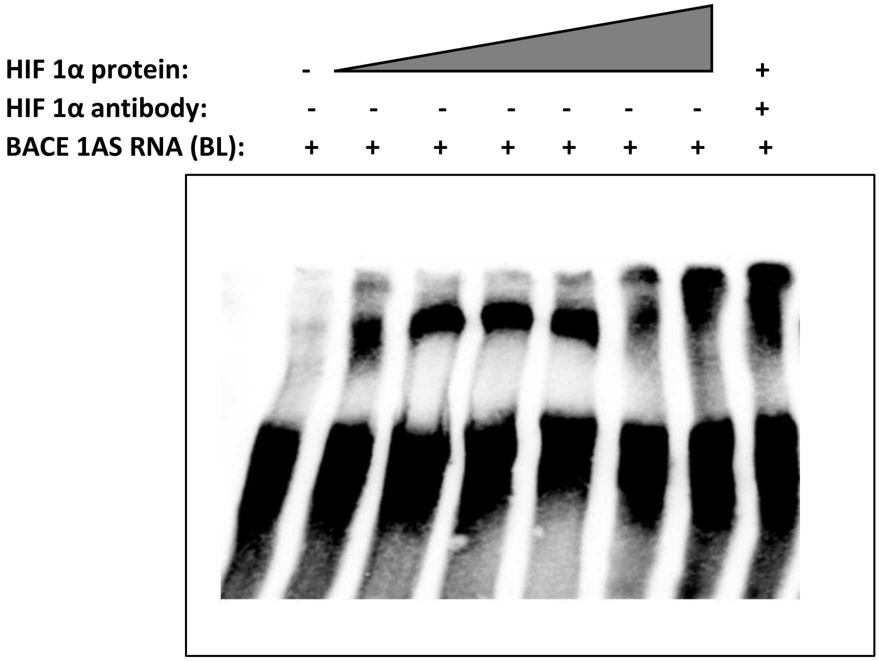

**Fig. 10B**

HIF-1α

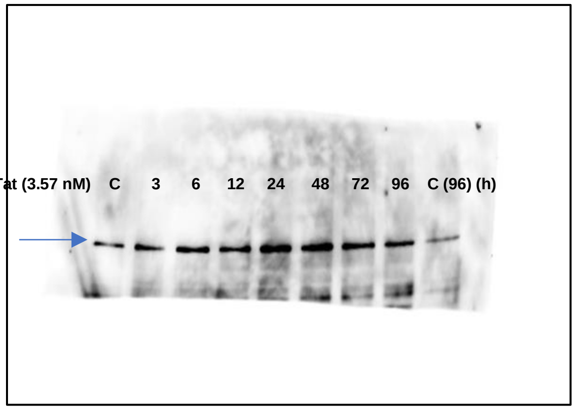


β - actin

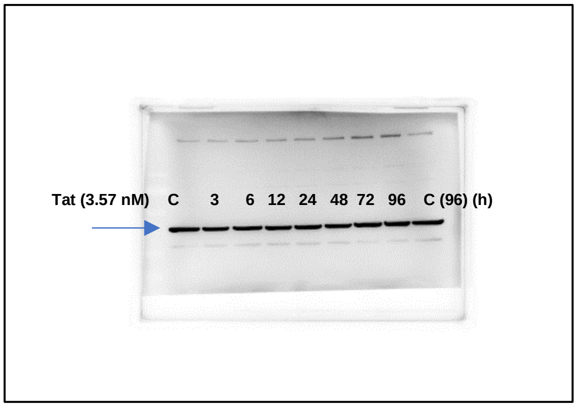


**Fig. 10D**

PHD-2

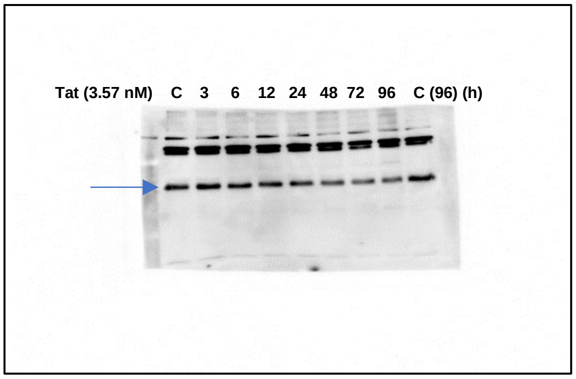


β - actin

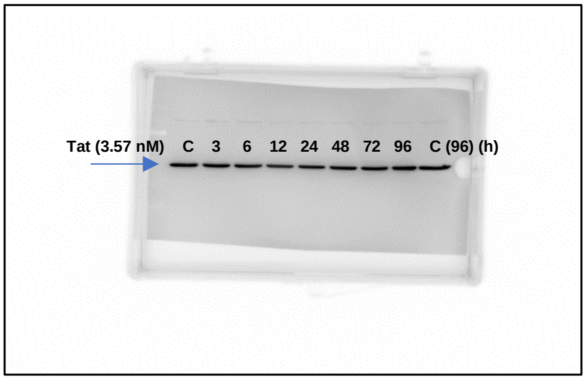

**Supplementary Fig. 2A – HIF-1α**

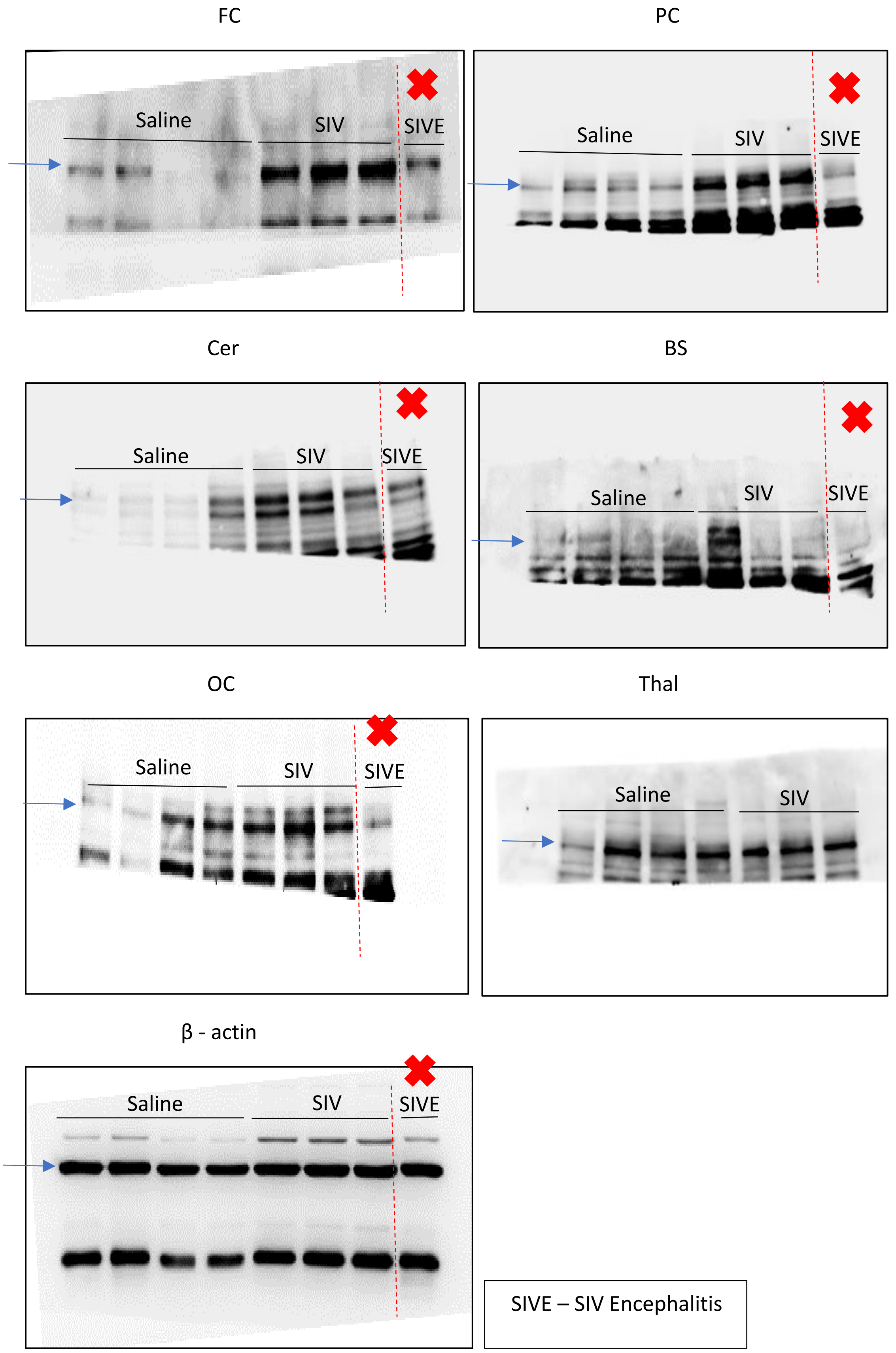

**Supplementary Fig. 2B – BACE 1**

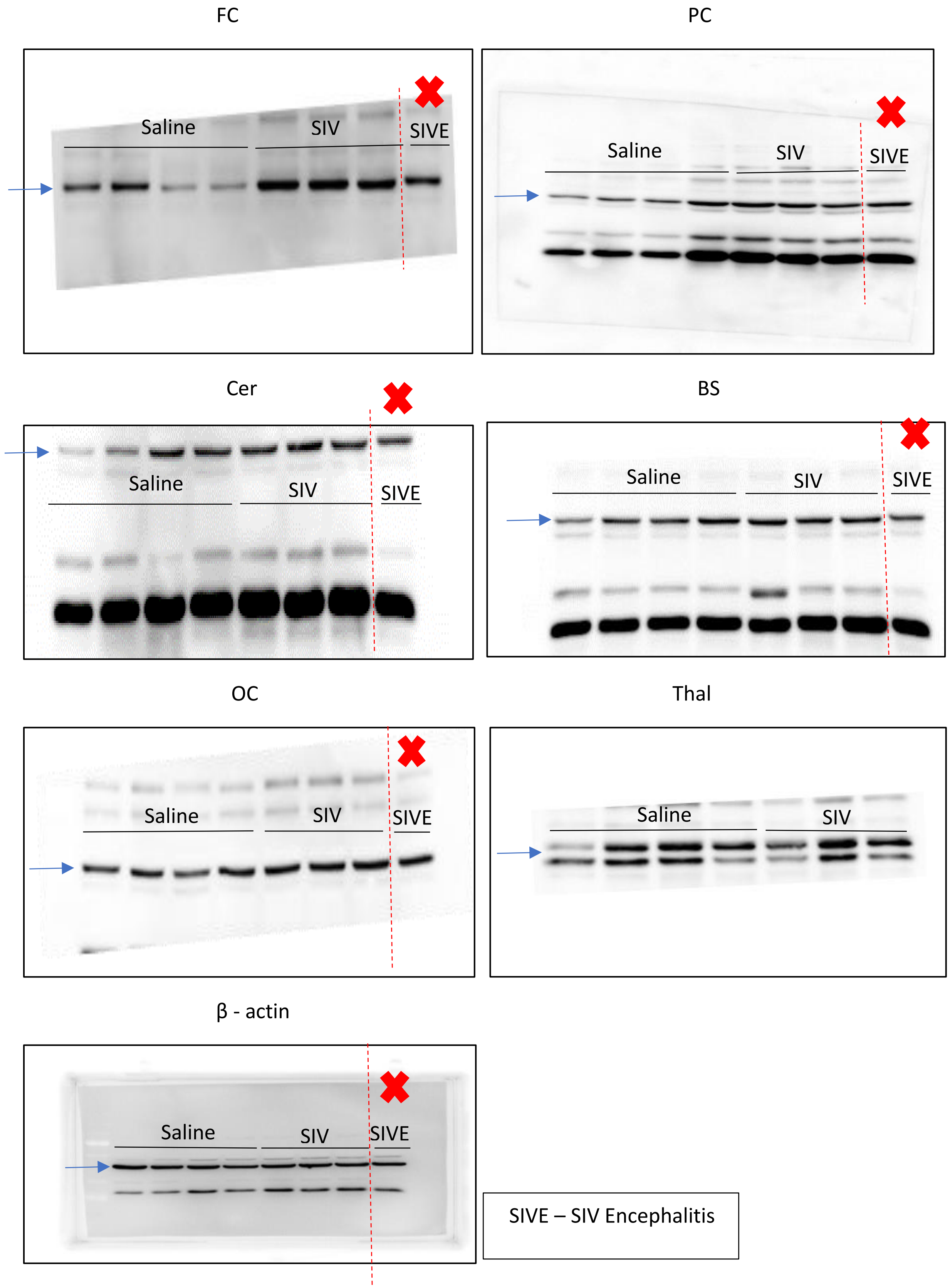

## Supplementary Fig. 7A

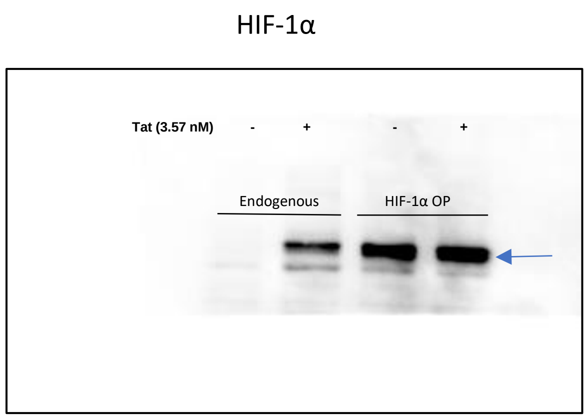


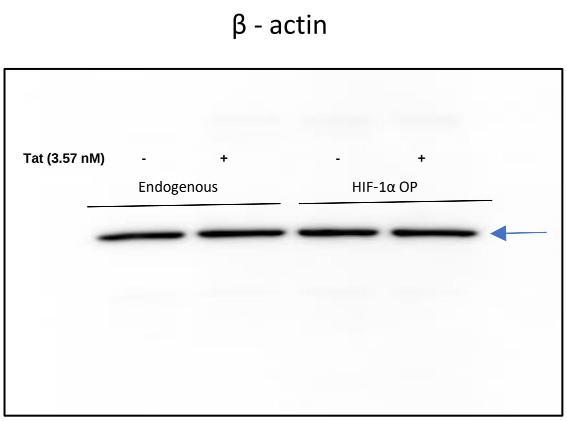

**Supplementary Fig. 8B:**

PHD-2

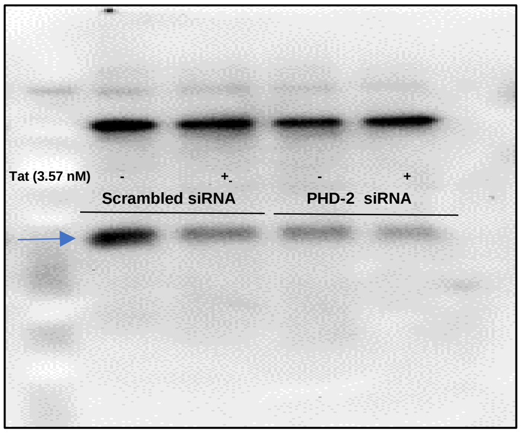


HIF-1α

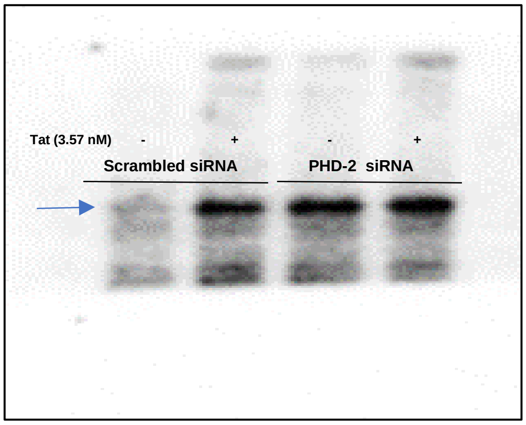


BACE-1

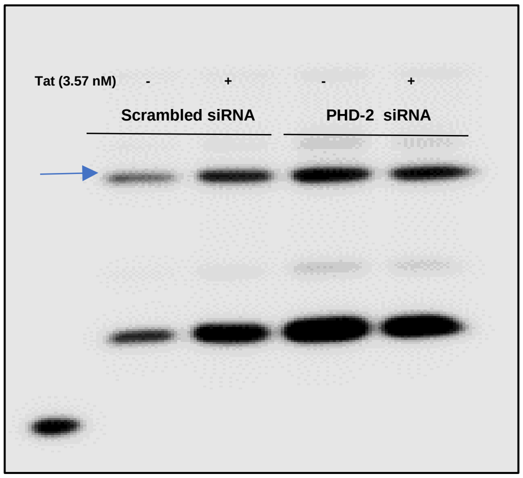


APP

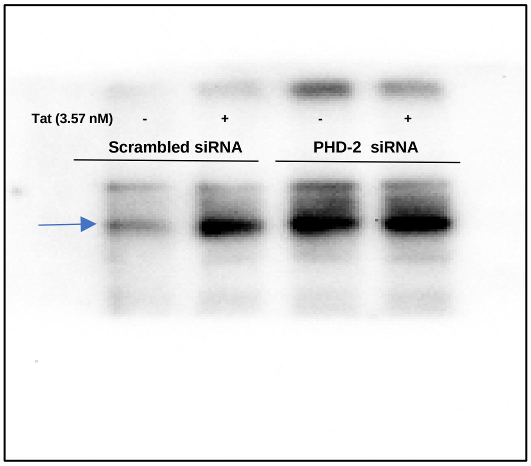


AβmOC64

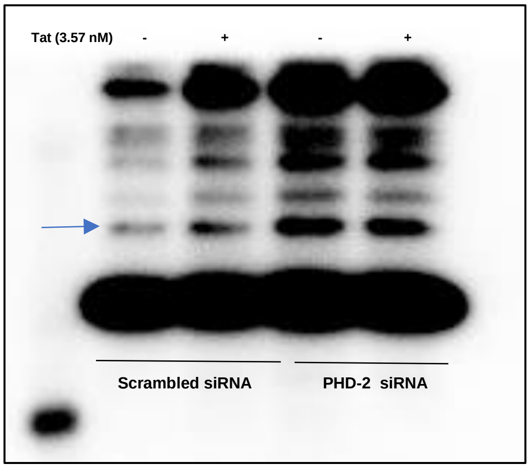


β-actin

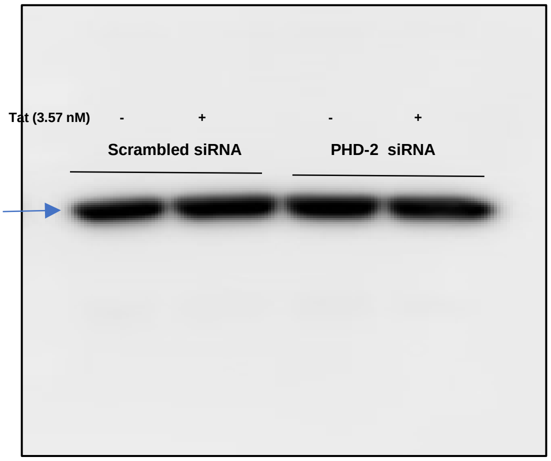

**Supplementary Fig. 8D:**

PHD-2

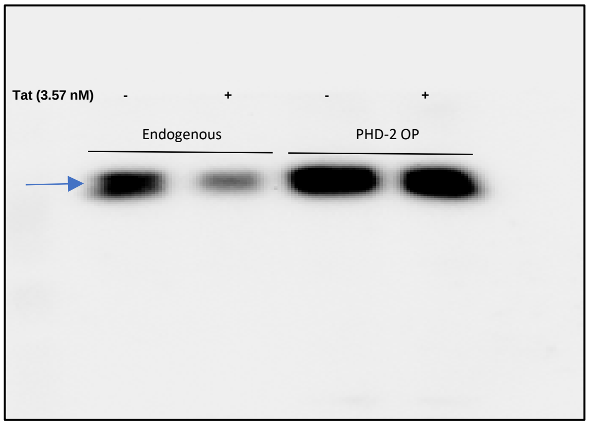


HIF-1α

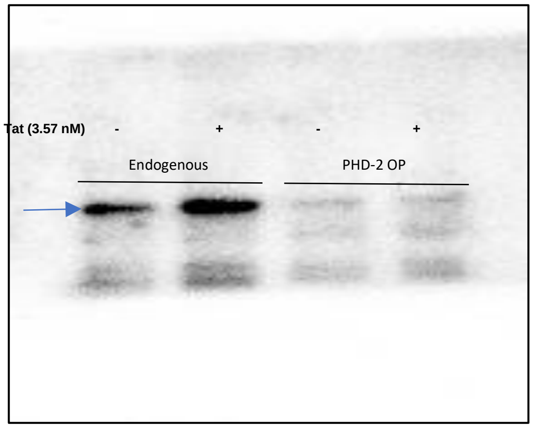


BACE-1

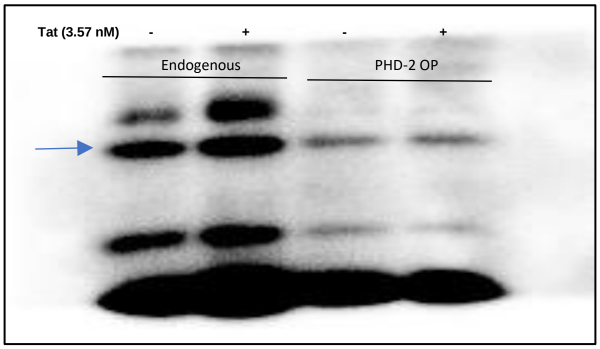


APP

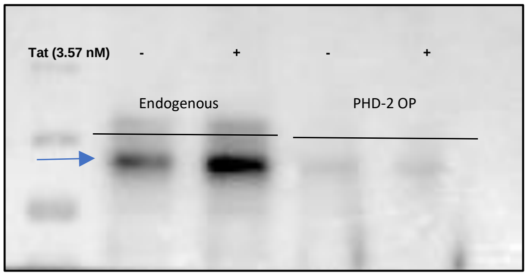


AβmOC64

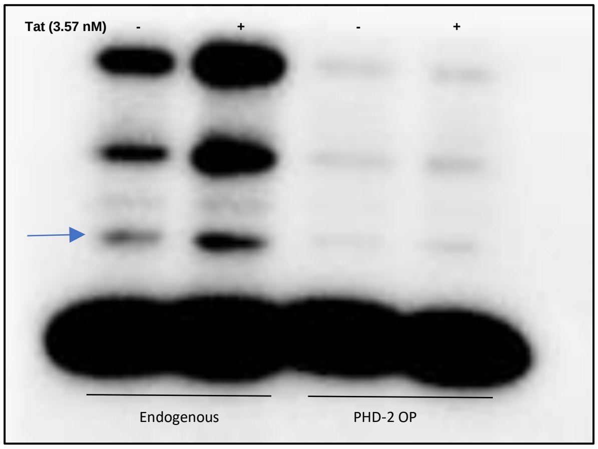


β-actin

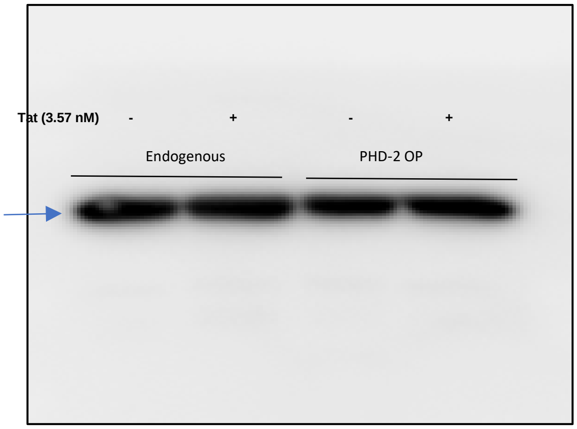

**Supplementary Fig. 10C:**

HIF-1α

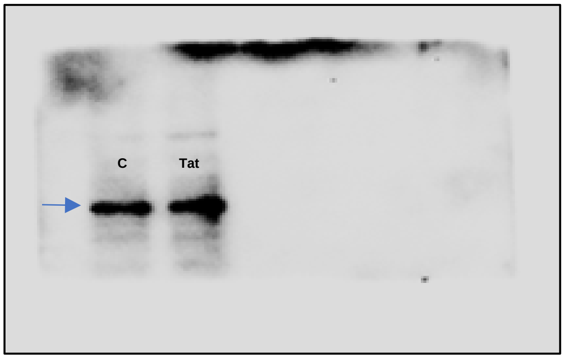


BACE-1

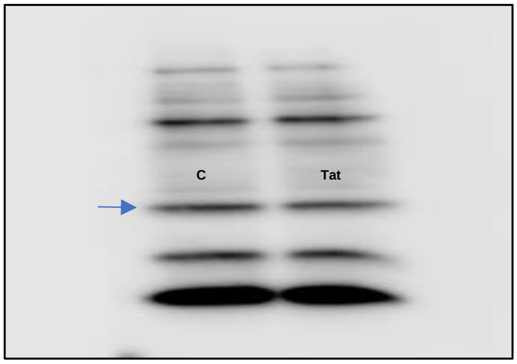


APP

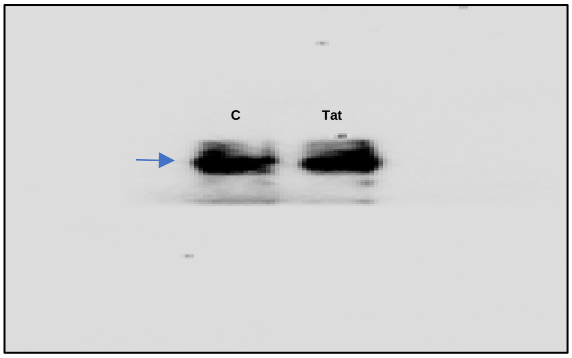


Aβm0C64

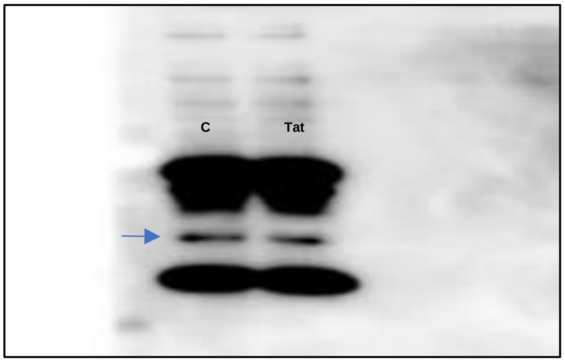


PHD-2

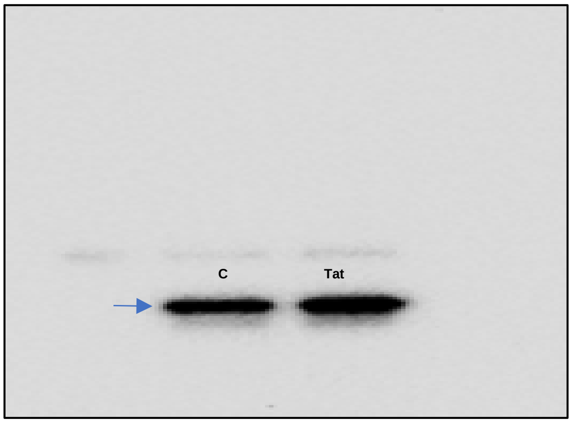


β-actin

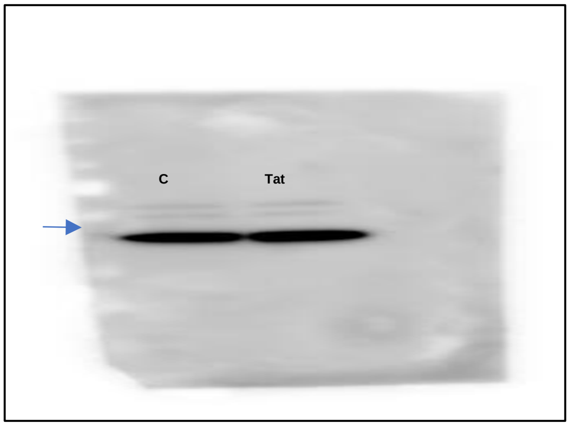

**Supplementary Fig. 10E:**

HIF-1α

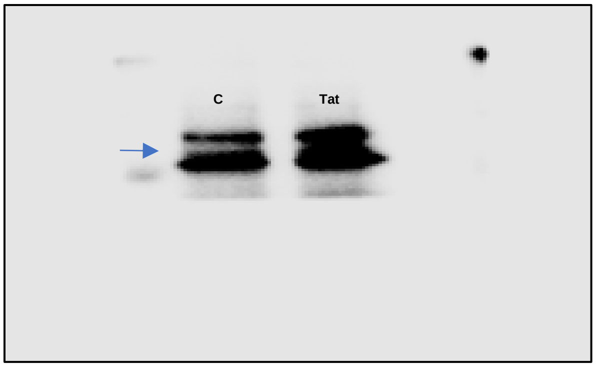


BACE-1

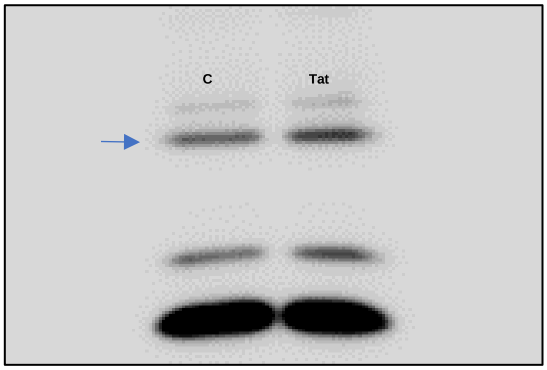


APP

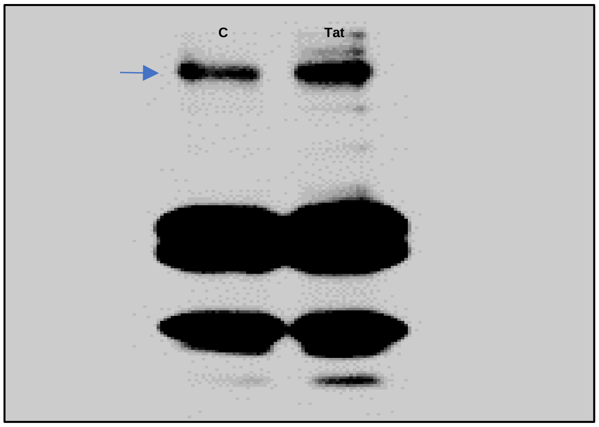


Aβm0C64

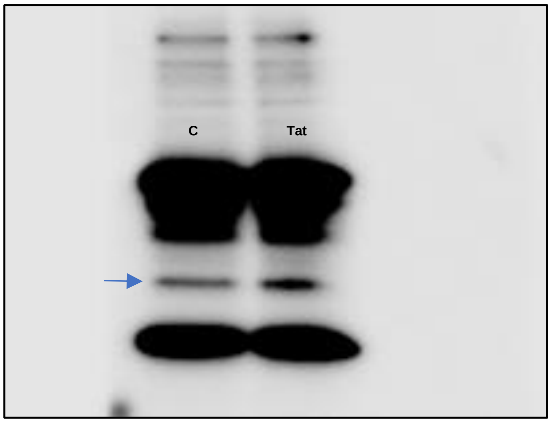


PHD-2

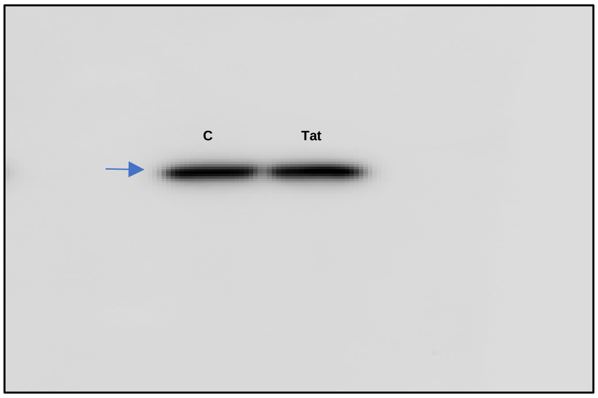


β-actin

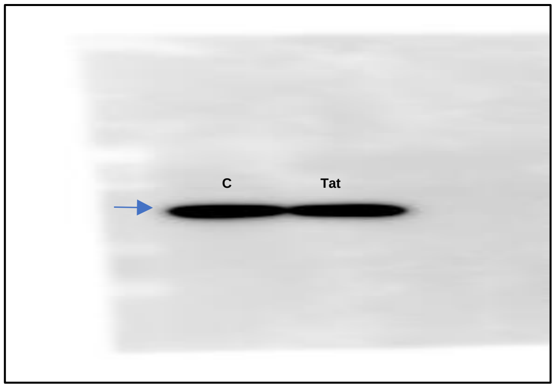

Supplement: S1 Raw Images — (PDF) [file pbio.3000660.s021.pdf]
