# Supplementary material for: HIV-1 Tat-mediated astrocytic amyloidosis involves the HIF-1α/lncRNA BACE1-AS axis
Source: PLoS Biol. 2020 May 26;18(5):e3000660. doi: 10.1371/journal.pbio.3000660 (PMC7274476; doi:10.1371/journal.pbio.3000660)
Supplement: S1 Methods — (DOCX) [file pbio.3000660.s022.docx]

**Supporting Information:**

**Methods and materials:**

**Cell culture**

**Human neurons**

Human primary neurons were obtained from ScienCell Research Laboratories (1520) and were cultured in neuronal media (ScienCell Research Laboratories, 1521) in a 5% CO_2_-humidified incubator at 37°C as per the manufacturer’s protocol. Neurons were plated in 6-well plates at seeding a density of 0.3 × 10^6^/ well.

**Neuroblastoma (SH-SY5Y) cells**

SH-SY5Y cells were plated at a seeding density of 1 × 10^6^ per ml and cultured in 1 : 1 mixture of Eagle’s minimum essential medium containing nonessential amino acids (Gibco, Gaithersburg, MD, USA) and F12 medium (Gibco) supplemented with heat-inactivated fetal bovine serum (10% v/v) and 2 mM glutamine at 37 °C in 5% CO_2_.

**Small interfering RNA (siRNA) transfection**

HPAs were seeded in 6-well plates (0.3×10^6^ cells per well) and incubated overnight at 37°C in a humidified, 5% CO_2_ incubator. Next day, cells were transfected with either human *PHD-2* siRNA (Santa Cruz Biotechnology, sc-45537) or PHD-2 overexpressing plasmid (Addgene, 18963) as described previously [62]. Knockdown efficiencies were confirmed by western blotting and qPCR.

**Phagocytosis assay:**

Phagocytosis assay was performed in control and Tat exposed (24h) HPAs as per the manufacturer’s protocol (Cayman, Item No. 500290). Briefly, to investigate the ability of astrocytes to internalize particles Fitc^+^-GFP beads, HPAs (2 × 10^5^ cells/mL) were plated on a 24-well plate and allowed to adhere overnight, and then incubated with FITC-labeled rabbit IgG-coated latex beads, intensively washed, followed by immunocytochemistry staining with GFAP antibody and imaged with confocal microscope.

**Results:**

**Expression of Aβ 1-42 and co-localization with GFAP in brain regions of SIV-infected macaques:** The brain sections from different brain regions (FC, PC, Hippocampus (Hippo), Basal Ganglia (BG), Cer and BS, were co-immunostained for the expression of Aβ1-42 in GFAP+ astrocytes. As shown in Supplementary Fig. 1, there was increased co localization of Aβ1-42 in GFAP+ astrocytes in all of the brain regions of SIV-infected macaques compared with those of the saline group (S1A-F Figs.). Intriguingly however, within the various brain regions of the SIV+ macaques there appeared to be differential expression of the toxic Aβ1-42 protein. More intense staining of Aβ1-42 appeared to be present in the FC, Hippo and the BG compared to other regions tested.

**Expression of HIF-1α and BACE1 in SIV-infected macaques:** HIF-1α and BACE1 were differentially upregulated in the various brain regions of SIV infected macaques; with only the FC, PC, Cer for HIF-1α and FC, Cer for BACE 1 exhibiting significant upregulation *(*P*<0.05) in the SIV+ group compared with the saline injected macaques (S2A-B Figs.).

**Differential expression of Aβ1-40 and p-Tau in the brains of SIV-infected macaques:** In addition to Aβ1-42, upregulation of Aβ1-40 variety was also observed and found to be differentially expressed in various brain regions of SIV-infected macaques, specifically in the astrocytes (S3 Fig.). In addition to amyloid deposition by astrocytes, we also detected region specific upregulation of neuronal p-Tau in the brains of SIV-infected macaques (S4 Fig.) compared with the uninfected controls.

***In situ* hybridiztion (RNA FISH) of BACE1-AS RNA in the brains of SIV-infected macaques:** Archival FC and Hippo regions of SIV-infected macaques demonstrated increased upregulation of BACE1-AS RNA in the GFAP+ astrocytes compared with the expression in the same brain regions of the uninfected controls (S5A-B Figs.).

***In situ* hybridiztion (RNA FISH) of APP RNA in the brains of SIV-infected macaques:** Archival FC and Hippo regions of SIV-infected macaques demonstrated increased upregulation of APP RNA in the GFAP+ astrocytes as well as MAP^+^ neurons compared with the expression in the same brain regions of the saline (S6A-B Figs.). Quantitative analysis showed significant *(p<0.05) co localization of astrocytes and neurons with APP RNA both in the frontal cortex and hippocampus in SIV compared to saline group (S6C-D Figs.).

**Increased Tat-mediated nuclear translocation of HIF-1α in HIF-1α overexpressing HPA:** As shown in S7A Fig. there was significant *(*P*<0.05) upregulation of HIF-1α in HIF-1α overexpressing HPAs in the presence or absence of Tat compared to that of control. Furthermore, as shown in S7B Fig., in the presence of Tat there was increased nuclear translocation of HIF-1α in HIF-1α overexpressing HPAs, as evidenced by immunofluorescence. Additionally, RNA sequencing of control and Tat exposed astrocytes showed that the mRNA expression of APP, HIF-1α, BACE1, BACE1-AS and GFAP were significantly upregulated *(p<0.05) in Tat treated HPA compared to that of control (S7C Fig.).

**PHD-2 in HIV-1 Tat-mediated amyloidosis**: As shown in S8A-B Figs., exposure of scrambled siRNA transfected HPAs to HIV-1 Tat resulted in significantly *(p<0.05) increased expression of HIF-1α, APP, BACE 1, BACE1-AS and AβmOC64 with concomitantly decreased expression of PHD-2 compared to controls. Furthermore, silencing of PHD-2 resulted in significant increase *(p< 0.05) in expression of HIF-1, APP, BACE 1 mRNAs and BACE1-AS by real-time RT-PCR. Western blot showed significant upregulation *(p< 0.05) of HIF-1α, APP, AβmOC64 and BACE 1 proteins (S8B Fig.), with or without exposure to Tat compared to scrambled-siRNA transfected astrocytes (control).

To further validate the role PHD-2 in amyloidosis, HPAs were transfected with PHD-2 overexpressing plasmid. As shown in S8C Figs., overexpression of PHD-2, resulted in significant decrease #(p< 0.05) in expression of HIF-1α, APP, BACE 1 mRNAs and BACE1-AS by real-time RT-PCR. Western blotting showed significant downregulation #(p< 0.05) in expression of HIF-1α, APP, AβmOC64 and BACE 1 proteins (S8D Fig.), with or without Tat exposure compared to Tat-exposed astrocytes, thus showing the intriguing role of PHD-2 in HIV-1 Tat mediated astrocytic amyloidosis.

**Phagocytic activity of HIV-1 Tat exposed HPAs:** Phagocytosis assay showed that HIV-1 Tat exposed HPAs in culture did not phagocytose any Fitc+ beads confirming no alteration in the phagocytic activity of astrocytes in presence or absence of Tat (S9 Fig.). So, this depicts that the Aβ 42 observed in GFAP^+^ astrocytes is not due to phagocytosis of external amyloids, but are produced by the astrocytes.

**Amyloidosis in HIV-1 Tat exposed neurons:** Exposure of human primary neurons to HIV-1 Tat (3.57 nM) for 24h failed to demosntrate an increase in HIF-1α, BACE1, BACE1-AS, APP mRNAs compared with control cells (S10A Fig.). These findings were also valdiated in neuroblastoma cells (SHY-5Y) for both the protein & mRNA expression (S10B-C Fig.). At 48 hrs post HIV-1 Tat exposure there was a significant increase *(p<0.05) in APP, BACE1 mRNAs and APP, Aβm0C64, BACE1 proteins, with no change in mRNA or protein expression of HIF-1α and PHD-2 (S10D-E Fig.) indicating thus that HIV-1 Tat mediated neuronal amyloidosis is HIF-1α independent.

**Figure legends:**

**S1 Fig.: Brain region specific expression of Aβ1-42 in SIV-infected macaques.** Representative fluorescent photomicrographs showing differential expression of Aβ 1-42 in GFAP+ astrocytes in the different brain regions of saline and SIV^+^ macaques. Scale bar: 10 µm. Saline, n = 4, SIV, n = 3.

**S2 Fig.: Expression of HIF-1α and BACE1 protein in the brain regions of SIV-infected macaques.** (A) Representative western blots showing the expression of HIF-1α in different brain regions – Frontal Cortex (FC), Parietal Cortex (PC), Cerebellum (Cer), Brain Stem (BS), Occipital Cortex (OC), Thalamus (Thal) of saline and SIV-infected macaques. (B) Representative western blots showing the expression of BACE1 in different brain regions – FC, PC, Cer, BS, OC and Thal of saline and SIV-infected macaques. β-actin was used as an internal control. n = 6. Data are presented as mean ± SEM; saline n=4, SIV n=3. S2 Fig. data in Supporting Information.

**S3 Fig.: Brain region specific expression of Aβ1-40 in SIV-infected macaques.** Representative fluorescent photomicrographs showing differential expression of Aβ1-40 co-immunostained with GFAP+ astrocytes in the Frontal Cortex (A), Parietal Cortex (B), Hippocampus (C), Basal Ganglia (D), Cerebellum (E) of saline and SIV-infected macaques. n = 4. Scale bar: 10 µm.

**S4 Fig.: Brain region specific expression of p-Tau in SIV-infected macaques.** Representative fluorescent photomicrographs showing differential expression of p-Tau co-immunostained with MAP2+ neurons in the Frontal Cortex (A), Parietal Cortex (B), Hippocampus (C), Basal Ganglia (D), Cerebellum (E) of saline and SIV-infected macaques. n = 4. Scale bar: 10 µm.

**S5 Fig.: Expression of BACE1-AS RNA in various brain regions of SIV-infected macaques by *in situ* hybridization.** Representative FISH and IF photomicrographs showing differential expression of BACE1-AS RNA co-immunostained with GFAP+ astrocytes or MAP2+ neurons in the frontal cortex (A) and hippocampus (B) of saline and SIV-infected macaques. Scale bar: 10 µm. n = 4. Arrows indicate GFAP positive astrocytes co-localized with BACE1-AS RNA.

**S6 Fig.: Expression of APP RNA in various brain regions of SIV-infected macaques by *in situ* hybridization.** Representative FISH and IF photomicrographs in the frontal cortex showing differential expression of APP RNA co-immunostained with GFAP+ astrocytes or MAP2+ neurons (A) and quantative analysis of percent of GFAP+ astrocytes or MAP2+ neurons co-localized with APP RNA in the frontal cortex (B). Representative FISH and IF photomicrographs in the hippocampus showing differential expression of APP RNA co-immunostained with GFAP+ astrocytes or MAP2+ neurons (C) and quantative analysis of percent of GFAP+ astrocytes or MAP2+ neurons co-localized with APP RNA in the hippocampus (D). Scale bar: 10 µm. n = 4. Data are presented as mean ± SEM; n = 3. Student's t-test was used to determine the statistical significance between two groups: * P < 0.05 vs. control. S6 Fig. data in Supporting Information.

**S7 Fig.: HIV-1 Tat mediated nuclear translocation of HIF-1α in HIF-1α overexpressing HPAs.** (A) Representative western blot showing expression of HIF-1α in endogenous and overexpressing HPA in the presence or absence of HIV-1 Tat (3.57 nM). (B) Representative fluorescent photomicrographs showing increased expression of HIF-1α in HIF-1α overexpressing HPAs in the presence or absence of HIV-1 Tat (3.57 nM; 30 min). Scale bar: 10 µm. (C) RNA seq data representing heatmaps for dysregulated genes – APP, HIF-1α, BACE1, BACE1-AS and GFAP in control or Tat exposed HPAs. (D) RNA sequencing data of the HIF-1α bound RNA complexes (RIP assay). Data are presented as mean ± SEM; n = 6. One-way ANOVA followed by Bonferroni *post hoc* test was used to determine the statistical significance between multiple groups: * P < 0.05 vs. control. S7 Fig. data in Supporting Information.

**S8 Fig.: HIV-1 Tat-mediated regulation of PHD-2 in modulating astrocytic amyloidosis.** (A) qPCR analysis demonstrating expression of PHD-2, HIF-1α, BACE1-AS, BACE1 and APP mRNAs in HPAs transfected with either PHD-2/ scrambled siRNA in the presence or absence of HIV-1 Tat (3.57 nM; 24h), (B) Representative western blots showing the expression of PHD-2, HIF-1α, BACE1, APP and Aβ mOC64 proteins in HPAs transfected with either PHD-2 or scrambled siRNA, (C) qPCR analysis demonstrating expression of PHD-2, HIF-1α, BACE1-AS, BACE1 and APP mRNAs in HPAs transfected with PHD-2 overexpressing plasmid in the presence or absence of HIV-1 Tat (3.57 nM; 24h), (D) Representative western blots showing the expression of PHD-2, HIF-1α, BACE1, APP and Aβ mOC64 proteins in HPAs transfected with PHD-2 overexpressing plasmid in the presence or absence of HIV-1 Tat (3.57 nM; 24h). Data are presented as mean ± SEM; n = 6. One-way ANOVA followed by Bonferroni *post hoc* test was used to determine the statistical significance: * P < 0.05 vs. control, ^#^ P < 0.05 vs. Tat. S8 Fig. data in Supporting Information.

**S9 Fig.: Phagocytic activity in HIV-1 Tat exposed HPAs:** Representative immunocytochemistry images showing the phagocytic potential of GFAP+ HPAs in control and Tat exposed cells (3.57 nM; 24h). Scale bar: 10 µm. n = 6.

**S10 Fig.: HIV-1 Tat-mediated amyloidosis in human neurons.** qPCR analysis showing expression of APP, BACE1, BACE1-AS, HIF-1 and PHD-2 mRNAs in human primary neurons exposed to HIV-1 Tat (3.57 nM, 24h). GAPDH was used as an internal control for mRNA expression (A). qPCR analysis showing expression of APP, BACE1, BACE1-AS, HIF-1 and PHD-2 mRNAs in SHSY-5Y cells exposed to HIV-1 Tat (3.57 nM, 24h). GAPDH was used as an internal control for mRNA expression (B). Western blot analysis showing expression of HIF-1α, BACE 1, APP, Aβ mOC64 and PHD-2 proteins in SHSY-5Y cells exposed to HIV-1 Tat (3.57 nM, 24h). β-actin was used as an internal control (C). qPCR analysis showing expression of APP, BACE1, BACE1-AS, HIF-1 and PHD-2 mRNAs in SHSY-5Y cells exposed to HIV-1 Tat (3.57 nM, 48h). GAPDH was used as an internal control for mRNA expression (D). Western blot analysis showing expression of HIF-1α, BACE 1, APP, Aβ mOC64 and PHD-2 proteins in SHSY-5Y cells exposed to HIV-1 Tat (3.57 nM, 48h). β-actin was used as an internal control (E). Data are presented as mean ± SEM; n = 6. Student’s t-test was used to determine the statistical significance: * P < 0.05 vs. control. S10 Fig. data in Supporting Information.
